# pyRootHair: Machine learning accelerated software for high-throughput phenotyping of plant root hair traits

**DOI:** 10.1093/gigascience/giaf141

**Published:** 2025-11-13

**Authors:** Ian Tsang, Lawrence Percival-Alwyn, Stephen Rawsthorne, James Cockram, Fiona Leigh, Jonathan A Atkinson

**Affiliations:** Plant Genetics Department, Niab, Park Farm, Cambridge CB24 9NZ, UK; Plant Sciences Building, Sutton Bonnington Campus, University of Nottingham, Nottingham LE12 5RD, UK; Plant Genetics Department, Niab, Park Farm, Cambridge CB24 9NZ, UK; The Morley Agricultural Foundation, Wymondham NR18 9DF, UK; Plant Genetics Department, Niab, Park Farm, Cambridge CB24 9NZ, UK; Plant Genetics Department, Niab, Park Farm, Cambridge CB24 9NZ, UK; Plant Sciences Building, Sutton Bonnington Campus, University of Nottingham, Nottingham LE12 5RD, UK

**Keywords:** root hairs, plant phenotyping, machine learning, computer vision, AI, U-Net, wheat, roots, software

## Abstract

**Background:**

Root hairs play a key role in plant nutrient and water uptake. Historically, root hair traits have largely been quantified manually. As such, this process has been laborious and low-throughput. However, given their importance for plant health and development, high-throughput quantification of root hair morphology could help underpin rapid advances in the genetic understanding of these traits. With recent increases in the accessibility and availability of artificial intelligence (AI) and machine learning techniques, the development of tools to automate plant phenotyping processes has been greatly accelerated.

**Results:**

We present pyRootHair, a high-throughput, AI-powered software application to automate root hair trait extraction from microscope images of plant roots grown on agar plates. pyRootHair is capable of batch processing over 600 images per hour without manual input from the end user. In this study, we deploy pyRootHair on a panel of 24 diverse wheat (*Triticum aestivum* and *Triticum turgidum* ssp. *durum*) cultivars and uncover a large, previously unresolved amount of variation in many root hair traits. We show that the overall root hair profile falls under 2 distinct shape categories and that different root hair traits often correlate with each other. We also demonstrate that pyRootHair can be deployed on a range of plant species, including oat (*Avena sativa*), rice (*Oryza sativa*), teff (*Eragrostis tef*), and tomato (*Solanum lycopersicum*).

**Conclusions:**

The application of pyRootHair enables users to rapidly screen a large number of plant germplasm resources for variation in root hair morphology, supporting high-resolution measurements and high-throughput data analysis. This facilitates downstream investigation of the impacts of root hair genetic control and morphological variation on plant performance. pyRootHair is installable via PyPI (https://pypi.org/project/pyRootHair/) and can be accessed on GitHub at https://github.com/iantsang779/pyRootHair.

## Background

Root hairs are single-cell projections that can develop on all root surfaces [1]. They play an important role in facilitating nutrient and water uptake in plants [2, 3] and also help to maintain root–soil cohesion [4] and plant anchoring to soil [5]. The ability of root hairs to project outwards against high soil pressure has led researchers to use root hair cells (trichoblasts) as a model system to study plant responses to mechanical resistance [6]. Despite their importance, the morphological variation and genetic control of root hairs remain relatively underexplored compared to other root traits [7]. This is largely due to the inherent challenges of phenotyping root hairs, which are small in size and typically obscured by soil.

Most traditional root hair phenotyping techniques are relatively straightforward. Roots are typically grown on transparent agar plates and imaged after a few days, and the root hair measurements are manually recorded from microscope images. While this method works for small-scale studies, manual measurement of root hair traits is extremely time-consuming, becomes prohibitively expensive in larger studies, and is often prone to user bias. Studies often select low numbers of root hairs to measure length at predefined distances from the root tip [8, 9], and measurements are typically performed using the software FIJI [10–13]. Other methods involve selecting fully elongated root hairs from the mature zone of the root [14–16]. While this method is less time-consuming, it fails to provide spatial information on root hair length relative to the root tip and constrains measurements to a specific zone of the root. Furthermore, the manual selection of the ‘longest’ root hairs per image introduces a large amount of user bias, both in terms of selection and measurement. Since the acquired image is a 2-dimensional (2D) projection of a 3-dimensional (3D) object, some ‘long’ root hairs may appear longer or shorter in the image than in reality. An alternative growth method is the ‘cigar roll’ technique [15, 17], whereby seedling roots are grown on moist germination paper, and the paper is subsequently rolled into a cigar shape for subsequent plant growth. While possibly faster to set up than growing on agar, this method may inflict damage to the delicate root hairs upon unrolling of the ‘cigar’ prior to imaging.

For root hair density measurements, studies often perform manual counting of individual root hairs in a defined root zone [9, 13]. These methods are very laborious and time-consuming, and they are not suitable across all plant species. While the root hairs of the model dicotyledonous species *Arabidopsis thaliana* are relatively sparse and individually identifiable [18], other species, such as the cereal crop bread wheat (*Triticum aestivum*) [19] or the nitrogen fixing model species *Medicago truncatula* [20], have denser root hairs, meaning manual counting is simply not feasible. This principle also applies to manually selecting root hairs to measure, where in species with dense root hairs, determining the start and end of individual root hairs is challenging and often impossible.

More complex methods for root hair phenotyping have also been described. For example, X-ray computed tomography has been used to model phosphate uptake in wheat root hairs [21] and nutrient movement in the rhizosphere of rice (*Oryza sativa*) [22], providing extremely detailed characterization of root hairs in soil. Scanning and confocal microscopy techniques have also been deployed, in combination with microfludic platforms, to visualize root hairs with minimal disruption. These techniques have been used to quantify and study trichoblast nuclei at the cellular level [23–26]. While these techniques provide a vast amount of data at a high resolution, the equipment required is expensive, and the methods are both computationally and labour-intensive, making them unsuitable for medium- to large-scale screens of root hair morphology.

While a number of semi-automated methods for root hair phenotyping have been described, these remain limited in the throughput that can be achieved. In 2017, a semi-automated image analysis program was developed to quantify root hair density (RHD) from images of plants grown in *in situ* systems (e.g., rhizotrons—glass-fronted, soil-filled chambers that allow root observation over time), which required users to manually trace outlines of their images for training [27]. Similar *in situ* software to segment root hairs from plant roots grown in mini-rhizotrons was also developed, using a convolutional neural network (CNN) to perform image segmentation, enabling subsequent extraction of root hair length, diameter, and area [28]. RootHairSizer, a moderate-throughput (42 images per hour) algorithm for semi-automated measurements of root hair length, growth rate, and the differentiation zone from agar-grown images of roots, was developed in ImageJ [20]. In RootHairSizer, users are required to manually define the following: the bounding regions along the root for measurement, the thresholding method, and the measurement resolution. Recently, DIRT/$\mu$ was developed [29]—a Python-based software that utilizes machine learning to disentangle and measure individual root hairs, regardless of root hair density in the input image. However, this software is computationally expensive and potentially unsuitable for large-scale screening experiments.

Despite the advances in root hair phenotyping tools summarized above, their inability to automatically process large numbers of images with minimal manual intervention makes them potentially ill-suited to large-scale screening of root hair traits. Here, we present pyRootHair, a novel and fast software application designed for high-throughput root hair phenotyping. Primarily, pyRootHair uses a CNN for rapid and accurate image segmentation with a graphical processing unit (GPU), while also providing a simple random forest classifier (RFC) pipeline for an alternative segmentation method. For each individual image, pyRootHair extracts up to 15 summary traits and provides high spatial-temporal resolution of root hair length and area. To demonstrate its utility, we used pyRootHair to uncover significant varietal variation in all root hair traits measured across a panel of diverse wheat cultivars and define multiple uniquely identifiable traits, including ‘root hair profile’. We further demonstrate the effectiveness of pyRootHair in processing root hair images by exhibiting its use across a range of plant species and validate the automatically extracted traits relative to manual measurements. pyRootHair resolves a previously persistent bottleneck in root hair phenotyping. Its ability to uncover a wealth of previously undiscovered root hair phenotypic diversity across different plant species and populations will enable future genetic and physiological studies aimed at further understanding key aspects of the ‘hidden half’ of the plant.

## Data Description

The root hair dataset used consisted of 252 wheat seedling root images. Seeds for the 24 wheat cultivars (listed in Table 1), plus seed of 1 accession each of rice, tomato, teff, and oat, were surface sterilized and germinated on agar plates for 5 days. All root images were captured via light microscopy (Leica S9D) at 0.6$\times$ magnification and processed using pyRootHair.

**Table 1:** Name, ploidy, common name, and country of origin of all the wheat cultivars used in this study. Country of origin denoted by the IBAN Alpha-3 country code. DEU: Germany; DNK: Denmark; FRA: France; GBR: Great Britain; NLD: Netherlands; SWE: Sweden, USA: United States.

Cultivar	Ploidy	Species (common name)	Origin
Alchemy	Hexaploid	*Triticum aestivum* (bread wheat)	GBR
Banco	Hexaploid	*Triticum aestivum* (bread wheat)	SWE
Bersee	Hexaploid	*Triticum aestivum* (bread wheat)	FRA/GBR
Brigadier	Hexaploid	*Triticum aestivum* (bread wheat)	GBR
Brompton	Hexaploid	*Triticum aestivum* (bread wheat)	GBR
Claire	Hexaploid	*Triticum aestivum* (bread wheat)	GBR
Copain	Hexaploid	*Triticum aestivum* (bread wheat)	FRA
Cordiale	Hexaploid	*Triticum aestivum* (bread wheat)	GBR
Dakter	Tetraploid	*Triticum turgidum* ssp. *durum* (pasta wheat)	FRA
Flamingo	Hexaploid	*Triticum aestivum* (bread wheat)	DNK/NLD
Gladiator	Hexaploid	*Triticum aestivum* (bread wheat)	GBR
Hereward	Hexaploid	*Triticum aestivum* (bread wheat)	GBR
Holdfast	Hexaploid	*Triticum aestivum* (bread wheat)	GBR
Kloka	Hexaploid	*Triticum aestivum* (bread wheat)	DEU/DNK/GBR
Kofa	Tetraploid	*Triticum turgidum* ssp. *durum* (pasta wheat)	USA
Maris Fundin	Hexaploid	*Triticum aestivum* (bread wheat)	GBR
Rialto	Hexaploid	*Triticum aestivum* (bread wheat)	GBR
Robigus	Hexaploid	*Triticum aestivum* (bread wheat)	GBR
Slejpner	Hexaploid	*Triticum aestivum* (bread wheat)	DNK/SWE
Soissons	Hexaploid	*Triticum aestivum* (bread wheat)	FRA
Spark	Hexaploid	*Triticum aestivum* (bread wheat)	GBR
Steadfast	Hexaploid	*Triticum aestivum* (bread wheat)	GBR
Stetson	Hexaploid	*Triticum aestivum* (bread wheat)	GBR
Xi19	Hexaploid	*Triticum aestivum* (bread wheat)	GBR

## Analyses

### Variation in root hair–related traits

#### Root hair traits

**Figure 1:**
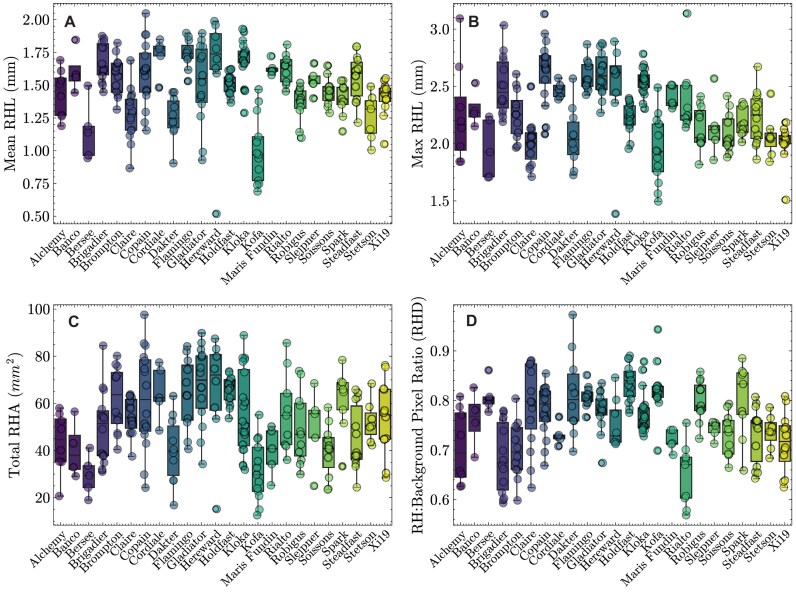
Root hair traits. Boxplots illustrate spread of data for each cultivar and each trait. The horizontal lines within each boxplot illustrate the median, whiskers represent the data range, and data from an individual image are displayed as a dot.

**Figure 2:**
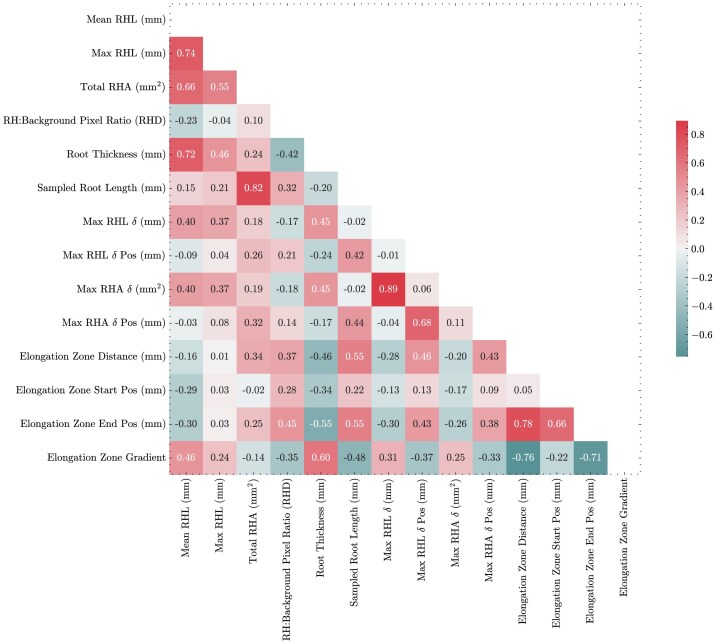
Correlation matrix illustrating relationships between root hair traits measured across the 24 wheat cultivars using pyRootHair. Numbers within squares illustrate Pearson correlation coefficients.

When using pyRootHair to extract root hair phenotypic data from images across a panel of 24 wheat cultivars, a large amount of variation was observed for all extracted root hair traits extracted (Fig. 1). Mean root hair length (RHL) varied between 1 and 2 mm for most cultivars. Within the bread wheat cultivars, Bersee had notably shorter root hairs relative to all other accessions (Fig. 1A). The durum wheat (*Triticum turgidum* ssp. *durum*) varieties Dakter and Kofa had shorter mean RHL than most bread wheat genotypes, potentially indicating the D genome in hexaploid wheat may contribute more to RHL relative to the A and B genomes of tetraploid wheat. Mean RHL exhibited a strong positive correlation with maximum RHL (Fig. 2, $R^{2}$ = 0.74, Fig. 1B). Total RHA (Fig. 1C) was positively correlated with mean RHL (Fig. 2, $R^{2} = 0.66$). This was unsurprising, as RHA was simply measured as the 2D area occupied by the root hair mask. As such, images that contained a longer section of root would have a higher mean RHL due to the inclusion of more root hairs in the mature zone of the root and thus a higher RHA. The root hair (RH)/background pixel ratio served as a proxy for RHD (Fig. 1D, Supplementary Fig. S1B). Interestingly, no correlation was observed between total RHA and RHD (Fig. 2, $R^{2} = 0.1$). This indicates that cultivars with a low RH/background pixel ratio (a proxy for high RHD, e.g., Rialto) may have high root hair density along the 3D root cylinder, but when projected onto a 2D plane (e.g., in an image), the increased root hair density does not result in increased root hair surface area (RHA). Since the RHD proxy is measured from pixel intensity, variation in image lighting quality would also affect this measurement, potentially resulting in mismatched correlations with total RHA.

#### Root traits

**Figure 3:**
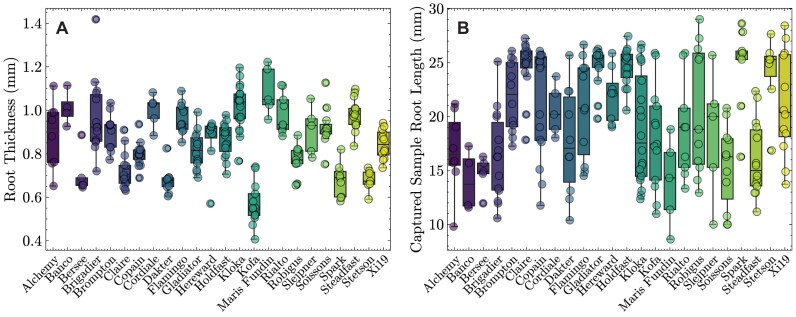
Root traits. Boxplots illustrate spread of data for each cultivar and each trait. The horizontal lines within each boxplot illustrate the median, whiskers represent the data range, and data from an individual image are displayed as a dot. RHA: root hair area; RHD: root hair density; RHL: root hair length.

The thickness of the captured root (i.e., mean width of the root excluding root hairs) (Fig. 3A) varied across all cultivars. Root thickness exhibited a strong positive correlation with mean RHL (Fig. 2, $R^{2} = 0.72$) and elongation zone gradient (Fig. 2, $R^{2} = 0.60$), and it was negatively correlated with the elongation zone end position (Fig. 2, $R^{2} = -0.55$). In addition to having a low mean RHL, the tetraploid cultivars Dakter and Kofa also displayed very narrow roots (Fig. 3A). Root length variation between cultivars and individuals was also observed (Fig. 3B). This was likely due to underlying genetic differences, as well as plate and positional growth room effects. As such, each image captured was positioned to contain the maximum length of root suitable for downstream processing. Thus, for cultivars with a consistent level of root growth (e.g., Gladiator, Spark, Stetson), there was minimal variation in captured sample root length. Conversely, cultivars with a high degree of variation in root length (e.g., Brigadier, Copain, Kloka) displayed large variation in captured sample root length (Fig. 3B).

#### Root hair heterogeneity traits

**Figure 4:**
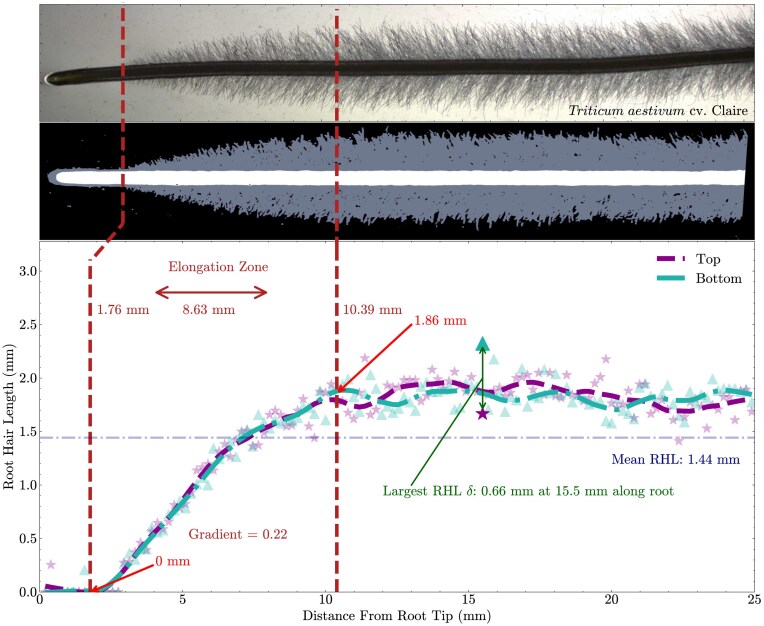
Visual representation of selected root hair traits extracted from an example bread wheat (cv. Claire) image using pyRootHair. (Top) Raw image. (Middle) Segmentation mask of the raw image. (Bottom) Green and purple markers illustrate root hair lengths along the root. Length profile for each segment (top or bottom) is illustrated by dashed regression lines through the markers. Vertical dashed maroon lines indicate the automatically identified root hair elongation zone, calculated as the largest region of continuous upward trajectory in the root hair profile. Gradient of the elongation zone is calculated as the maximum change in root hair length within the zone, divided by the length of the zone. Mean root hair length is calculated across the entire root length, illustrated by the dashed horizontal light purple line. The maximum difference ($\delta$) in root hair length between the root hair segments is 0.66 mm at 15.5 mm away from the root tip.

**Figure 5:**
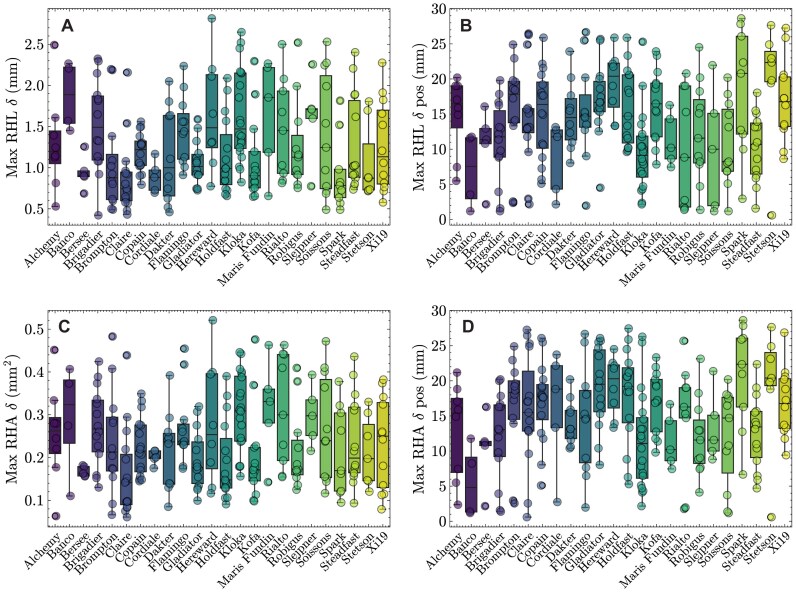
Root hair heterogeneity traits. Boxplots illustrate spread of data for each cultivar and each trait. The horizontal lines within each boxplot illustrate the median, whiskers represent the data range, and data from an individual image are displayed as a dot. $\delta$: difference.

Here, the trait ‘root hair heterogeneity’ was determined as the variation in root hair length and area within an individual root image (Fig. 4). Bread wheat cultivars, including Brigadier, Brompton, Hereward, and Soissons, all exhibited large fluctuations in max RHL $\delta$ between the left and right root hair segments (Fig. 5A). The position along the root of maximum RHL fluctuation also varied between cultivars (Fig. 5B), but no linear relationship with max RHL $\delta$ was found. In contrast, some cultivars (notably Robigus, Spark, and Steadfast) exhibited minimal variation in RHL $\delta$ between individuals, indicating these cultivars had more uniform root hair growth. Max RHA $\delta$ (Fig. 5C) and max RHA $\delta$ pos (Fig. 5D) measured the amount of RHA difference between the left and right root hair segments of an image and the corresponding position of this difference, respectively. Max RHL $\delta$ was strongly correlated with max RHA $\delta$ (Fig. 2). Since the root hair heterogeneity traits exhibited here are measuring absolute difference in length and area instead of taking a mean value of an image, these traits are very sensitive to the accuracy of the segmented image and should thus be used with caution.

#### Elongation zone traits

**Figure 6:**
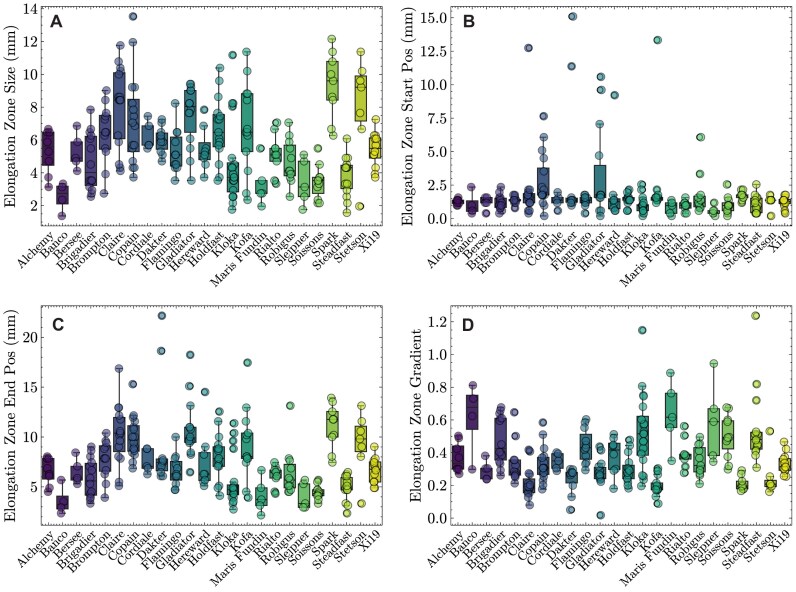
Root hair elongation zone traits. Boxplots illustrate spread of data for each cultivar and each trait. The horizontal lines within each boxplot illustrate the median, whiskers represent the data range, and data from an individual image are displayed as a dot.

The elongation zone was defined here as the largest continuous region where root hair length increases (Fig. 4, region bound by the vertical dashed maroon lines). Notably, the bread wheat cultivar Spark had a very large elongation zone size (Fig. 6A). Three cultivars (Claire, Copain, and Kloka) all exhibited wide variation in elongation zone size between biological replicates. The starting position of the elongation zone was relatively consistent between all cultivars, in close proximity to the root tip (Fig. 6B). In contrast, the end position of the elongation zone varied more relative to the start position (Fig. 6C), as highlighted by the high correlation between the elongation zone distance and end position (Fig. 2, $R^{2} = 0.78$). The gradient of the elongation zone (Fig. 6D) was negatively correlated with the stop position (Fig. 2, $R^{2} = -0.71$) and distance (Fig. 2, $R^{2} = -0.76$).

**Figure 7:**
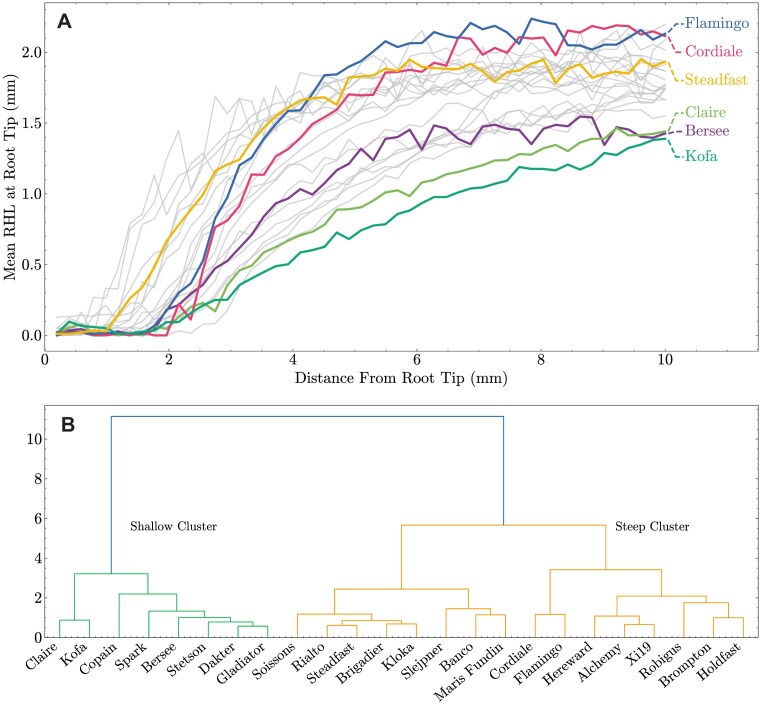
Variation in root hair profile around the root tip (1 cm) across the 24 wheat cultivars phenotyped using pyRootHair. (A) Mean root hair length (RHL) profile, highlighting cultivars exhibiting extreme variation in RHL within the region displayed. (B) Unsupervised agglomerative clustering of root hair profiles highlights 2 main clusters of root hair profiles around the root tip: ‘Shallow’ (green cluster) and ‘Steep’ (orange cluster). See Supplementary Fig. S1A for examples of cultivars with ‘shallow’ and ‘steep’ profiles.

The greatest variation in overall root hair profile was found around the root tip, where root hairs had begun to emerge. As such, this diversity in root hair morphology around the root tip may likely play an important role in affecting nutrient uptake. A large degree of variation was found for both root hair emergence (minimum distance from root tip where hairs begin emerging) and overall root hair profile across the 1-cm section from the root tip (Fig. 7A). Unsupervised clustering of the root hair profiles revealed 2 major clusters, termed here ‘shallow’ and ‘steep’ (Fig. 7B). Cultivars in the ‘shallow’ cluster (including Kofa, Bersee, and Claire) had a shallower root hair profile gradient (Fig. 6D, 7A, Supplementary Fig. S1A). In contrast, cultivars in the ‘steep’ cluster (including Flamingo, Cordiale, and Steadfast) had a steeper root hair gradient and generally had longer root hairs around the root tip compared to cultivars in the ‘Shallow’ cluster (Fig. 7A, Supplementary Fig. S1A). Of particular note, Cordiale, Steadfast, and Claire all originated from the same country (GBR, Table 1) and exhibited vastly different root hair profiles (Cordiale and Steadfast: steep, Claire: shallow) (Fig. 7A). As such, this indicated that root hair morphology may not be strongly influenced by soil type and climate, but rather more by genetic control.

### Adaptability

**Figure 8:**
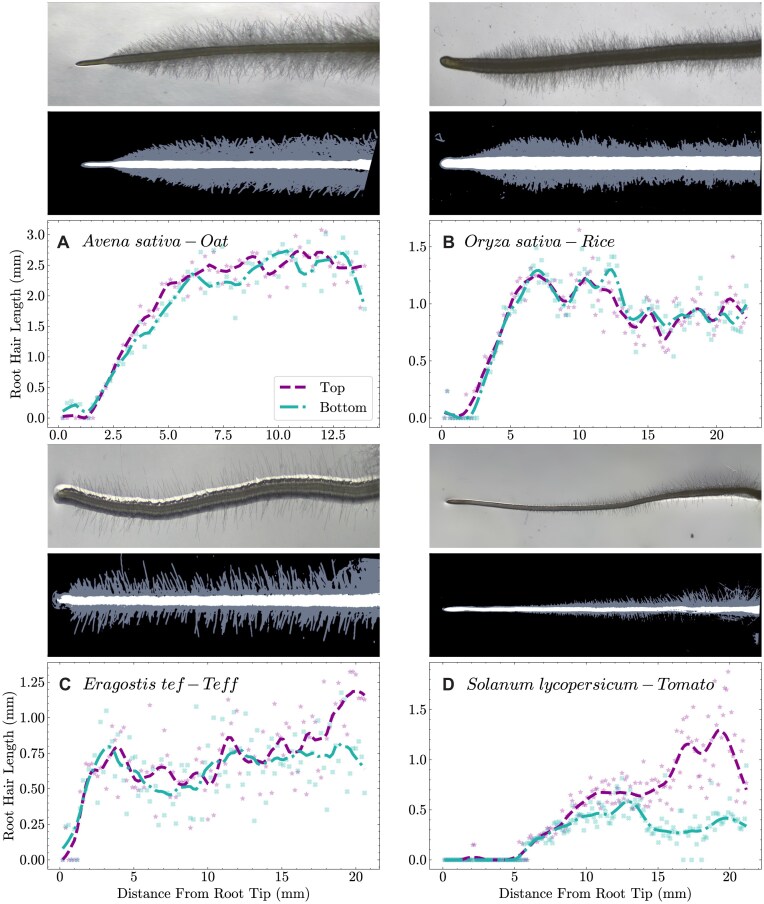
pyRootHair deployed on root images captured from different plant species: (A) oat, (B) rice, (C) teff, and (D) tomato. For each panel: raw images on top, predicted segmentation masks in the middle, and automated plots of root hair length (RHL) on the bottom. Raw images and segmentation masks not to scale. Top = top segment of root hairs; bottom = bottom segment of root hairs.

Given that overall root morphology is relatively consistent throughout many higher plant species, the methodology deployed in the pyRootHair workflow can be translated to a wide range of wild and cultivated plant species. Here, we demonstrated that pyRootHair can accurately segment and quantify root hair traits by detailed investigation of wheat, as well as further investigation in single accessions from 4 other species: oat, rice, teff, and tomato (Fig. 8, Supplementary Fig. S2).

### Trait validation

To validate traits extracted by pyRootHair, the RHL, elongation zone length, and root length were manually measured across different wheat images. Manual RHL measurements were taken from 5 different images, with measurements performed at 0.1-mm intervals along the root across both root hair segments. Manual measurements of the elongation zone and root length were taken from 54 and 68 different images, respectively. Validation of RHL measurements in prestraightened (prior to affine transformation, Supplementary Fig. S9A) and straightened (after affine transformation, Supplementary Fig. S9C) roots were performed on 15 images. All manual measurements were calibrated using the conversion factor of 102 pixels per 1 mm. Additional validation of RHL measurements was performed in oat, rice, teff, and tomato ($R^{2} = 0.91$, Supplementary Fig. S2).

**Figure 9:**
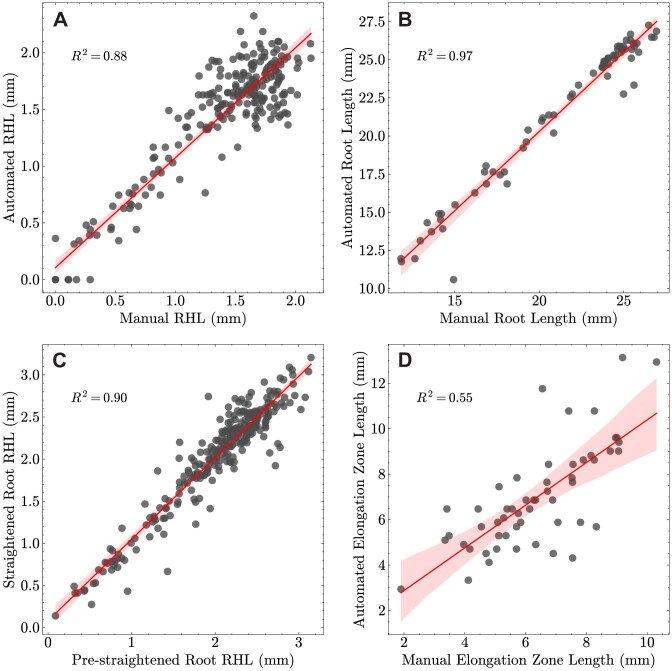
Correlation ($R^{2}$) between manually measured traits and automated trait extraction using pyRootHair. (A) root hair length (RHL). (B) Root length. (C) RHL in prestraightened and straightened images. (D) Elongation zone length. Regression line with 99% confidence interval illustrated in red.

Strong positive correlations between automated and manual measurements were found for RHL along the root (Fig. 9A, $R^{2} = 0.88$) and root length (Fig. 9B, $R^{2} = 0.97$). RHL measurements from prestraightened and straightened roots exhibited a strong correlation (Fig. 9C, $R^{2} = 0.9$). The CNN model exhibited accurate segmentation of input images compared to manually annotated segmentation masks, with intersection over union (IoU) scores >0.8 across different example images (Supplementary Fig. S4). Automated measurements of the elongation zone length correlated less well with manual measurements (Fig. 9D, $R^{2} = 0.55$). While traits such as RHL and root length were simple to manually measure, estimating the elongation zone length was significantly more subjective due to small fluctuations in the root hair profile. The method of elongation zone measurement deployed in pyRootHair selects the largest continuous region of constant root hair growth, as determined by the gradient of the root hair profile (Supplementary Fig. S3). This approach of utilizing the gradient to select the elongation zone is more robust than visual quantification. However, this approach does not discriminate against biological anomalies, such as roots with bald patches near the root tip, and may flag a region far from the root tip as the elongation zone. As such, end-user validation of output data is critical.

### Performance

Across all images for the 24 wheat cultivars, the mean per-image processing time (including inference) was 7.82 seconds with an Nvidia L40S GPU with 8 GB of video random access memory (VRAM), compared to 7.97 seconds per image using the RFC pipeline (Supplementary Fig. S5A) on a compute node with 20 GB RAM. The mean processing time per image was 430 seconds when using the CNN to perform inference without a GPU (Supplementary Fig. S5A), which was significantly slower than either the GPU or RFC pipelines (Supplementary Fig. S5B). No significant difference in mean image processing time was found between the 2 pipeline configurations. Since all input wheat images were obtained from the same imaging setup, the image quality and lighting remained consistent. As such, for this comparison, an RFC model was trained on a single input image and used to perform inference on all subsequent images. Naturally, the simple RFC model produced less refined segmentation masks of input images compared to the CNN deployed in the main pipeline. Despite the reduction in segmentation accuracy with the RFC model, the output data of the random forest pipeline displayed a relatively linear relationship with the output data generated from the main pipeline using a GPU (Supplementary Fig. S5C, D).

## Discussion

Advances in computer vision and artificial intelligence (AI) have greatly accelerated the field of plant phenotyping. Here, we present pyRootHair, a rapid and new computer vision-based software application for high-throughput extraction of root hair traits from root images. To our knowledge, pyRootHair extracts more traits per input image than any other available software. It has a low per-image processing time and enables large-scale batch processing of images. Importantly, no user input is required during computation, which eliminates user measurement bias. Furthermore, unlike previous methods [28], pyRootHair is readily available, simple to install and operate, and entirely open source. As such, pyRootHair significantly improves the accessibility, speed, and accuracy of root hair phenotyping. The throughput achievable with pyRootHair means that the bottleneck in acquiring root hair data has transitioned from the phenotyping step to the plant growth and image acquisition process.

The limitations of pyRootHair primarily relate to the curvature of root hairs and the segmentation performance. While straightening of the input root is effective at standardising the measurement along each root hair segment, RHL is calculated as the width of the root hair segmentation mask and does not account for the inherent curvature within the individual root hairs. While DIRT/$\mu$ accurately resolves the length of individual root hairs at the cost of computational throughput [29], pyRootHair quantifies overall root hair morphology of an image at high speeds with lower hardware demand, while sacrificing some individual root hair resolution. Our approach offers increased throughput, and the fully automated nature of the package avoids any user selection biases towards longer root hairs. Secondly, accuracy and effectiveness of trait extraction are dependent on the segmentation performance. While the CNN deployed by the main pipeline is flexible and powerful, training instances are laborious to annotate and cannot be generated to cover all examples of end-user images. The RFC pipeline is computationally inexpensive but far less flexible compared to the main pipeline with the CNN.

The variation in root hair morphology identified across the 24 wheat cultivars investigated here highlights useful entry points for future genetic and physiological investigation of root hair morphology on plant performance. In particular, traits such as the shape of the root hair profile and heterogeneity have, to our knowledge, not been previously quantified. Identification and formalization of these new traits increases the likelihood of identifying genetic loci and genes controlling root hair morphology via forward genetic analyses and for their physiological investigation (e.g., via genome-wide association studies). For example, given that root hair length is positively correlated with phosphorous uptake in different crops [11, 30], automated RHL calculation via pyRootHair serves as a convenient and fast method of prescreening a panel of cultivars for field trials or breeder selection. Future work should investigate the impact of the traits outlined in this study on plant physiology. Examples may include quantifying whether root thickness affects root foraging or whether elevated RHA and RHD can promote increased nutrient/water uptake across different soil conditions. Future experiments based on data gathered via pyRootHair have the potential to significantly advance our understanding of how root hair morphology affects crop physiology.

## Potential Implications

Due to the throughput achieved and ease of use, we believe that pyRootHair can significantly reduce the bottleneck in root hair phenotyping and serve as a key tool for uncovering the genetic loci and genes controlling root hair morphology in crops and other plant species. Through this process, candidate genes controlling root hair traits can be identified and selectively bred into existing populations to develop more resilient, nutrient-efficient crops for the future.

## Methods

### Plant material and image acquisition

All plant material is summarized in Table 1. The 24 wheat cultivars were sourced from field-grown trials in Cambridgeshire. The accessions included 22 bread wheat cultivars, encompassing all founder lines within the ‘Niab Elite MAGIC’ (Multi-Parent Advanced Generation Inter-Cross) and ‘Niab Diverse MAGIC’ populations [31, 32]. Two durum wheat (*T. turgidum* ssp. *durum*) varieties (Dakter, Kofa) were also included. Seeds for oat, rice, teff, and tomato were either sourced from locally available seed stocks at Niab or commercially purchased. Medicago, brachypodium, and arabidopsis images were provided by [20].

Cold-treated seeds (stored for 5 days at 4$^{\circ }$C) were surface sterilized with 70% ethanol for 60 seconds, then immersed in 20% bleach (sodium hypochlorite) for 10 minutes. Seeds were then washed 3 times with sterile distilled water in a laminar flow hood and plated onto square agar plates (Sigma-Aldrich, 120 × 120 × 17 mm) with sterile forceps. Four seeds were placed on each plate, with each seed positioned in a corner with the embryos oriented towards the centre of the plate. Each agar plate contained 50 mL of media prepared with of 0.5 g sucrose (Sigma-Aldrich), 0.15 g phytagel (Sigma-Aldrich), and 0.2 g Murashige and Skoog basal salt (Sigma-Aldrich), mixed with 50 mL dH$_{2}$O. Plates were sealed with micropore tape (3M) and placed horizontally on flat racks in a well-lit, 25$^{\circ }$C growth room for 5 days under constant lighting. Subsequently, the roots were imaged using a Leica S9D Stereomicroscope at 0.6$\times$ magnification, 5 days after germination.

### Segmentation model

To generate and train the segmentation model, we used the self-configuring CNN generator nnU-Netv2 [33]. Segmentation masks of training instances were manually curated using the interactive segmentation tool ilastik [34]. For each segmentation mask, pixels associated with the background were labelled with 0, root hairs as 1, and roots as 2. Training instances varied in input dimensions, clarity, and lighting conditions. The training set was composed of 44 wheat, 5 arabidopsis, 14 medicago, 3 brachypodium, 4 teff, 4 maize (*Zea mays*), and 9 rice images, along with the corresponding segmentation masks.

Model training was carried out on the crop diversity high-performance computer (HPC) cluster [35] using an NVIDIA A100 SXM4 80 GB Tensor Core GPU. The ‘nnUNetResEncUNetMPlans’ planner preset was used for all nnU-Net stages [36]. Training was conducted with the ‘all’ fold and the ‘2d’ configuration arguments. Image augmentation was automatically performed by the nnU-Net pipeline. Z-score normalization was carried out for the 3 input image channels (red, green, blue). The model was trained for 1,000 epochs with a batch size of 13. The overall best validation Dice score of the model was 98% (Supplementary Fig. S6). For root and root hair segmentation masks, mean validation Dice scores were 0.97 and 0.99, respectively, across all training instances, while mean IoU scores were 0.84 and 0.97 respectively (Supplementary Fig. S7).

### Pipeline configurations

To accommodate a variety of end-user hardware, pyRootHair was designed to operate across different levels of computing power, including HPC systems and personal computers. As such, different pipeline configurations are available for the end user.

By default, the main pipeline utilizes a GPU to perform inference on input images. GPU requirements vary depending on input image sizes. For reference, an Nvidia L40S GPU with 8 GB VRAM was sufficient to perform inference on input images of size 2,600 × 1,500 × 3. Inference without a GPU is still possible on a CPU, but speed will be significantly affected.

For users without a GPU, pyRootHair offers a simple alternative pipeline. Users can easily train an RFC segmentation model on a representative image of their choice with a single command. The trained RFC model can be subsequently used to run inference on input images. Alternatively, end users can generate their own segmentation masks, which can then be individually processed for trait extraction without a GPU.

### Workflow

**Figure 10:**
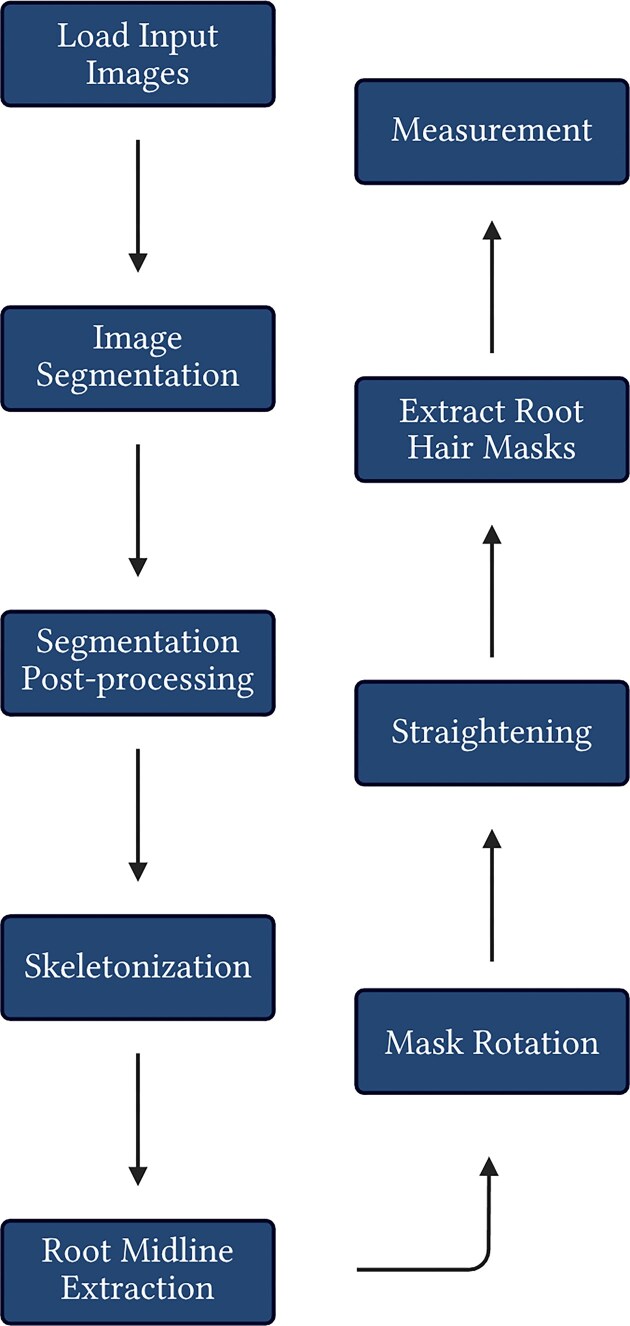
Simple overview of pyRootHair processing methodology.

For a given batch of input images with the main or RFC segmentation pipeline, the predicted masks are generated first. The postprocessing steps of each segmentation mask obey the following methodology for trait extraction:

For each mask, the root is first extracted and segmentation noise removed (Fig. 10, Supplementary Fig. S8A–C). The root is then skeletonised to a single-pixel-wide representation of the root (Fig. 10). A spline (smooth curve fitted through a set of points) is subsequently mapped to the skeleton coordinates, and the root midline is approximated via computing the median of each bin in a sliding window down the root mask (Fig. 10, Supplementary Fig. S8D).

To standardise trait extraction, roots must be oriented downwards in the input image. The orientation of the root in the segmentation mask is calculated from the approximated root midline and rotated such that the root tip points downwards (Fig. 10, Supplementary Fig. S9A). The rotated root mask is re-skeletonised and coordinates are remapped to approximate the midline of the rotated root (Fig. 10). Next, to minimize the effect of any inherent root curvature within the rotated root, the entire segmentation mask is straightened via piecewise affine transformation [37], producing a straightened segmentation mask of the original image (Fig. 10, Supplementary Fig. S9B, C).

After straightening the mask, the root tip is located using kernel convolution along the skeleton of the straightened root (Supplementary Fig. S9C), and the root hair masks are extracted on either side of the root (Fig. 10, Supplementary Fig. S9D).

To extract RHL, the longest RHL is calculated in each bin from a sliding window along each root hair segment, starting at the root tip. The size of the window can be controlled via the –resolution argument. The default value of 20 pixels per bin size was used to compute traits from all images analysed in this article. RHA is calculated as the total pixel area of root hair mask within each bin. The root hair elongation zone is defined as the region where root hairs experience continuous positive growth. The ‘profile’ of the root hair segment is modelled using a locally weighted scatterplot smoothing (LOWESS) regression line, and the elongation zone position, size, and gradient are extracted from the longest region of the root where the gradient of the root hair profile continuously increases (Fig. 4, Supplementary Fig. S3B). The heterogeneity of root hair growth is defined as the difference in RHL and RHA between the ‘left’ and ‘right’ sides of the root hair segment. The greatest difference in RHL and RHA between each root hair segment is recorded as ‘max RHL/RHA $\delta$’, along with the corresponding position along the root (Fig. 4). All traits are translated from pixel measurements to millimetre measurements, where the pixel/millimetre conversion factor can be controlled via the –conv argument. A full list of extracted traits is available in Table 2.

**Table 2:** Summary traits calculated for each input image. RHA: root hair area; RHL: root hair length.

Trait	Explanation
Name	Name of image file
Batch ID	Batch ID specified via -b/–batch-id
Mean RHL (mm)	Mean root hair length of the input image, calculated across the entire length of the root
Max RHL (mm)	Maximum recorded root hair length in the input image
Min RHL (mm)	Minimum recorded root hair length in the input image
Total RHA (mm$^{2}$)	Total root hair area of the input image
Max RHL Delta (mm)	Largest difference in root hair length between each root hair segment on either side of the root
Max RHL Delta Pos (mm)	Position along the root corresponding to the largest difference in root hair length
Max RHA Delta (mm)	Largest difference in root hair area between each root hair segment on either side of the root
Max RHA Delta Pos (mm)	Position along the root corresponding to the largest difference in root hair area
Elongation Zone Distance (mm)	Length of the elongation zone, defined as the largest continuous region where root hairs are emerging
Elongation Zone Start (mm)	The position along the root where the elongation zone starts, calculated as distance from the root tip
Elongation Zone End (mm)	The position along the root where the elongation zone ends, calculated as distance from the root tip
Elongation Zone Gradient	The gradient of the elongation zone, calculated as $\delta$ RHL in elongation zone/length of elongation zone
Root Thickness (mm)	Mean thickness of the root, calculated via a sliding window down the segmented root mask
Captured Sample Root Length (mm)	Total length of the straightened root captured within the input image
RH Pixel Intensity Mean	Mean pixel intensity of the area occupied by the root hairs in the input image
Background Pixel Intensity Mean	Mean pixel intensity of the background in the input image
RH/Background Pixel Ratio	RH pixel intensity divided by background pixel intensity

RHA was calculated as the total area occupied by the root hairs in each segmentation mask. RHD was estimated using the RH/background pixel ratio (Fig. 1D). This ratio was calculated as the mean pixel intensity of the root hairs in the input image, divided by the mean pixel intensity of the background. In backlit images, the lighter background would have a higher mean pixel intensity, while the root hairs would be darker due to their obstruction of light and thus have a lower mean pixel intensity. If a cultivar had more root hairs per unit area of root, the root hairs would be darker, decreasing the pixel intensity value of the root hairs. Thus, assuming consistent lighting across input images, lower RH/background ratios could indicate a cultivar had a higher RHD, while higher RH/background ratios could indicate a cultivar had a lower RHD (Supplementary Fig. S1B). Furthermore, the variation in mean background pixel intensity within each cultivar illustrates the consistency of lighting conditions between images and can therefore serve as a quality control metric.

### Output

For a given batch of input images, pyRootHair produces a summary table and a raw table in comma-separated value format. The summary table contains the 15 traits listed in Table 2 and is calculated for each image in the input image folder. The raw table contains the individual RHL and RHA measurements from each bin in the sliding window for each input image.

Users can quickly view the summary information displaying the RHL and RHA profile for each image via the –plot-summary flag. To visualize the segmentation and transformation of the input image, –plot-segmentation saves the generated segmentation mask of each image. To view how the straightening was performed, –plot-transformation saves a graphical representation of the root warping. To operate pyRootHair, users only need to provide 3 required arguments: -i/–input specifies the filepath/directory to the input image folder. -b/–batch-id specifies the subfolder name associated with the current run, which is stored in the output directory. -o/–output specifies a filepath/directory to store the output data and plots.

Since varying root length in the input image affects how summary traits (e.g., mean RHL, total RHA) are calculated, we offer the argument –length-cutoff, which allows users to specify a length cutoff for input roots (measured in millimetres from the root tip). –length-cutoff standardises trait calculation for all images in the input batch based on the value provided. For example, –length-cutoff 10 will return summary and raw tables of only the first 10 mm of the root for all images in the batch. This mitigates a significant portion of bias introduced with varying root length, enabling standardisation of traits for more detailed analysis.

### Dependencies

pyRootHair was written in the Python programming language (v3.12.7) and developed on the crop diversity HPC, running the Debian 12 Bookworm operating system [35]. Scikit-image (v0.24.0) was used to carry out most image processing functionalities. Numerical calculations were carried out using the numpy (v2.0.2) and scipy (v1.14.1) libraries. Data tables were constructed using pandas (v2.2.3), and all plots and figures were created using matplotlib (v3.9.3). LOWESS regression lines were computed using statsmodels (v0.14.4). Scikit-learn (v.1.5.2) was used for quality control of segmented images. nnU-Netv2 (v2.5.1) was used to create the image segmentation model with PyTorch (v.2.5.1) and CUDA (v.12.6).

## Availability of Source Code and Requirements

- Project name: pyRootHair
- Project homepage: https://github.com/iantsang779/pyRootHair
- Operating system(s): Linux, MacOS, Windows
- Programming language: Python
- License: MIT License

## Supplementary Material

giaf141_Supplemental_File

giaf141_Authors_Response_To_Reviewer_Comments_Original_Submission

giaf141_GIGA-D-25-00279_Original_Submission

giaf141_GIGA-D-25-00279_Revision_1

giaf141_Reviewer_1_Report_Original_Submission Wanneng Yang -- 8/10/2025

giaf141_Reviewer_1_Report_Revision_1 Wanneng Yang -- 10/20/2025

giaf141_Reviewer_2_Report_Original_Submission Nicolas Gaggion -- 8/20/2025

giaf141_Reviewer_2_Report_Revision_1 Nicolas Gaggion -- 10/27/2025

giaf141_Reviewer_3_Report_Original_Submission Peter Pietrzyk -- 9/7/2025

giaf141_Reviewer_3_Report_Revision_1 Peter Pietrzyk -- 11/3/2025

## Data Availability

All additional supporting data are available in the *GigaScience* repository, GigaDB [38]. The source jupyter notebook and data used to generate all figures in the manuscript have been deposited on GitHub [39]. Dome-ML annotations can be found in the DOME Registry [40].

## Additional Files

**Supplementary Fig. S1**. Highlighting variation in root hairs around root tips. (A) Representative images of root tips of wheat cultivars with ‘shallow’ (Bersee, Claire) and ‘steep’ (Flamingo, Steadfast) root hair profiles, as identified in Fig. 7. Red lines illustrate the difference in gradient of the root hair profile at root tip. (B) A visual representation between cultivars with a higher (Holdfast) and lower (Kloka) RH/background pixel ratio. Low values indicate increased root hair area (RHA), while high values indicate low RHA.

**Supplementary Fig. S2**. Validation of root hair length (RHL) measurements in oat, rice, teff, and tomato. Manual RHL measurements were performed in FIJI across 3 different images for all 4 species. Regression line with 99% confidence interval illustrated in red.

**Supplementary Fig. S3**. Automated root hair elongation zone calculation. (A) Example segmentation mask of a root hair section. (B) Example of a plot automatically generated by pyRootHair with –plot-summary, corresponding to data extracted from the mask in (A). Red crosses illustrate root hair length (RHL) from sliding window along root hair mask. Purple line illustrates mean root hair profile derived from a regression line. Dashed green line illustrates the gradient of the root hair profile (purple line). Regions where *y* > 0 for the dashed green line indicate regions of positive root hair growth, highlighted by the grey rectangles. The largest region is automatically determined as the root hair elongation zone, bound by the vertical dashed black lines. RH: root hair.

**Supplementary Fig. S4**. Validation of convolutional neural network (CNN) segmentation on 4 selected images. (A–D) Raw images. (E–H) Manually annotated segmentation masks of the above images using ilastik [34]. (I–L) Predicted segmentation masks generated from the CNN. Intersection over union (IoU) scores for the root hair masks are shown at the bottom. IoU scores illustrate proportion of overlapping pixels in the manually annotated (E–H, grey) and the predicted (I–L, grey) root hair masks, divided by the total area of root hair masks minus the overlapping region (intersection).

**Supplementary Fig. S5**. Comparing processing times between different pyRootHair pipeline configurations. (A) Boxplot illustrating the mean processing duration in seconds (including inference) for each image within each cultivar input folder. Mean runtime per image annotated above/below each box. CPU pipeline utilized the same CNN deployed in the main (GPU) pipeline to perform inference without a GPU. (B) Cumulative runtime for all 252 images used in this study across all 3 pipeline options. (C) Summary data and (D) mean normalized summary data calculated for all images using the default pipeline (GPU) and the random forest classifier (RFC) pipeline. The GPU pipeline was run using an Nvidia L40S GPU with 8 GB VRAM. The RFC pipeline was run on an HPC compute node with 20 GB RAM using an RFC model trained on a single image. CNN: convolutional neural network; CPU: central processing unit; GPU: graphical processing unit; HPC: high-performance computer; RFC: random forest classifier.

**Supplementary Fig. S6**. nnUNet model training metrics. Illustration of training loss, validation loss, and Dice scores for each epoch (*n*= 1,000). Mean validation Dice score: 0.98 across 83 training instances.

**Supplementary Fig. S7**. nnUNet model validation metrics. (A) Dice and IoU scores for root and root hair masks for each individual training image (*n* = 83) during model validation. Confusion matrices for (B) root and (C) root hair segmentation masks during validation. Values within each cell represent the mean number of pixels across all training images assigned to the particular class. FN: false negative; FP: false positive; IoU: intersection over union; TN: true negative; TP: true positive.

**Supplementary Fig. S8**. Segmentation, root extraction, and midline approximation. (A) Example of a raw input image into pyRootHair. (B) Segmentation mask of the input image. (C) Segmentation mask of all root objects. (D) Removal of nonprimary root objects within the mask. The root mask is skeletonised, and a cubic spline is mapped to the root skeleton. A sliding window is used to approximate the root midline (in red) by calculating the median coordinates of the cubic spline down the root. The root endpoints (in parentheses) are retrieved from the approximated midline and used to calculate the angle ($\theta$) of the root relative to the vertical (*y*-axis). Here, the angle $\theta$ is calculated by $\arctan \left( {\frac{|2665-146|}{|97 - 1348)|}} \right)^\circ = 63.6^{\circ }$.

**Supplementary Fig. S9**. Root straightening. (A) Segmentation mask of input image, rotated by angle $\theta$ such that the root is oriented downwards. (B) Root midline is approximated as previously described (Supplementary Fig. S8), illustrated by green crosses. Orange stars illustrate midline of new, straight root, which starts and ends at the same endpoints of the original root (red circles). Euclidean distances between midline points are preserved from the original root midline to the new root midline. The direction of image transformation is shown by the pink arrow. (C) Straightened segmentation mask via piecewise affine transformation from $scikit-image$ [37] and the root tip is located (purple circle). (D) Removal of root mask, preserving the 2 root hair segments on either side of the root. Root hair masks are cropped such that an equal length of root hair segment is processed downstream.

## Abbreviations

AI: artificial intelligence; CNN: convolutional neural network; GPU: graphical processing unit; HPC: high-performance computer; IoU: intersection over union; LOWESS: locally weighted scatterplot smoothing; RFC: random forest classifier; RH: root hair; RHA: root hair area; RHD: root hair density; RHL: root hair length; VRAM: video random access memory.
