# Supplementary material for: pyRootHair: Machine learning accelerated software for high-throughput phenotyping of plant root hair traits
Source: Gigascience. 2025 Nov 13;15:giaf141. doi: 10.1093/gigascience/giaf141 (PMC12824728; doi:10.1093/gigascience/giaf141)
Supplement: giaf141_Supplemental_File [file giaf141_supplemental_file.pdf]

433 ## 13 Supplementary Figures

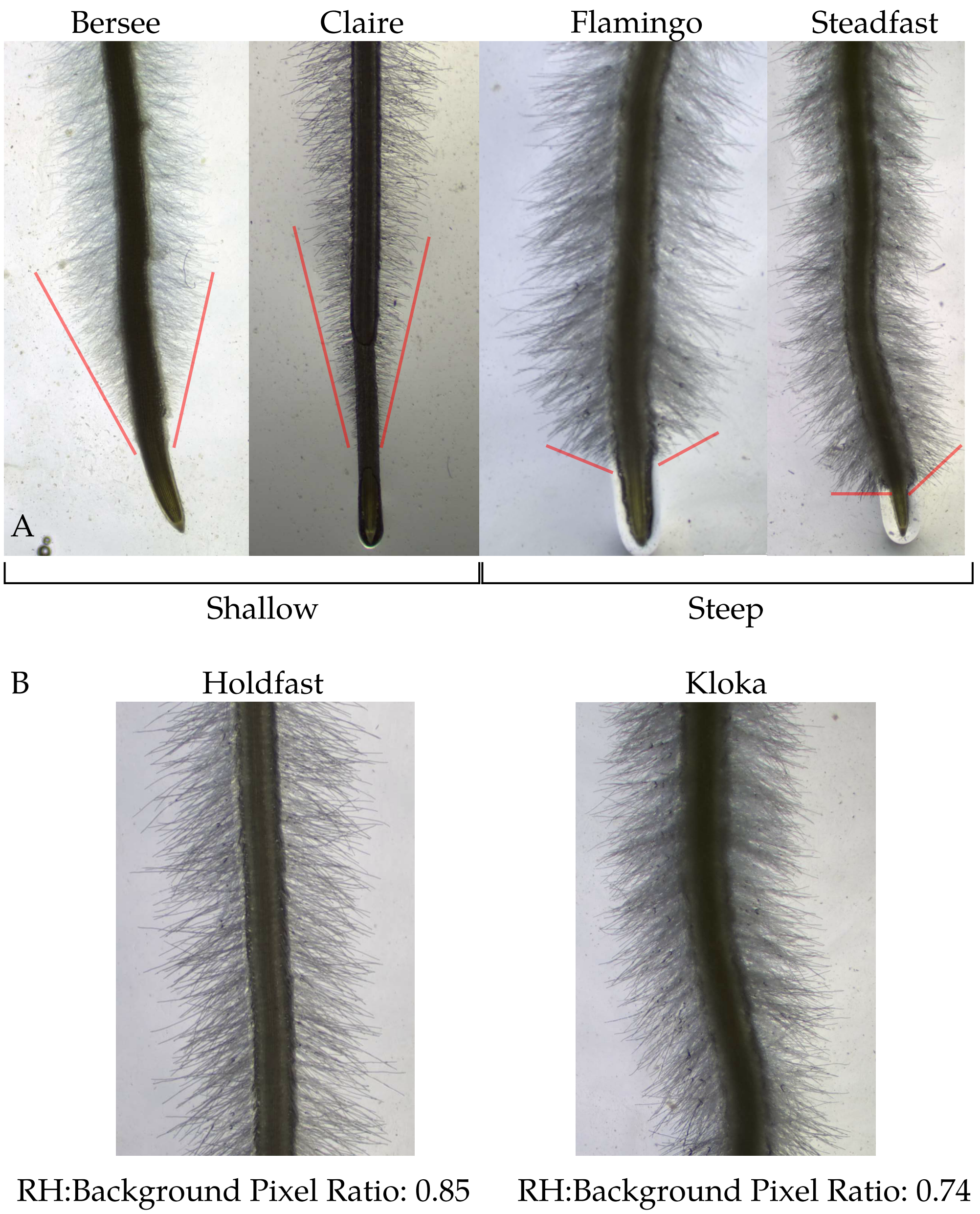

Supplementary Figure 1: **Highlighting variation in root hairs around root tips.** A) Representative images of root tips of wheat cultivars with 'shallow' (Bersee, Claire) and 'steep' (Flamingo, Steadfast) root hair profiles as identified in Figure 7. Red lines illustrate the difference in gradient of the root hair profile at root tip. B) A visual representation between cultivars with a higher (Holdfast) and lower (Kloka) RH:Background Pixel Ratio. Low values indicate increased root hair area (RHA), while high values indicate low RHA.

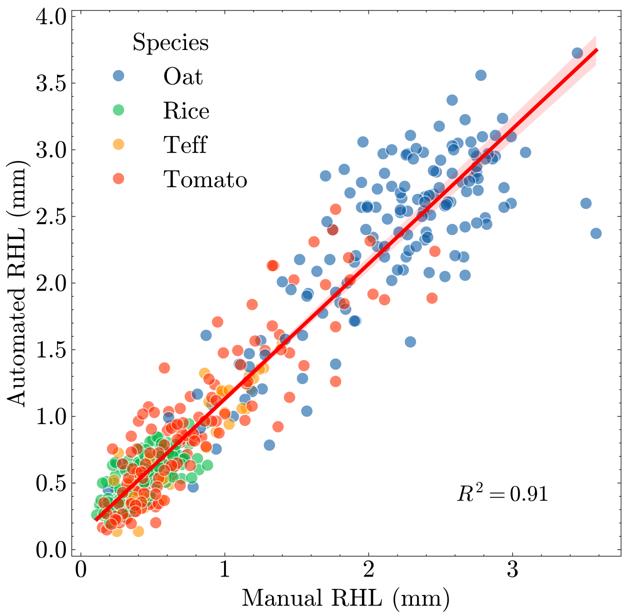

Supplementary Figure 2: **Validation of root hair length (RHL) measurements in oat, rice, teff and tomato.** Manual RHL measurements were performed in FIJI across three different images for all four species. Regression line with 99% confidence interval illustrated in red.

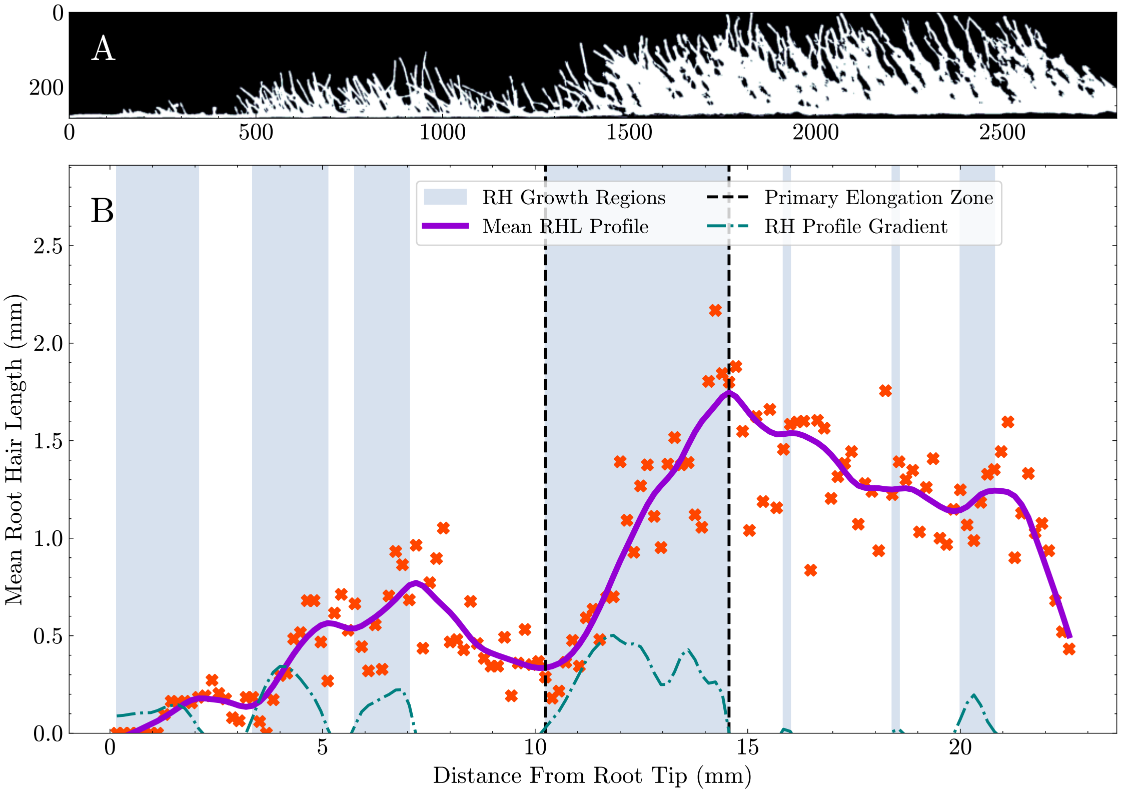

Supplementary Figure 3: **Automated root hair elongation zone calculation.** A) Example segmentation mask of a root hair section. B) Example of a plot automatically generated by pyRootHair with --plot-summary, corresponding to data extracted from the mask in A). Red crosses illustrate root hair length (RHL) from sliding window along root hair mask. Purple line illustrates mean root hair profile derived from a regression line. Dashed green line illustrates the gradient of the root hair profile (purple line). Regions where $y>0$ for the dashed green line indicate regions of positive root hair growth, highlighted by the grey rectangles. The largest region is automatically determined as the root hair elongation zone, bound by the vertical dashed black lines. RH: Root Hair

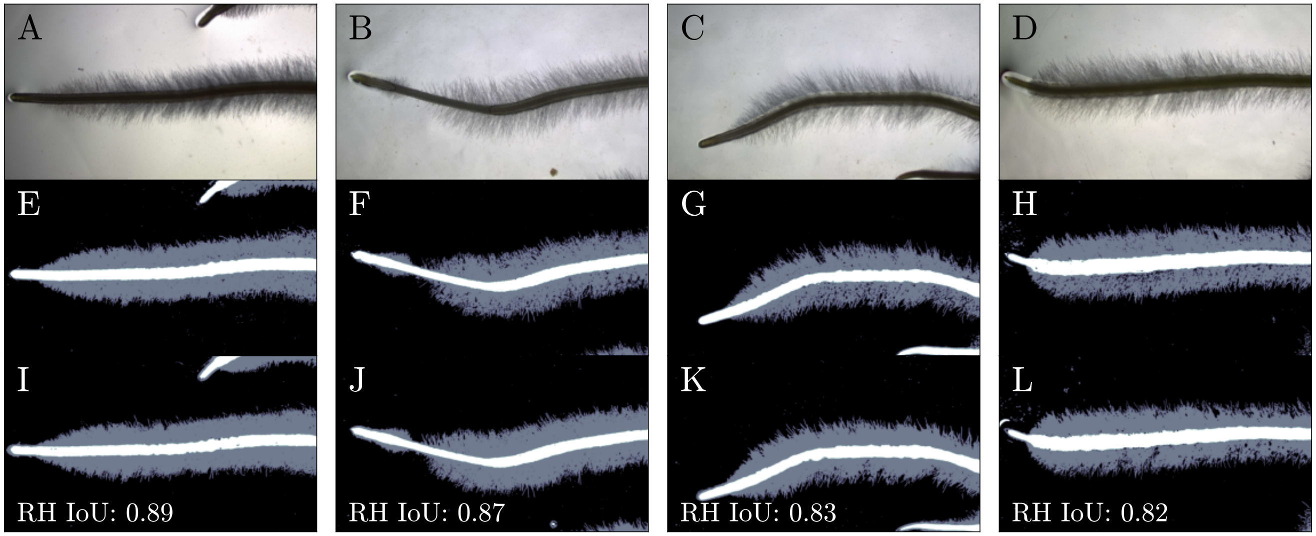

Supplementary Figure 4: **Validation of Convolutional Neural Network (CNN) segmentation on four selected images**. A-D) Raw Images, E-H) Manually annotated segmentation masks of the above images using ilastik (Berg et al. 2019), I-L) Predicted segmentation masks generated from the CNN. Intersection over union (IoU) scores for the root hair masks are shown at the bottom. IoU scores illustrate proportion of overlapping pixels in the manually annotated (E-H, grey) and the predicted root hair masks (I-L, grey), divided by the total area of root hair masks minus the overlapping region (intersection).

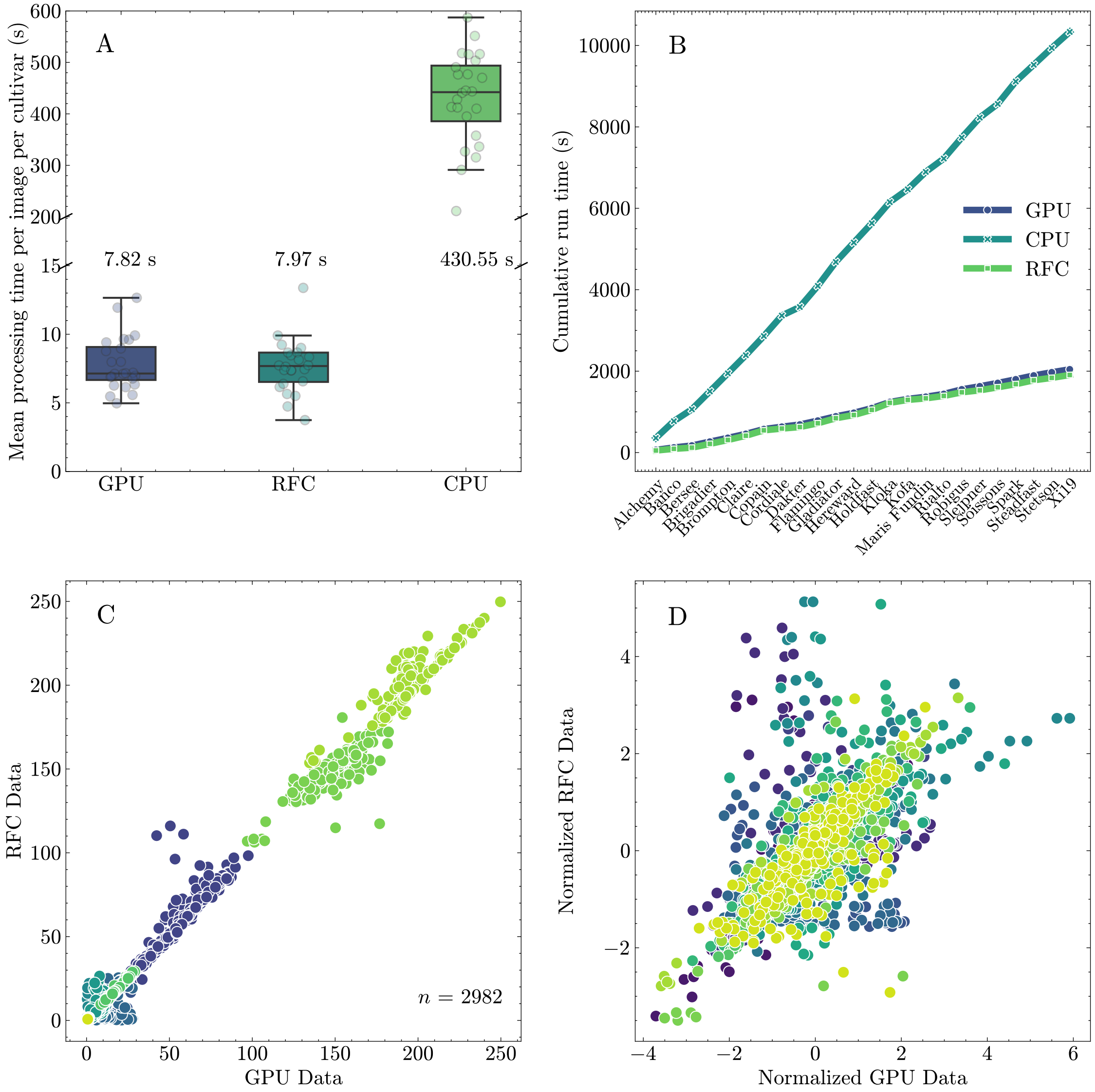

Supplementary Figure 5: **Comparing processing times between different pyRootHair pipeline configurations.** A) Boxplot illustrating the mean processing duration in seconds (including inference) for each image within each cultivar input folder. Mean runtime per image annotated above/below each box. CPU pipeline utilized the same CNN deployed in the main (GPU) pipeline to perform inference without a GPU. B) Cumulative run time for all 252 images used in this study across all three pipeline options. (C) Summary data and (D) mean normalized summary data calculated for all images using the default pipeline (GPU) and the random forest classifier (RFC) pipeline. The GPU pipeline was run using an Nvidia L40S GPU with 8 GB VRAM. The RFC pipeline was run on an HPC compute node with 20 GB RAM using a RFC model trained on a single image. CNN: convolutional neural network. GPU: Graphical Processing Unit. RFC: Random Forest Classifier. CPU: Central Processing Unit, HPC: High Performance Computer

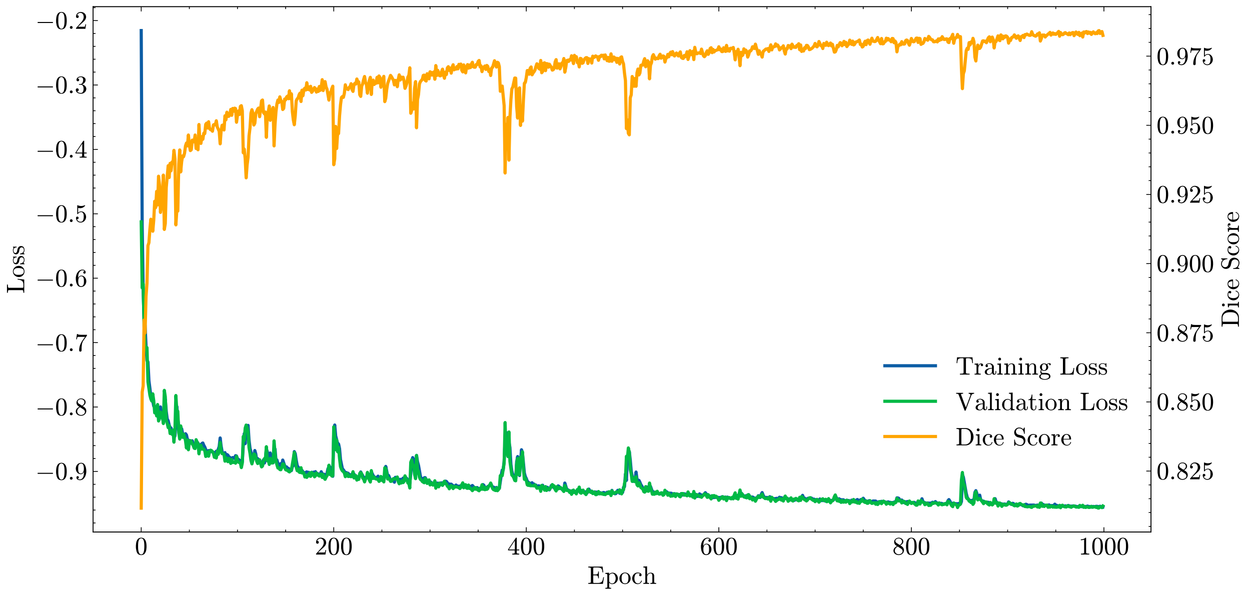

Supplementary Figure 6: **nnUNet Model Training Metrics.** Illustration of training loss, validation loss and dice scores for each epoch (n=1000). Mean validation dice score: 0.98 across 83 training instances.

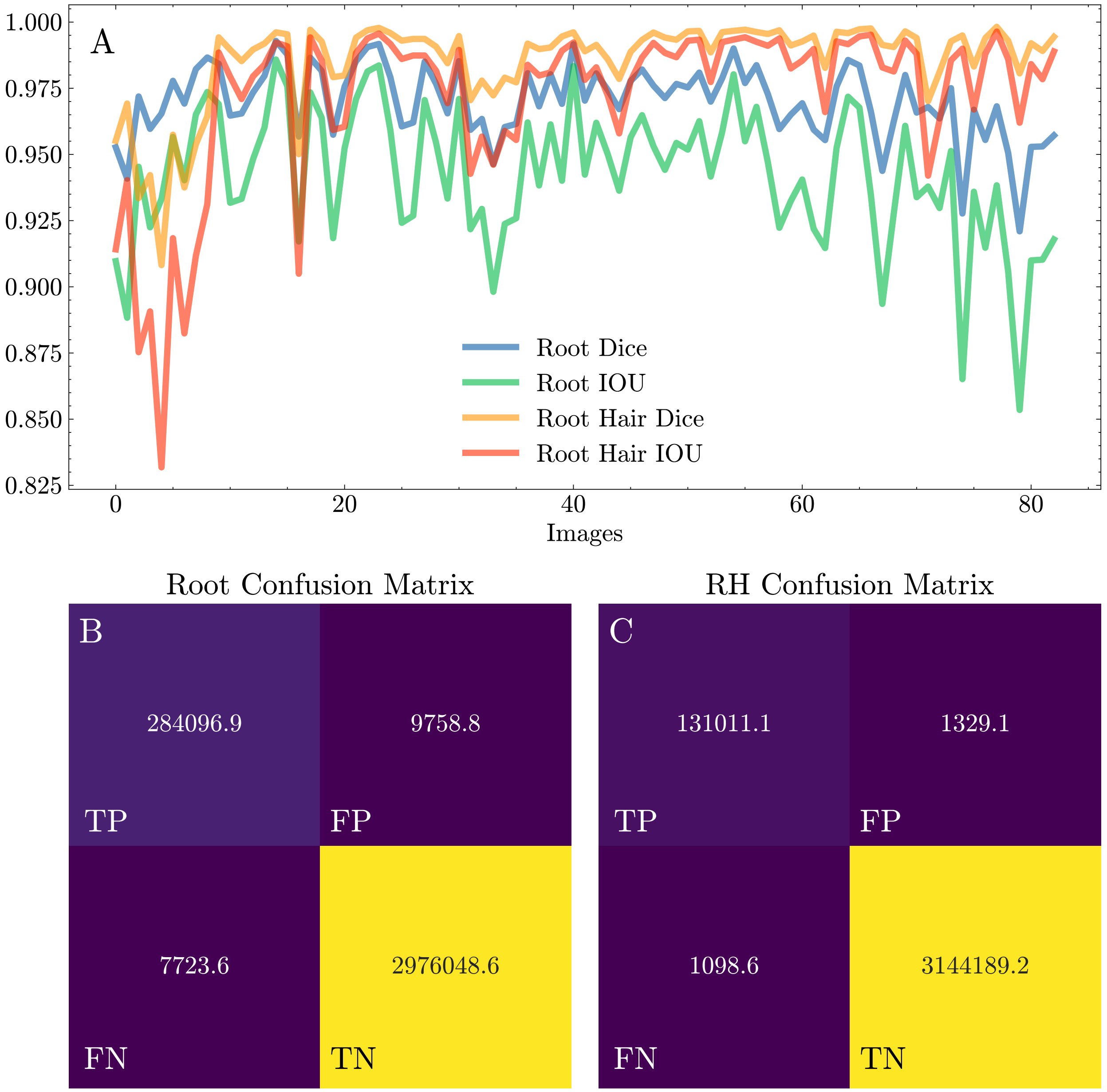

Supplementary Figure 7: **nnUNet Model Validation Metrics.** A) Dice and IoU scores for root and root hair masks for each individual training image ($n = 83$) during model validation. Confusion matrices for B) root and C) root hair segmentation masks during validation. Values within each cell represent the mean number of pixels across all training images assigned to the particular class. TP: True Positive, TN: True Negative, FP: False Positive, FN: False Negative, IoU: Intersection Over Union

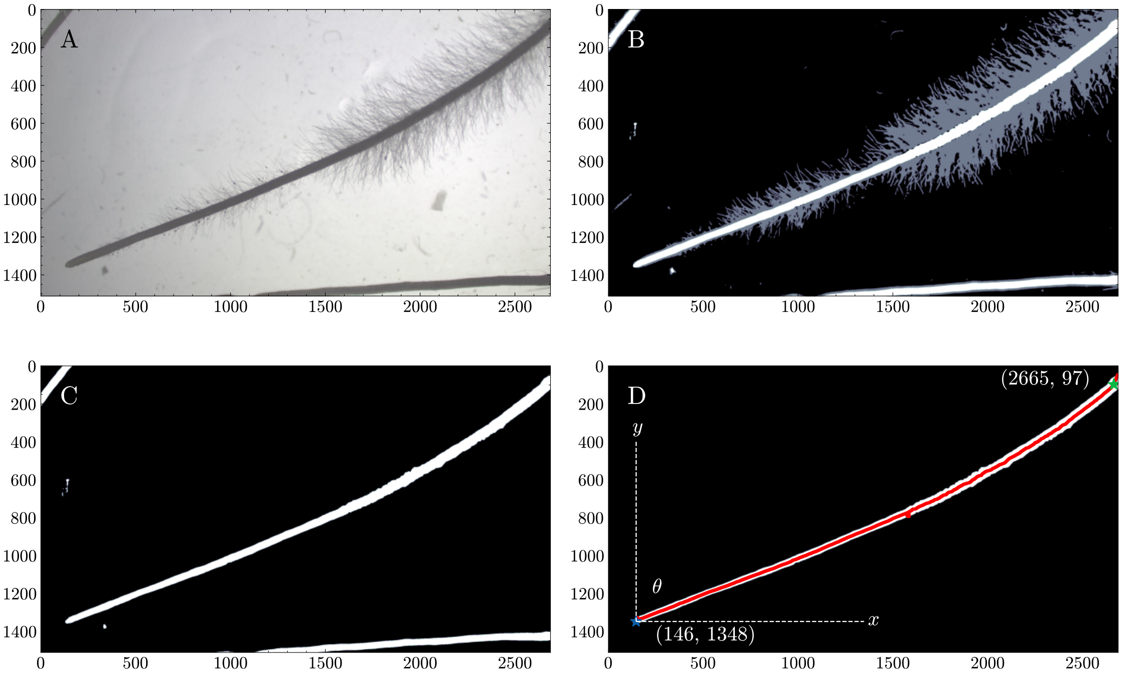

Supplementary Figure 8: **Segmentation, root extraction and midline approximation** A) Example of a raw input image into pyRootHair. B) Segmentation mask of the input image. C) Segmentation mask of all root objects. D) Removal of non-primary root objects within the mask. The root mask is skeletonized, and a cubic spline is mapped to the root skeleton. A sliding window is used to approximate the root midline (in red) by calculating the median co-ordinates of the cubic spline down the root. The root endpoints (in parentheses) are retrieved from the approximated midline and used to calculate the angle (θ) of the root relative to the vertical (y-axis). Here, the angle θ is calculated by $\arctan\left(\frac{|2665-146|}{|97-1348)|}\right)^{\circ} = 63.6^{\circ}$.

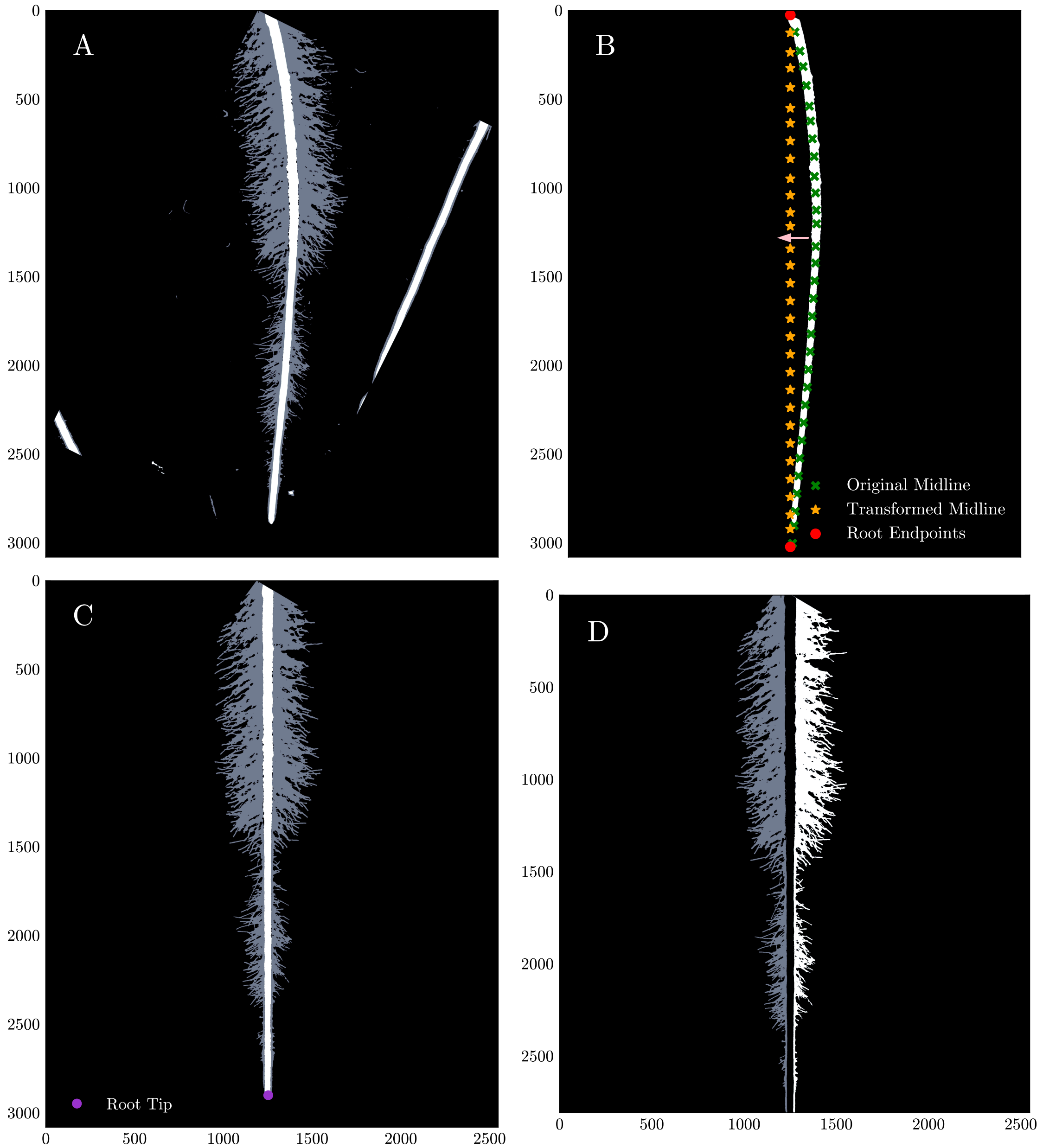

Supplementary Figure 9: **Root straightening** A) Segmentation mask of input image, rotated by angle θ such that the root is oriented downwards. B) Root midline is approximated as previously described (Supplementary Figure 8), illustrated by green crosses. Orange stars illustrate midline of new, straight root, which starts and ends at the same endpoints of the original root (red circles). Euclidean distances between midline points are preserved from the original root midline to the new root midline. The direction of image transformation is shown by the pink arrow. C) Straightened segmentation mask via piecewise affine transformation from $scikit - image$ (Van Der Walt et al. 2014) and the root tip is located (purple circle). D) Removal of root mask, preserving the two root hair segments on either side of the root. Root hair masks are cropped such that an equal length of root hair segment is processed downstream.
