# Supplementary material for: pyRootHair: Machine learning accelerated software for high-throughput phenotyping of plant root hair traits
Source: Gigascience. 2025 Nov 13;15:giaf141. doi: 10.1093/gigascience/giaf141 (PMC12824728; doi:10.1093/gigascience/giaf141)
Supplement: giaf141_GIGA-D-25-00279_Revision_1 [file giaf141_giga-d-25-00279_revision_1.pdf]

GigaScience

pyRootHair: Machine Learning Accelerated Software for High-Throughput Phenotyping of Plant Root Hair Traits

--Manuscript Draft--

**Manuscript Number:**	GIGA-D-25-00279R1
**Full Title:**	pyRootHair: Machine Learning Accelerated Software for High-Throughput Phenotyping of Plant Root Hair Traits
**Article Type:**	Research
**Funding Information:**	Biotechnology and Biological Sciences Research Council (BB/W009439/1) — Mr Ian Tsang, Dr Fiona Leigh<br>Biotechnology and Biological Sciences Research Council (BB/X018725/1) — Dr Lawrence Percival-Alwyn, Dr James Cockram
**Abstract:**	Root hairs play a key role in plant nutrient and water uptake. Historically, root hair traits have largely been quantified manually. As such, this process has been laborious and low-throughput. However, given their importance for plant health and development, high-throughput quantification of root hair morphology could help underpin rapid advances in the genetic understanding of these traits. With recent increases in the accessibility and availability of artificial intelligence (AI) and machine learning techniques, the development of tools to automate plant phenotyping processes has been greatly accelerated. Here, we present pyRootHair, a high-throughput, AI-powered software application to automate root hair trait extraction from microscope images of plant roots grown on agar plates. pyRootHair is capable of batch processing over 600 images per hour without manual input from the end user. In this study, we deploy pyRootHair on a panel of 24 diverse wheat (Triticum aestivum and Triticum turgidum ssp. durum) cultivars and uncover a large, previously unresolved amount of variation in many root hair traits. We show that the overall root hair profile falls under two distinct shape categories, and that different root hair traits often correlate with each other. We also demonstrate that pyRootHair can be deployed on a range of plant species, including oat (Avena sativa), rice (Oryza sativa), teff (Eragrostis tef) and tomato (Solanum lycopersicum). The application of pyRootHair enables users to rapidly screen a large number of plant germplasm resources for variation in root hair morphology, supporting high-resolution measurements and high-throughput data analysis. This facilitates downstream investigation of the impacts of root hair genetic control and morphological variaton on plant performance.
**Corresponding Author:**	Ian Tsang<br>NIAB: National Institute of Agricultural Botany<br>Cambridge, Cambridgeshire UNITED KINGDOM
**Corresponding Author Secondary Information:**	
**Corresponding Author's Institution:**	NIAB: National Institute of Agricultural Botany
**Corresponding Author's Secondary Institution:**	
**First Author:**	Ian Tsang
**First Author Secondary Information:**	
**Order of Authors:**	Ian Tsang<br>Lawrence Percival-Alwyn<br>Stephen Rawsthorne<br>James Cockram<br>Fiona Leigh<br>Jonathan Atkinson

Powered by Editorial Manager® and ProduXion Manager® from Aries Systems Corporation

**Order of Authors Secondary Information:**	
**Response to Reviewers:**	Dear Editor and Reviewers,<br><br>I would like to thank you all for providing helpful and constructive feedback, which I believe has significantly improved the quality of the manuscript. All suggestions and comments have been addressed, unless this was not possible, in which case I have provided my reasoning. I hope that these changes have suitably addressed the limitations of the manuscript, and that it is now suitable for publication in Gigascience.<br><br>---<br><br>Reviewer #1: This paper introduces an artificial intelligence-driven software named pyRootHair, which enables high-throughput automated extraction of root hair traits from plant root images, thereby facilitating rapid analysis of root hair morphological variations in various plants, including wheat. However, the following issues remain:<br><br>1) Compared to previously published work, the contributions and innovations of this study are not sufficiently highlighted. For instance, the work by Lu, Wei, Xiaochan Wang, and Wei Jia, titled "Root hair image processing based on deep learning and prior knowledge" (Comput. Electron. Agric. 202, 2022: 107397), should be explicitly referenced to clarify the advancements presented here.<br><br>Specifically, compared to Lu et al 2022, their method proposed a custom uNet model to segment root hair images. However, their model is not explicitly available from the paper for end users to download and operate, or use for direct benchmarking against pyRootHair. I have referenced Lu et al 2022 in the discussion, adding the fact that pRrootHair is easy to install and operate and available on GitHub (Lines 230-231)<br><br>2) Although the study demonstrates that pyRootHair can be applied to multiple plant species, including Arabidopsis, Brachypodium, rice, and tomato, the primary validation and analysis are conducted on wheat. For other species, only segmentation results and trait extraction figures are presented, lacking detailed comparative validation with manual measurements as thoroughly as for wheat.<br><br>I have included manual validation of RHL measurements in oat, rice, tomato and teff, as illustrated in S.Figure 2. This has been mentioned in text in lines 194-195.<br><br>3) The process of "straightening" curved roots is implemented, but the potential introduction of new errors by this procedure is not discussed.<br><br>I have included panel C in figure 9 (manual correlation plots), which shows a high correlation (R^2 = 0.9) between RHL measurements in pre-straightened roots and straightened roots. Since the images are rotated relative to the vertical first before affine transformation, the rotation ensures that for most images, only a small portion of the root is warped by the transformation. This has also been updated in text, line 198<br><br>4) In the trait validation section, the correlation analysis between automated and manual measurements shows strong agreement for root hair length and root length, but weaker correlation for elongation zone length. The study should provide a more in-depth discussion on the possible reasons for this lower correlation.<br><br>To summarize, the elongation zone trait is hard to visually quantify due to the fluctuating nature of the root hair profile. As such, the manual measurements differ from the automated calculation, which utilizes the gradient of the root hair profile to determine the elongation zone. This has been expanded on in text in lines 202-209, and a visual representation has been supplied in S. Figure 3.<br><br>5) The details of the core algorithms (CNN architecture, random forest classifier) are insufficiently described. Key aspects such as parameter selection, optimization, training procedures, and the division ratios of the training/validation/test sets are not clearly specified. Additionally, the specific strategies for data augmentation are not mentioned.

One of the advantages of using nnUNet (https://www.nature.com/articles/s41592-020-01008-z) to generate a segmentation model is that nnUNet automatically performs dataset splitting, optimization, cross validation and data augmentation as part of the integrated pipeline. Furthermore, nnUNet performance is state of the art and has been benchmarked in multiple segmentation challenges (e.g. AMOS22, MSD). As such, I would argue that it is not necessary to provide the details of model architecture, parameter selection, optimization and training, as the nnUNet pipeline is extremely robust and well documented. Nevertheless, metrics such as dice and IOU scores have been provided in text (lines 299-301), and training/validation loss curves and confusion matrices have been provided in S.Figures 6-7. Additional details of the model configuration can be found in the plans file on huggingface: https://huggingface.co/iantsang779/pyroothair_v1/blob/main/plans.json. . As for the Random Forest Classifier, the pyroothair_train_random_forest command is a simple wrapper around scikit-learn's RandomForestClassifier(), which only accepts a single segmentation mask, and the RGB image of the segmentation mask for training. The train_random_forest command is a wrapper around the skimage.future.TrainableSegmenter() class, where the skimage.feature.multiscale_basic_features() function is used to extract intensity, edges and texture properties from the image to train the RFC. No default RFC models are provided by pyroothair, as the RFC models are much less adaptable compared to the CNN trained by nnUNet, and should be used only for users with very minimal compute power available.

6) No quantitative comparisons with similar tools (e.g., in terms of speed and accuracy) are provided.

The DIRT/µ package was developed to process images of close-up sections of root hairs, where the root hairs are not expected to have any overlap with each other and images must be taken at a small focal depth (Pietrzyk et al., 2024). As such, the images used in this study are not compatible with DIRT/µ, vice versa. This was confirmed when I attempted to run DIRT \µ and was unable to extract any data for my images due to raised errors. When attempting to run RootHairSizer, I kept encountering an error in the source code; an error which persisted even when attempting to use the author's own images. As I am unfamiliar with Java/ImageJ Macros, I wasn't able to debug the issue. Other methods mentioned in the manuscript, such as those of Vincent et al 2017, are not suitable for agar-based images. Lu et al 2022's CNN model is not publicly accessible or deployable within a software package, and only serves to segment images taken in rhizotrons, and furthermore, does not provide a full package for analysis to generate data. As such, there no software currently available, to my knowledge, that generates data similar to those presented in this manuscript. To summarize, I believe that this highlights the novelty of pyRoothair, and demonstrates its ease of use, both in terms of computation, and imaging requirements.

Reviewer #2: The manuscript "pyRootHair: Machine Learning Accelerated Software for High-Throughput Phenotyping of Plant Root Hair Traits" presents a valuable tool for plant phenotyping in microscopy images. As it stands, my recommendation is to accept after minor revisions.

I found the GitHub repository provides detailed instructions and was straightforward to install and run. The whole process took only a few minutes to execute, which speaks well to the software's accessibility.

To further enhance the clarity, precision, and accessibility of the manuscript, I have several comments.

1.) On the random forest classifier training process, the manuscript states "For this comparison, the RFC was trained on a single input image, and used to perform inference on all subsequent images." However, the repository documentation indicates: "To train a random forest model, you will need to train the model on a single representative example of an image, and a corresponding binary mask of the image."

	The repository further notes that "You will need to ensure that all the images are relatively consistent in terms of lighting, appearance, root hair morphology, and have the same input dimensions. Should your images vary for these traits, you will need to train separate random forest models for different batches of images."<br><br>The manuscript needs clarification on this training process. Please specify whether users are expected to manually segment one of their own images, use the nnUNet model to generate binary segmentation and refine it using annotation tools (such as ilastik), or select one of the images from ones provided by the authors of the manuscript that best matches their new data.<br><br>If a user decides to use a random forest classifier to perform segmentation on their input images, they will need to:<br><br>1.) Choose one representative image of their choice as input to the RFC classifier<br><br>2.) Generate a segmentation mask of the selected image in 1), using ilastik<br><br>3.) Train a random forest classifier on the image from 1.) and the segmentation mask from 2.) using pyroothair_train_random_forest, which is just a wrapper around scikit-learn's RandomForestClassifier(). During training, the pyroothair_train_random_forest command extracts features from the image (intensity, edges and texture) via the skimage.feature.multiscale_basic_features() function.<br><br>4.) Perform inference on their images using the trained model from 3.). To deploy the RFC, users can run pyroothair_run_random_forest, specifying the path to the trained RFC model from 3.) with –rfc.<br><br>In line 218, I used a single image to train a RFC, to perform inference on all other wheat images. Since all the images were taken under the same microscope setup and conditions, the image quality was relatively consistent, and thus the RFC was able to acceptably segment the input images for the most part. However, if users have images of more varying quality, or images of different species, then it would be more appropriate to train different classifiers for different groups of input images. The RFC segmentation option has been designed to be quick and lightweight, offering a fraction of the segmentation power of the CNN, but still allowing users with minimal hardware available to perform some inference on their images. This has been clarified in the manuscript, lines 217-220.<br><br>2.) Regarding nnUNet performance, I support the decision not to compare with other models, as nnUNet represents state-of-the-art performance and enables easy training for non-expert users. However, I have several questions: Do you plan to release the training dataset so users can retrain the model by incorporating new manually annotated data? The manuscript would benefit from quantifying segmentation performance by crop type. Measuring performance solely by computing time is insufficient, and quantitative metrics such as Dice scores on test holdout sets or cross-validation results (as performed by the nnUNet model) should be reported.<br><br>All training images, and the corresponding segmentation masks, were uploaded to GigaDB upon initial submission, which should be publicly available if the manuscript is accepted. I have added the training and validation loss curves, as well as the dice scores per epoch to S.Figure 6. To reduce the segmentation model size, the final model was trained with the –f all option, as 5 fold CV did not drastically improve the already excellent segmentation performance on existing training images. I have reported validation dice and IoU scores for the overall data set, and for each image in section 8.2 in Line 300-301. In addition, I have added S.Figure 7, which illustrates the dice and IOU scores for the root and root hair segmentation masks in each image during validation, and the confusion matrices for root and root hair segments.<br><br>3.) The current abstract describes pyRootHair as an "AI-powered software application to automate root hair trait extraction from images of plant roots grown on agar plates." This description needs to clarify that images were obtained via microscopy. Do you have insights on how the trained model performs across different microscope systems?

Powered by Editorial Manager® and ProduXion Manager® from Aries Systems Corporation

The model performs well on different images gathered from different microscope systems. Provided that the images are sufficiently backlit and in focus, the model should have no problem producing an acceptable segmentation mask. The quality of the segmentation masks is more dependent on the imaging setup (i.e decent lighting, minimal foreign objects in frame, clear root view), rather than different microscope brands/setups. I generated numerous poor training images, with varying contrast, lighting conditions, and contamination/foreign objects in frame, so the model can adapt. I have amended the abstract on line 19 such that it now reads '... automate root hair trait extraction from microscopy images of plant roots...

4.) The manuscript requires additional clarification on the root straightening process using piecewise transformation, as this represents an important step in the measurement procedure. Please specify how this is performed and whether a specific algorithm or function from a library is used for the piecewise affine transformation. For readers who are not computer vision specialists, a figure illustrating the measurement steps (segmentation → skeletonization → straightening → measurement) would be valuable.

The straightening process is performed using the PiecewiseAffineTransform class from scikit-image. I have added Supplementary Figures 8 and 9, which provide a visual representation of most pipeline stages, including a representation of the affine transformation (S.Figure 9B,C), and additional explanation has been added in the Figure Caption. For further clarification, the original Figure 1, which provided a graphical representation of the pipeline, has been significantly simplified now in Figure 10, which should aid reader clarity, especially for those with non-technical backgrounds. Figure 9 (manual correlation plots) has been updated to include panel D, which shows a high correlation between manual RH measurements in pre-straightened and straightened roots.

5.) Really minor comments: It would be helpful if the demo generated all plots by default, and Random Forest Classifier (RFC) is not included in the abbreviation list.

The command pyroothair_run_demo will now automatically generate, and save segmentation masks and summary plots. The arguments –plot-segmentation, --plot-transformation and –plot-summary have been removed from pyroothair_run_demo. This change has also been reflected in the github documentation. RFC has been added to the abbreviation list.

Overall, this represents solid work that addresses an important need in plant phenotyping research. The suggested clarifications will enhance both the scientific rigor and practical utility of the contribution.

Reviewer #3: I really enjoyed reading this manuscript because it covers a small but very important niche in plant phenotyping. Root hairs play a crucial role in water and nutrient uptake, but phenotyping them remains a challenge. The manuscript introduces pyRootHair, an AI based image analysis tool for phenotyping of root hairs traits. The rationale of the work is clear: namely that manual root hair phenotyping is challenging and time consuming, while existing computational tools require input or long processing times. pyRootHair tries to alleviate this phenotyping bottleneck. The dataset is appropriate and validation against manual measurements shows good reliability of the method. The code and Jupyter notebooks are available on GitHub under MIT license. At the same time, some issues as stated below need to be addressed before publication.

Major comments
1.) Figure numbering and referencing: Across the manuscript, figure numbers are not always in the same order as they are referenced in the text. This creates unnecessary confusion for the reader and should be corrected. For example, the first referenced figure is Figure 3 in line 114. The same happens with supplementary figures. This

creates confusion and should be corrected.

The figure order in the manuscript has been amended such that figures appear in order as referenced in text.

2.) Section 4 Data Description: This section only shortly describes the dataset of the wheat images, but does not include the images of the other species that were used for training and evaluation. These additional images are, however, used for analysis in Section 5.2 and 5.3. The manuscript would benefit from elaborating on the description of all data (e.g. different magnification, microscopes) used and include all images in this section.

More detail has been added in Section 4 to include other species. Oat,tomato, rice and teff images were acquired under the same experimental setup as all the wheat images.

3.) Lines 121-123: The manuscript mentions that images that contain a longer section of root would have a higher mean RHL. Is this a mistake or can the authors elaborate on this?

This is not a mistake – the manuscript (and pyRootHair) assumes that input root images will contain the root tip in view, with the root pointing downwards. As such, if an image contains a short section of root (i.e there is a large amount of whitespace between the root tip and the bottom image border), then only a small section of root hairs will be present in the image, thus the overall mean RHL for the image will be shorter due to less hairs being present in frame within the mature zone. In contrast, if an image has a long section of root (i.e the root tip is close to the bottom image border), the image will have included a large section of root hairs from the mature zone, which are fully elongated, and thus will produce a larger mean RHL for that image. As such, the length of root present in the input image will affect mean RHL measurements. For this reason, I have included –length-cutoff, which allows standardization of all measurements for a given batch of images to be performed.

4.) Section 5.1 Variation in Root Hair Traits : The results are presented as a very large set of traits, but often without synthesis. At present, the section does not feel like an analysis. The authors should highlight and discuss the most biologically meaningful traits and better integrate the findings into a interpretation.

The Analyses section has now been split into 4 distinct categories; root hair traits, root traits, root hair heterogeneity traits, and elongation zone traits. Each sub category now corresponds to it’s own figure, which hopefully increases clarity and readability. Each section has also been expanded to include more biological insights.

5.) The method to calculate RHD is described in section 5 Analysis section. I believe it would fit better into the Section 8 Methods. This would also improve the readability of section 5.

The section on RHD calculation has been moved to section 8.4.

6.) I disagree with how the authors use the term plasticity. Plasticity usually relates to the change in a trait due to environmental conditions. Since no environmental factors were used in this study it should not be used. I think that developmental variability or heterogeneity are more suitable in the context of this manuscript.

All mentions of ‘root hair plasticity’ have been amended to ‘root hair heterogeneity’.

7.) From Section 4 and Section 8.2 it appears as if different microscopes (Leica SP9 and Leica S9D) were used. If this is a typo it should be corrected.

This typo has been rectified (Leica S9D for both sections).

8.) Section 5.2 Adaptability: Are there any results for medicago, brachypodium and arabidopsis? If not, the authors should either add the results or do not state that they investigated these species.

Powered by Editorial Manager® and ProduXion Manager® from Aries Systems Corporation

Medicago, arabidopsis and brachypodium images were sent by Guichard et al 2019. I was unable to locate a scale bar to convert the pixel data to mm for those images, thus I did not include the data in the current manuscript. The medicago, arabidopsis and brachypodium images were only used as training instances for the nnUNet segmentation model. I have amended section 5.2: '... as well as further investigation in single accessions from four other species: oat, rice, teff and tomato, and removed mention of the 3 species from the introduction.

9.) Section 5.4 Performance: Can you elaborate on what "extracted summary data" means?

'Extracted summary data' was not the correct term and has been removed. I have reworded this sentence to:

'Despite the reduction in segmentation accuracy with the RFC model, the output data of the random forest pipeline displayed a relatively linear relationship with the output data generated from the main pipeline using a GPU. '

10.) Section 6 Discussion: The critique of DIRT/μ could be framed more constructively. While pyRootHair emphasizes speed and throughput, DIRT/μ aims at accurate resolution of individual hairs. These represent different goals, and a fairer discussion would acknowledge the trade-offs rather than dismissing the alternative approach.

I apologize for the initial wording; the intention was not to critique DIRT/μ at all. This section has been reworded as follows, which I hope offers a fair reflection of both software packages:

'While DIRT μ accurately resolves the length of individual root hairs at the cost of computational throughput (Pietrzyk et al., 2025), pyRootHair quantifies overall root hair morphology of an image at high speeds with lower hardware demand, while sacrificing some individual root hair resolution. Our approach offers increased throughput, and the fully automated nature of the package avoids any user selection biases towards longer root hairs.'

11.) Section 8.2 Image Acquisition: Most of this paragraph deals with plant material rather than the imaging process. This part should be moved to Section 8.1.

Section 8.1 and 8.2 have been merged to form '8.1 Plant Material and Image Acquisition' for clarity.

12.) Section 8.5 Workflow and Figure 1: The workflow was difficult to understand and there are a few mistakes in the text/figure. Steps 9-10 are omitted in the text and the description for Steps 12-15 are described as single step within one sentence. Please elaborate on that. In Figure 1 the text "Cubic spline interpolation on rotated root skeleton co-ordinates" is used between steps 9-10 as well as 10-11. I would also prefer to have a visual presentation of the steps to clarify some of the steps.

The original pipeline Figure (1) has now been replaced with Figure (10), which is a much more simplified version explaining the brief pipeline stages for non-technical readers, but still covers the key steps involved. I have also added Supplementary Figures 8 and 9, which provide a graphical representation of most of the pipeline stages, including root extraction, rotation, straightening via affine transformation, and root hair mask extraction. The S Figures have been referenced in text in Section 8.4 Workflow, which hopefully aids clarity by providing a visual representation.

13.) Figure 3: The figure is overcrowded and very difficult to follow. The order of subplots does not match the order in which they are introduced in the text, which makes it hard for the reader to connect text with subplots. The figure would benefit from being split into multiple, thematically grouped panels, with subplots reordered to match the text.

The original figure has been split into 4 separate figures, grouped by theme (Root hair traits, root traits, elongation zone traits and root hair heterogeneity traits).

	14) Figure 3/4: It would be also beneficial for easy comparison if the order of the traits is the same in both figures.<br><br>The order of traits in the correlation matrix has been corrected to sequentially follow the order of traits shown in the 4 sets of box plots.<br><br>15) The manuscript uses the word "extremely" several times (eight times in total), which reduces scientific objectivity. I recommend rephrasing with more precise or neutral wording (e.g., "computationally intensive" instead of "extremely computationally expensive").<br><br>6/8 instances of ‘extremely’ have been removed, or replaced with neutral wording. For example, Line 228 has been reworded from ‘...pyRootHair is extremely quick...’ to ‘...pyRootHair has a low per-image processing time...’.<br><br>Minor comments<br>16) Mean RHL and average RHL are used interchangeably throughout the text and in Table 2. Please choose one and correct.<br><br>All instances of ‘average’ have been changed to ‘mean’ in text and Table 2.<br><br>17) Line 26: Eragostis tef is misspelled. Should be Eragrostis tef.<br><br>This typo has been rectified<br>18) Line 88: DIRTμ should be written DIRT/μ<br><br>This typo has been rectified<br>19) Line 118: Add the value of the correlation between mean RHL and maximum RHL: i.e. (Figure 4, R^2=???)<br><br>R^2 value has been added<br>20) Line 130: "Supplementary 2B" should be "Supplementary Figure 2B"<br><br>This has been corrected<br>21) Line 140: The term 'positive growth' is possibly unsuitable to describe the elongation zone. I would suggest: "…where root hairs increase length."<br><br>This has been reworded to ‘...largest continuous region where root hair length increases...’<br>22) Line 147: The sentence "All cultivars were imaged five days after germination." would fit better into the data description or methods sections<br><br>This has been moved to the end of Section 8.2<br>23) Line 153: Figure 3M should be Figure 3N<br><br>This typo has been rectified<br><br>24) Line 181: Is the correlation between automated and manual measurements for RHL or mean RHL?<br><br>The correlation is for RHL, not mean RHL. Mean RHL (taken as an average of all RHLs within the image) is calculated from raw RHL (spatial length data along the root). Manual RHL measurements were performed in ImageJ across the validation images, at 0.1mm intervals along the root. This data was compared to the automated length measurements from pyRootHair and correlated. This has been expanded on in text (Line188-189)<br><br>25) Line 256 and other (plus Figure 2 caption): The term binary mask is used even though three classes are present. From my understanding a binary mask only has zeros and ones. It should therefore not be called binary.<br><br>‘Binary’ has been replaced with ‘Segmentation’ for accuracy.<br>26) Section 8.6 Output: Be consistent with the format for arguments/flags (bold or not). E.g. -i/--input is bold while --plot-summary is not

Powered by Editorial Manager® and ProduXion Manager® from Aries Systems Corporation

	All flags/arguments have been un-bolded.<br>27) Line 365: GWAS was not used as abbreviation in the text<br><br>GWAS has been fully abbreviated in text, and removed from the abbreviation section<br>28) Table 1: Is DEU used for Germany? Explain the country abbreviations used in the caption<br><br>DEU is Germany, the country codes are used in line with the IBAN Alpha-3 (https://www.iban.com/country-codes) guidelines. The table caption has been updated to clarify this.<br>29) Table 2: Why is "Avg RHL" used here? Could you be consistent with the rest of the text<br><br>Avg RHL has been corrected to Mean RHL.<br>30) Figure 2: In the caption the vertical dashes lines are described as "maroon", but in the text (lines 141) they are described as "red". Also, the horizontal dashed line in the image looks purple, but in the caption it is described as "orange".<br><br>The colour mismatches have been corrected in text and in the figure caption.<br><br>--<br><br>Once again, I would like to thank all the reviewers for their feedback. I hope that my responses have suitably addressed all reviewer concerns regarding the manuscript.<br><br>Kind Regards,<br>Ian Tsang
**Additional Information:**	
**Question**	**Response**
Are you submitting this manuscript to a special series or article collection?	No
**Experimental design and statistics**<br><br>Full details of the experimental design and statistical methods used should be given in the Methods section, as detailed in our [Minimum Standards Reporting Checklist](). Information essential to interpreting the data presented should be made available in the figure legends.<br><br>Have you included all the information requested in your manuscript?	Yes
**Resources**<br><br>A description of all resources used, including antibodies, cell lines, animals and software tools, with enough information to allow them to be uniquely	Yes

identified, should be included in the Methods section. Authors are strongly encouraged to cite Research Resource Identifiers (RRIDs) for antibodies, model organisms and tools, where possible.<br><br>Have you included the information requested as detailed in our Minimum Standards Reporting Checklist?	
**Availability of data and materials**<br><br>All datasets and code on which the conclusions of the paper rely must be either included in your submission or deposited in publicly available repositories (where available and ethically appropriate), referencing such data using a unique identifier in the references and in the "Availability of Data and Materials" section of your manuscript.<br><br>Have you have met the above requirement as detailed in our Minimum Standards Reporting Checklist?	Yes
GigaScience has policies and guidelines in place for the use of generative AI-writing tools such as ChatGPT. If you have used such writing tools to assist with writing the manuscript this must be declared and cited in the text. Authors should not list AI-writing tools and other AI-assisted technologies as an author or co-author and should acknowledge that they are fully responsible for text generated or refined by AI-writing tools.<p><br><br>A summary of use (particularly in the introduction or among methods) needs to be included at the end of the paper, and the outputs should also be included as a supplementary file hosted in GigaDB or other open repositories. Please read our guidelines for more information. <p><br><br>By submitting to GigaScience, you are aware of the journal's AI-writing tools policy, and if you have declared use of	No

such tools below, you have acknowledged this where appropriate in your manuscript and have made a summary of use and outputs available. <p> <b>AI-assisted writing tools have been used in the preparation of this manuscript?	

Manuscript

Click here to access/download;Manuscript;pyRootHair_Paper_Revised.pdf

pyRootHair: Machine Learning Accelerated Software for High-Throughput Phenotyping of Plant Root Hair Traits

Ian Tsang[1,2,*], Lawrence Percival-Alwyn[1], Stephen Rawsthorne[3], James Cockram[1], Fiona Leigh[1,†], and Jonathan A. Atkinson[2,†]

[1] Niab, Park Farm, Villa Road, Histon, Cambridge, CB24 9NZ, UK
[2] University of Nottingham, Plant Sciences Building, Sutton Bonnington Campus, Nottingham LE12 5RD, UK
[3] The Morley Agricultural Foundation, Morley Business Centre, Deopham Road, Morley St Botolph, Wymondham NR18 9DF, UK

† Joint last authorship.

* Corresponding author: ian.tsang@niab.com

1 Abstract

Root hairs play a key role in plant nutrient and water uptake. Historically, root hair traits have largely been quantified manually. As such, this process has been laborious and low-throughput. However, given their importance for plant health and development, high-throughput quantification of root hair morphology could help underpin rapid advances in the genetic understanding of these traits. With recent increases in the accessibility and availability of artificial intelligence (AI) and machine learning techniques, the development of tools to automate plant phenotyping processes has been greatly accelerated. Here, we present pyRootHair, a high-throughput, AI-powered software application to automate root hair trait extraction from microscope images of plant roots grown on agar plates. pyRootHair is capable of batch processing over 600 images per hour without manual input from the end user. In this study, we deploy pyRootHair on a panel of 24 diverse wheat (*Triticum aestivum* and *Triticum turgidum ssp. durum*) cultivars and uncover a large, previously unresolved amount of variation in many root hair traits. We show that the overall root hair profile falls under two distinct shape categories, and that different root hair traits often correlate with each other. We also demonstrate that pyRootHair can be deployed on a range of plant species, including oat (*Avena sativa*), rice (*Oryza sativa*), teff (*Eragrostis tef*) and tomato (*Solanum lycopersicum*). The application of pyRootHair enables users to rapidly screen a large number of plant germplasm resources for variation in root hair morphology, supporting high-resolution measurements and high-throughput data analysis. This facilitates downstream investigation of the impacts of root hair genetic control and morphological variaton on plant performance.

2 Keywords

Root Hairs, Plant Phenotyping, Machine Learning, Computer Vision, AI, U-Net, Wheat, Roots, Software

3 Background

Root hairs are single cell projections that can develop on all root surfaces (Grierson et al. 2014). They play an important role in facilitating nutrient and water uptake in plants (Dolan 2017, Zhang et al. 2018) and also help to maintain root-soil cohesion (De Baets et al. 2020) and plant anchoring to soil (Haling et al. 2013). The ability of root hairs to project outwards against high soil pressure has led researchers to use root hair cells (trichoblasts) as a model system to study plant responses to mechanical resistance (Pereira et al. 2024). Despite their importance, the morphological variation and genetic control of root hairs remain relatively under-explored compared to other root traits (Tsang et al. 2024a). This is largely due to the inherent challenges of phenotyping root hairs, which are small in size and typically obscured by soil.

Most traditional root hair phenotyping techniques are relatively straightforward. Roots are typically grown on transparent agar plates, imaged after a few days, and the root hair measurements are manually recorded from microscope images. While this method works for small-scale studies, manual measurement of root hair traits is extremely time consuming, becomes prohibitively expensive in larger studies, and is often prone to user bias. Studies often select low numbers of root hairs to measure length at pre-defined distances from the root tip (Liu et al. 2018, Bahmani et al. 2016), and measurements are typically performed using the software FIJI (Schindelin et al. 2012, Saengwilai et al. 2021, Vatter et al. 2015, Stetter et al. 2015). Other methods involve selecting fully elongated root hairs from the mature zone of the root (Pacheco et al. 2022, Maqbool et al. 2022, Huang et al. 2020). While this method is less time consuming, it fails to provide spatial information on root hair length relative to the root tip, and constrains measurements to a specific zone of the root. Furthermore, the manual selection of the 'longest' root hairs per image introduces a large amount of user bias, both in terms of selection and measurement. Since the acquired image is a two-dimensional (2D) projection of a three-dimensional (3D) object, some 'long' root hairs may appear longer or shorter in the image than in reality. An alternative growth method is the 'cigar roll' technique (Liu et al. 2021, Maqbool et al. 2022), whereby seedling roots are grown on moist germination paper, and the paper subsequently rolled into a cigar shape for subsequent plant growth. While possibly faster to set up than growing on agar, this method may inflict damage to the delicate root hairs upon unrolling of the 'cigar' prior to imaging.

For root hair density measurements, studies often perform manual counting of individual root hairs in a defined root zone (Bahmani et al. 2016, Stetter et al. 2015). These methods are very laborious and time consuming, and are not suitable across all plant species. While the root hairs of the model dicotyledonous species *Arabidopsis thaliana* are relatively sparse and individually identifiable (Choi et al. 2019), other species such as the cereal crop bread wheat (*Triticum aestivum*) (Tsang et al. 2024b) or the nitrogen fixing model species *Medicago truncatula* (Guichard et al. 2019) have denser root hairs, meaning manual counting is simply not feasible. This principle also applies to manually selecting root hairs to measure, where in species with dense root hairs, determining the start and end of individual root hairs is challenging and often impossible.

More complex methods for root hair phenotyping have also been described. For example, X-ray computed

tomography (CT) has been used to model phosphate uptake in wheat root hairs (Keyes et al. 2013) and nutrient movement in the rhizosphere of rice (*Oryza sativa*) (Daly et al. 2016), providing extremely detailed characterization of root hairs in soil. Scanning and confocal microscopy techniques have also been deployed, in combination with microfludic platforms, to visualize root hairs with minimal disruption. These techniques have been used to quantify and study trichoblast nuclei at the cellular level (Aufrecht et al. 2017, Yan et al. 2021, Singh et al. 2021, Brueggeman et al. 2022). While these techniques provide a vast amount of data at a high resolution, the equipment required is expensive, and the methods are both computationally and labour-intensive, making them unsuitable for medium-to-large scale screens of root hair morphology.

While a number of semi-automated methods for root hair phenotyping have been described, these remain limited in the throughput that can be achieved. Vincent et al. 2017 developed a semi-automated image analysis program that quantified root hair density (RHD) from images of plants grown in *in-situ* systems (e.g. rhizotrons - glass fronted soil filled chambers that allow root observation over time), which required users to manually trace outlines of their images for training. Lu et al. 2022 developed similar *in-situ* software to segment root hairs from plant roots grown in mini rhizotrons. Their method used a convolutional neural network (CNN) to perform image segmentation, enabling subsequent extraction of root hair length, diameter and area. Guichard et al. 2019 developed the software RootHairSizer, a high-throughput (42 images per hour), ImageJ-based algorithm for semi-automated measurements of root hair length, growth rate, and the differentiation zone from agar-grown images of roots. In RootHairSizer, users are required to manually define the following: the bounding regions along the root for measurement, the thresholding method and the measurement resolution. Recently, Pietrzyk et al. 2025 developed DIRT/μ, a python-based software that utilizes machine learning to disentangle and measure individual root hairs, regardless of root hair density in the input image. However, this software is computationally expensive, and potentially unsuitable for large-scale screening experiments.

Despite the advances in root hair phenotyping tools summarized above, their inability to automatically process large numbers of images with minimal manual intervention makes them potentially ill-suited to large-scale screening of root hair traits. Here, we present pyRootHair, a novel and fast software application designed for high-throughput root hair phenotyping. Primarily, pyRootHair uses a CNN for rapid and accurate image segmentation with a graphical processing unit (GPU), while also providing a simple random forest classifier (RFC) pipeline for an alternative segmentation method. For each individual image, pyRootHair extracts up to 15 summary traits and provides high spatial-temporal resolution of root hair length and area. To demonstrate its utility, we used pyRootHair to uncover significant varietal variation in all root hair traits measured across a panel of diverse wheat cultivars, and define multiple uniquely identifiable traits, including 'root hair profile'. We further demonstrate the effectiveness of pyRootHair in processing root hair images by exhibiting its use across a range of plant species, and validate the automatically extracted traits relative to manual measurements. pyRootHair resolves a previously persistent bottleneck in root hair phenotyping. Its ability to uncover a wealth of previously undiscovered root hair phenotypic diversity across different plant species and populations, will enable future genetic and physiological studies aimed at further understanding key aspects of the 'hidden half' of the plant.

4 Data Description

The root hair dataset used consisted of 252 wheat seedling root images. Seeds for the 24 wheat cultivars (listed in Table 1), plus seed of 1 accession each of rice, tomato, teff and oat were surface sterilized and germinated on agar plates for 5 days. All root images were captured via light microscopy (Leica S9D) at 0.6× magnification and processed using pyRootHair.

5 Analyses

5.1 Variation in Root Hair Related Traits

5.1.1 Root Hair Traits

When using pyRootHair to extract root hair phenotypic data from images across a panel of 24 wheat cultivars, a large amount of variation was observed for all extracted root hair traits extracted (Figure 1). Mean root hair length (RHL) varied between 1-2 mm for most cultivars. Within the bread wheat cultivars, Bersee had notably shorter root hairs relative to all other accessions (Figure 1A). The durum wheat (*Triticum turgidum ssp. durum*) varieties Dakter and Kofa had shorter mean RHL than most bread wheat genotypes, potentially indicating the D genome in hexaploid wheat may contribute more to RHL relative to the A and B genomes of tetraploid wheat. Mean RHL exhibited a strong positive correlation with maximum RHL (Figure 2, $R^2 = 0.74$, Figure 1B). Total RHA (Figure 1C) was positively correlated with mean RHL (Figure 2, $R^2 = 0.66$). This was unsurprising, as RHA was simply measured as the 2D area occupied by the root hair mask. As such, images that contained a longer section of root would have a higher mean RHL due to the inclusion of more root hairs in the mature zone of the root, and thus, a higher RHA. The RH:Background Pixel Ratio served as a proxy for RHD (Figure 1D, Supplementary Figure 1B). Interestingly, no correlation was observed between total RHA and RHD (Figure 2, $R^2 = 0.1$). This indicates that cultivars with a low RH:Background pixel ratio (a proxy for high RHD, e.g. Rialto) may have high root hair density along the 3D root cylinder, but when projected onto a 2D plane (e.g. in an image), the increased root hair density does not result in increased root hair surface area (RHA). Since the RHD proxy is measured from pixel intensity, variation in image lighting quality would also affect this measurement, potentially resulting in mismatched correlations with total RHA.

5.1.2 Root Traits

The thickness of the captured root (i.e mean width of the root excluding root hairs) (Figure 3A) varied across all cultivars. Root thickness exhibited a strong positive correlation with mean RHL (Figure 2, $R^2 = 0.72$), elongation zone gradient (Figure 2, $R^2 = 0.60$), andnegatively correlated with the elongation zone end position (Figure 2, $R^2 = -0.55$). In addition to having a low mean RHL, the tetraploid cultivars Dakter and Kofa also displayed very narrow roots (Figure 3A). Root length variation between cultivars and individuals was also observed (Figure 3B). This was likely due to underlying genetic differences, plate and positional growth room effects. As such, each image captured was positioned to contain the maximum length of root suitable for downstream processing. Thus, for cultivars with a consistent level of root growth (e.g. Gladiator,

Spark, Stetson), there was minimal variation in captured sample root length. Conversely, cultivars with a high degree of variation in root length (e.g. Brigadier, Copain, Kloka) displayed large variation in captured sample root length (Figure 3B).

5.1.3 Root Hair Heterogeneity Traits

Here, the trait 'root hair heterogeneity' was determined as the variation in root hair length and area within an individual root image (Figure 4). Bread wheat cultivars, including Brigadier, Brompton, Hereward and Soissons, all exhibited large fluctuations in max RHL δ between the left and right root hair segments (Figure 5A). The position along the root of maximum RHL fluctuation also varied between cultivars (Figure 5B), but no linear relationship with Max RHL δ was found. In contrast, some cultivars (notably Robigus, Spark and Steadfast) exhibited minimal variation in RHL δ between individuals, indicating these cultivars had more uniform root hair growth. Max RHA δ (Figure 5C) and Max RHA δ pos (Figure 5D) measured the amount of RHA difference between the left and right root hair segments of an image, and the corresponding position of this difference respectively. Max RHL δ was strongly correlated with max RHA δ (Figure 2). Since the root hair heterogeneity traits exhibited here are measuring absolute difference in length and area instead of taking a mean value of an image, these traits are very sensitive to the accuracy of the segmented image, and should thus be used with caution.

5.1.4 Elongation Zone Traits

The elongation zone was defined here as the largest continuous region where root hair length increases (Figure 4, region bound by the vertical dashed maroon lines). Notably, the bread wheat cultivar Spark had a very large elongation zone size (Figure 6A). Three cultivars (Claire, Copain and Kloka) all exhibited wide variation in elongation zone size between biological replicates. The starting position of the elongation zone was relatively consistent between all cultivars, in close proximity to the root tip (Figure 6B). In contrast, the end position of the elongation zone varied more relative to the start position (Figure 6C), as highlighted by the high correlation between the elongation zone distance and end position (Figure 2, $R^2 = 0.78$). The gradient of the elongation zone (Figure 6D) was negatively correlated with the stop position (Figure 2, $R^2 = -0.71$) and distance (Figure 2, $R^2 = -0.76$).

The greatest variation in overall root hair profile was found around the root tip, where root hairs had begun to emerge. As such, this diversity in root hair morphology around the root tip may likely play an important role in affecting nutrient uptake. A large degree of variation was found for both root hair emergence (minimum distance from root tip where hairs begin emerging) and overall root hair profile across the 1 cm section from the root tip (Figure 7A). Unsupervised clustering of the root hair profiles revealed two major clusters, termed here 'Shallow' and 'Steep' (Figure 7B). Cultivars in the 'Shallow' cluster (including Kofa, Bersee, Claire) had a shallower root hair profile gradient (Figure 6D, 7A, Supplementary Figure 1A). In contrast , cultivars in the 'Steep' cluster (including Flamingo, Cordiale, Steadfast) had a steeper root hair gradient, and generally had longer root hairs around the root tip compared to cultivars in the 'Shallow' cluster (Figure 7A, Supplementary Figure 1A). Of particular note, Cordiale, Steadfast, Claire all originated from the same country (GBR, Table 1), and exhibited vastly different root hair profiles (Cordiale and Steadfast: steep, Claire: shallow) (Figure 7A). As such, this indicated that root hair morphology may not be strongly

influenced by soil type and climate, but rather more by genetic control.

5.2 Adaptability

Given that overall root morphology is relatively consistent throughout many higher plant species, the methodology deployed in the pyRootHair workflow can be translated to a wide range of wild and cultivated plant species. Here, we demonstrated that pyRootHair can accurately segment and quantify root hair traits by detailed investigation of wheat, as well as further investigation in single accessions from four other species: oat, rice, teff, tomato (Figure 8, Supplementary Figure 2).

5.3 Trait Validation

To validate traits extracted by pyRootHair, the RHL, elongation zone length and root length were manually measured across different wheat images. Manual RHL measurements were taken from five different images, with measurements performed at 0.1 mm intervals along the root across both root hair segments. Manual measurements of the elongation zone and root length were taken from 54 and 68 different images respectively. Validation of RHL measurements in pre-straightened (prior to affine transformation, Supplementary Figure 9A) and straightened (post affine transformation, Supplementary Figure 9C) roots were performed on 15 images. All manual measurements were calibrated using the conversion factor of 102 pixels per 1 mm. Additional validation of RHL measurements were performed in oat, rice, teff and tomato ($R^2 = 0.91$, Supplementary Figure 2).

Strong positive correlations between automated and manual measurements were found for RHL along the root (Figure 9A, $R^2 = 0.88$) and root length (Figure 9B, $R^2 = 0.97$). RHL measurements from pre-straightened and straightened roots exhibited a strong correlation (Figure 9C, $R^2 = 0.9$). The CNN model exhibited accurate segmentation of input images compared to manually annotated segmentation masks, with intersection over union (IoU) scores > 0.8 across different example images (Supplementary Figure 4). Automated measurements of the elongation zone length correlated less well with manual measurements (Figure 9D, $R^2 = 0.55$). While traits such as RHL and root length were simple to manually measure, estimating the elongation zone length was significantly more subjective due to small fluctuations in the root hair profile. The method of elongation zone measurement deployed in pyRootHair selects the largest continuous region of constant root hair growth, as determined by the gradient of the root hair profile (Supplementary Figure 3). This approach of utilizing the gradient to select the elongation zone is more robust than visual quantification. However, this approach does not discriminate against biological anomalies, such as roots with bald patches near the root tip, and may flag a region far from the root tip as the elongation zone. As such, end user validation of output data is critical.

5.4 Performance

Across all images for the 24 wheat cultivars, the mean per-image processing time (including inference) was 7.82 s with an Nvidia L40S GPU with 8 Gigabytes (GB) of Video Random Access Memory (RAM), compared to 7.97 s per image using the RFC pipeline (Supplementary Figure 5A) on a compute node with 20 GB RAM. The mean processing time per image was 430 s when using the CNN to perform inference without

a GPU (Supplementary Figure 5A), which was significantly slower than either the GPU or RFC pipelines (Supplementary Figure 5B). No significant difference in mean image processing time was found between the two pipeline configurations. Since all input wheat images were obtained from the same imaging setup, the image quality and lighting remained consistent. As such, for this comparison, an RFC model was trained on a single input image, and used to perform inference on all subsequent images. Naturally, the simple RFC model produced less refined segmentation masks of input images compared to the CNN deployed in the main pipeline. Despite the reduction in segmentation accuracy with the RFC model, the output data of the random forest pipeline displayed a relatively linear relationship with the output data generated from the main pipeline using a GPU (Supplementary Figure 5C, D).

6 Discussion

Advances in computer vision and AI have greatly accelerated the field of plant phenotyping. Here, we present pyRootHair, a rapid and new computer vision-based software application for high-throughput extraction of root hair traits from root images. To our knowledge, pyRootHair extracts more traits per input image than any other available software. It has a low per-image processing time and enables large scale batch processing of images. Importantly, no user input is required during computation, which eliminates user measurement bias. Furthermore, unlike previous methods (Lu et al. 2022), pyRootHair is readily available, simple to install, operate, and is entirely open source. As such, pyRootHair significantly improves the accessibility, speed and accuracy of root hair phenotyping. The throughput achievable with pyRootHair means that the bottleneck in acquiring root hair data has transitioned from the phenotyping step to the plant growth and image acquisition process.

The limitations of pyRootHair primarily relate to the curvature of root hairs, and the segmentation performance. While straightening of the input root is effective at standardising the measurement along each root hair segment, RHL is calculated as the width of the root hair segmentation mask, and does not account for the inherent curvature within the individual root hairs. While DIRT/μ accurately resolves the length of individual root hairs at the cost of computational throughput (Pietrzyk et al. 2025), pyRootHair quantifies overall root hair morphology of an image at high speeds with lower hardware demand, while sacrificing some individual root hair resolution. Our approach offers increased throughput, and the fully automated nature of the package avoids any user selection biases towards longer root hairs. Secondly, accuracy and effectiveness of trait extraction is dependent on the segmentation performance. While the CNN deployed by the main pipeline is flexible and powerful, training instances are laborious to annotate, and cannot be generated to cover all examples of end user images. The RFC pipeline is computationally inexpensive, but far less flexible compared to the main pipeline with the CNN.

The variation in root hair morphology identified across the 24 wheat cultivars investigated here highlights useful entry points for future genetic and physiological investigation of root hair morphology on plant performance. In particular, traits such as the shape of the root hair profile and heterogeneity have, to our knowledge, not been previously quantified. Identification and formalization of these new traits increases the likelihood of identifying genetic loci and genes controlling root hair morphology via forward genetic analyses, and for their physiological investigation (e.g. via genome wide association studies). For example, given that

root hair length is positively correlated with phosphorous uptake in different crops (Gahoonia et al. 2004, Saengwilai et al. 2021), automated RHL calculation via pyRootHair serves as a convenient and fast method of pre-screening a panel of cultivars for field trials or breeder selection. Future work should investigate the impact of the traits outlined in this study on plant physiology. Examples may include quantifying whether root thickness affects root foraging, or whether elevated RHA and RHD can promote increased nutrient/water uptake across different soil conditions. Future experiments based on data gathered via pyRootHair has the potential to significantly advance our understanding of how root hair morphology affects crop physiology.

7 Potential Implications

Due to the throughput achieved and ease of use, we believe that pyRootHair can significantly reduce the bottleneck in root hair phenotyping, and serve as a key tool for uncovering the genetic loci and genes controlling root hair morphology in crops and other plant species. Through this process, candidate genes controlling root hair traits can be identified and selectively bred into existing populations to develop more resilient, nutrient-efficient crops for the future.

8 Methods

8.1 Plant Material and Image Acquisiton

All plant material is summarized in Table 1. The 24 wheat cultivars were sourced from field-grown trials in Cambridgeshire. The accessions included 22 bread wheat cultivars, encompassing all founder lines within the 'Niab Elite MAGIC' (Multi-Parent Advanced Generation Inter-Cross) and 'Niab Diverse MAGIC' populations (Mackay et al. 2014, Scott et al. 2021). Two durum wheat (*Triticum turgidum* ssp. *durum*) varieties (Dakter, Kofa) were also included. Seeds for oat, rice, teff and tomato were either sourced from locally available seed stocks at Niab or commercially purchased. Medicago, brachypodium and arabidopsis images were provided by Guichard et al. 2019.

Cold-treated seeds (stored for five days at 4°C) were surface sterilized with 70% ethanol for 60 s, then immersed in 20% bleach (sodium hypochlorite) for 10 minutes. Seeds were then washed three times with sterile distilled water in a laminar flow hood, and plated onto square agar plates (Sigma-Aldrich, 120 x 120 x 17 mm) with sterile forceps. Four seeds were placed on each plate, with each seed positioned in a corner with the embryos oriented towards the centre of the plate. Each agar plate contained 50 mL of media prepared with of 0.5 g sucrose (Sigma-Aldrich), 0.15 g phytagel (Sigma-Aldrich) and 0.2 g Murashige and Skoog basal salt (Sigma-Aldrich), mixed with 50 mL dH_2O. Plates were sealed with micropore tape (3M) and placed horizontally on flat racks in a well lit, 25°C growth room for five days under constant lighting. Subsequently, the roots were imaged using a Leica S9D Stereomicroscope at 0.6× magnification, five days after germination.

8.2 Segmentation Model

To generate and train the segmentation model, we used the self-configuring CNN generator nnU-Netv2 (Isensee et al. 2021). Segmentation masks of training instances were manually curated using the interactive segmentation tool ilastik (Berg et al. 2019). For each segmentation mask, pixels associated with the background were labelled with a 0, root hairs as 1, and roots as 2. Training instances varied in input dimensions, clarity and lighting conditions. The training set was composed of 44 wheat, 5 arabidopsis, 14 medicago, 3 brachypodium, 4 teff, 4 maize (*Zea mays*) and 9 rice images, along with the corresponding segmentation masks.

Model training was carried out on the Crop Diversity High Performance Computer (HPC) Cluster (Percival-Alwyn et al. 2024) using an NVIDIA A100 SXM4 80GB Tensor Core GPU. The 'nnUNetResEncUNetMPlans' planner preset was used for all nnU-Net stages (Isensee et al. 2024). Training was conducted with the 'all' fold and the '2d' configuration arguments. Image augmentation was automatically performed by the nnU-Net pipeline. Z-score normalization was carried out for the three input image channels (red, green, blue). The model was trained for 1000 epochs with a batch size of 13. The overall best validation dice score of the model was 98% (Supplementary Figure 6). For root and root hair segmentation masks, mean validation dice scores were 0.97 and 0.99 respectively across all training instances, while mean intersection over union (IoU) scores were 0.84 and 0.97 respectively (Supplementary Figure 7).

8.3 Pipeline Configurations

To accommodate a variety of end-user hardware, pyRootHair was designed to operate across different levels of computing power, including HPC systems and personal computers. As such, different pipeline configurations are available for the end-user.

By default, the main pipeline utilizes a GPU to perform inference on input images. GPU requirements vary depending on input image sizes. For reference, an Nvidia L40S GPU with 8 GB VRAM was sufficient to perform inference on input images of size 2600 x 1500 x 3. Inference without a GPU is still possible on a CPU, but speed will be significantly affected.

For users without a GPU, pyRootHair offers a simple alternative pipeline. Users can easily train an RFC segmentation model on a representative image of their choice with a single command. The trained RFC model can be subsequently used to run inference on input images. Alternatively, end users can generate their own segmentation masks, which can then be individually processed for trait extraction without a GPU.

8.4 Workflow

For a given batch of input images with the main or RFC segmentation pipeline, the predicted masks are generated first. The post-processing steps of each segmentation mask obeys the following methodology for trait extraction:

For each mask, the root is first extracted and segmentation noise removed (Figure 10, Supplementary Figure 8A-C). The root is then skeletonised to a single-pixel wide representation of the root (Figure 10). A spline (smooth curve fitted through a set of points) is subsequently mapped to the skeleton co-ordinates, and the

root midline is approximated via computing the median of each bin in a sliding window down the root mask (Figure 10, Supplementary Figure 8D).

To standardise trait extraction, roots must be oriented downwards in the input image. The orientation of the root in the segmentation mask is calculated from the approximated root midline, and rotated such that the root tip points downwards (Figure 10, Supplementary Figure 9A). The rotated root mask is re-skeletonised and co-ordinates are re-mapped to approximate the midline of the rotated root (Figure 10). Next, to minimize the effect of any inherent root curvature within the rotated root, the entire segmentation mask is straightened via piecewise affine transformation (Van Der Walt et al. 2014), producing a straightened segmentation mask of the original image (Figure 10, Supplementary Figure 9B & C).

After straightening the mask, the root tip is located using kernel convolution along the skeleton of the straightened root (Supplementary Figure 9C), and the root hair masks are extracted on either side of the root (Figure 10, Supplementary Figure 9D).

To extract RHL, the longest RHL is calculated in each bin from a sliding window along each root hair segment, starting at the root tip. The size of the window can be controlled via the --resolution argument. The default value of 20 pixels per bin size was used to compute traits from all images analysed in this paper. RHA is calculated as the total pixel area of root hair mask within each bin. The root hair elongation zone is defined as the region where root hairs experience continuous positive growth. The 'profile' of the root hair segment is modeled using a LOWESS (locally weighted scatterplot smoothing) regression line, and the elongation zone position, size and gradient are extracted from the longest region of the root where the gradient of the root hair profile continuously increases (Figure 4, Supplementary Figure 3B). The heterogeneity of root hair growth is defined as the difference in RHL and RHA between the 'left' and 'right' sides of the root hair segment. The greatest difference in RHL and RHA between each root hair segment are recorded as 'Max RHL/RHA δ', along with the corresponding position along the root (Figure 4). All traits are translated from pixel measurements to mm measurements, where the pixel:mm conversion factor can be controlled via the --conv argument. A full list of extracted traits is available in Table 2.

Root hair area (RHA) was calculated as the total area occupied by the root hairs in each segmentation mask. Root hair density (RHD) was estimated using the RH:Background Pixel ratio (Figure 1D). This ratio was calculated as the mean pixel intensity of the root hairs in the input image, divided by the mean pixel intensity of the background. In backlit images, the lighter background would have a higher mean pixel intensity, while the root hairs would be darker due to their obstruction of light and thus have a lower mean pixel intensity. If a cultivar had more root hairs per unit area of root, the root hairs would be darker, decreasing the pixel intensity value of the root hairs. Thus, assuming consistent lighting across input images, lower RH:Background ratios could indicate a cultivar had higher RHD, while higher RH:Background ratios could indicate a cultivar had lower RHD (Supplementary Figure 1B). Furthermore, the variation in mean background pixel intensity within each cultivar illustrates the consistency of lighting conditions between images, and can therefore serve as a quality control metric.

8.5 Output

For a given batch of input images, pyRootHair produces a summary table and a raw table in comma separated value (CSV) format. The summary table contains the 15 traits listed in Table 2, and is calculated for each image in the input image folder. The raw table contains the individual RHL and RHA measurements from each bin in the sliding window for each input image.

Users can quickly view the summary information displaying RHL and RHA profile for each image via the --plot-summary flag. To visualize the segmentation and transformation of the input image, --plot-segmentation saves the generated segmentation mask of each image. To view how the straightening was performed, --plot-transformation saves a graphical representation of the root warping. To operate pyRootHair, users only need to provide three required arguments: -i/--input specifies the filepath/directory to the input image folder. -b/--batch-id specifies the sub-folder name associated with the current run, which is stored in the output directory. -o/--output specifies a filepath/directory to store the output data and plots.

Since varying root length in the input image affects how summary traits (e.g. Mean RHL, total RHA) are calculated, we offer the argument --length-cutoff, which allows users to specify a length cutoff for input roots (measured in milimetres from the root tip). --length-cutoff standardises trait calculation for all images in the input batch based on the value provided. For example, --length-cutoff 10 will return summary and raw tables of only the first 10 millimetres of the root for all images in the batch. This mitigates a significant portion of bias introduced with varying root length, enabling standardisation of traits for more detailed analysis.

A detailed breakdown of all the arguments and flags is available on the following Github repository `https://github.com/iantsang779/pyRootHair`.

8.6 Dependencies

pyRootHair was written in the Python programming language (v3.12.7) and developed on the Crop Diversity HPC, running the Debian 12 Bookworm operating system (Percival-Alwyn et al. 2024). Scikit-image (v0.24.0) was used to carry out most image processing functionalities. Numerical calculations were carried out using the numpy (v2.0.2) and scipy (v1.14.1) libraries. Data tables were constructed using pandas (v2.2.3) and all plots and figures were created using matplotlib (v3.9.3). LOWESS regression lines were computed using statsmodels (v0.14.4). Scikit-learn (v.1.5.2) was used for quality control of segmented images. nnU-Netv2 (v2.5.1) was used to create the image segmentation model with PyTorch (v.2.5.1) and CUDA (v.12.6).

8.7 Installation

Extensive documentation and source code is available from the github repository: `https://github.com/iantsang779/pyRootHair`. pyRootHair is available on pip for installation: `https://pypi.org/project/pyRootHair/`

9 Availability of Source Code and Requirements

All code used to generate figures for this paper has been made available in the following Jupyter Notebook: https://github.com/iantsang779/pyRootHair/blob/main/paper_data/pyRootHair.ipynb. The source data used to generate figures is available here: https://github.com/iantsang779/pyRootHair/tree/main/paper_data.

- Project name: pyRootHair
- Project home page: https://github.com/iantsang779/pyRootHair
- Operating system(s): Linux , MacOS, Windows
- Programming language: Python
- License: MIT License

10 Declarations

10.1 Competing Interests

The authors declare no competing interests.

10.2 List of Abbreviations

- AI: Artifical Intelligence
- CNN: Convolutional Neural Network
- GPU: Graphical Processing Unit
- HPC: High Performance Computer
- LOWESS: Locally Weighted Scatterplot Smoothing
- RFC: Random Forest Classifier
- RHL: Root Hair Length
- RHA: Root Hair Area
- RHD: Root Hair Density
- VRAM: Video Random Access Memory

10.3 Funding

IT was funded by the Biotechnology and Biological Sciences Research Council (BBSRC) as part of the Collaborative Training Program for Sustainable Agricultural Innovation (CTP-SAI) PhD Programme (BBSRC grant BB/W009439/1), in funded partnership with The Morley Agricultural Foundation (TMAF). A portion

of LP's and JC's time was funded by BBSRC grant BB/X018725/1. A portion of FL's time was funded by BBSRC grant BB/W009439/1.

10.4 Author Contributions

I.T - Conceptualization, Data curation, Formal analysis, Methodology, Software, Validation, Visualization, Writing - original draft, Writing - review & editing. L.P.A - Validation, Writing - review & editing. S.R, J.C, F.L, J.A.A - Supervision, Writing - review & editing.

10.5 Acknowledgements

The authors would like to thank Marjorie Guichard for providing arabidopsis, medicago and brachypodium images for training, and Nina Foreman for providing rice seed stocks. We also thank Mike Pound and Feng Chen for their invaluable advice on CNNs. The authors acknowledge Research Computing at the James Hutton Institute for providing computational resources and technical support for the "UK's Crop Diversity Bioinformatics HPC" (BBSRC grants BB/S019669/1 and BB/X019683/1), use of which has contributed to the results reported within this paper.

11 Tables

**Cultivar**	**Ploidy**	**Species (Common Name)**	**Origin**
Alchemy	Hexaploid	*Triticum aestivum* (Bread Wheat)	GBR
Banco	Hexaploid	*Triticum aestivum* (Bread Wheat)	SWE
Bersee	Hexaploid	*Triticum aestivum* (Bread Wheat)	FRA/GBR
Brigadier	Hexaploid	*Triticum aestivum*(Bread Wheat)	GBR
Brompton	Hexaploid	*Triticum aestivum* (Bread Wheat)	GBR
Claire	Hexaploid	*Triticum aestivum* (Bread Wheat)	GBR
Copain	Hexaploid	*Triticum aestivum* (Bread Wheat)	FRA
Cordiale	Hexaploid	*Triticum aestivum* (Bread Wheat)	GBR
Dakter	Tetraploid	*Triticum turgidum ssp. durum* (Pasta Wheat)	FRA
Flamingo	Hexaploid	*Triticum aestivum* (Bread Wheat)	DNK/NLD
Gladiator	Hexaploid	*Triticum aestivum* (Bread Wheat)	GBR
Hereward	Hexaploid	*Triticum aestivum* (Bread Wheat)	GBR
Holdfast	Hexaploid	*Triticum aestivum* (Bread Wheat)	GBR
Kloka	Hexaploid	*Triticum aestivum* (Bread Wheat)	DEU/DNK/GBR
Kofa	Tetraploid	*Triticum turgidum ssp. durum* (Pasta Wheat)	USA
Maris Fundin	Hexaploid	*Triticum aestivum* (Bread Wheat)	GBR
Rialto	Hexaploid	*Triticum aestivum* (Bread Wheat)	GBR
Robigus	Hexaploid	*Triticum aestivum* (Bread Wheat)	GBR
Slejpner	Hexaploid	*Triticum aestivum* (Bread Wheat)	DNK/SWE
Soissons	Hexaploid	*Triticum aestivum* (Bread Wheat)	FRA
Spark	Hexaploid	*Triticum aestivum* (Bread Wheat)	GBR
Steadfast	Hexaploid	*Triticum aestivum* (Bread Wheat)	GBR
Stetson	Hexaploid	*Triticum aestivum* (Bread Wheat)	GBR
Xi19	Hexaploid	*Triticum aestivum* (Bread Wheat)	GBR

Table 1: **Name, ploidy, common name and country of origin of all the wheat cultivars used in this study.** Country of origin denoted by the IBAN Alpha-3 country code. DEU: Germany, DNK: Denmark, FRA: France, GBR: Great Britain, NLD: Netherlands, SWE: Sweden, USA: United States of America

Trait	Explanation
Name	Name of image file
Batch ID	Batch ID specified via -b/--batch_id
Mean RHL (mm)	Mean root hair length of the input image, calculated across the entire length of the root
Max RHL (mm)	Maximum recorded root hair length in the input image
Min RHL (mm)	Minimum recorded root hair length in the input image
Total RHA (mm^2)	Total root hair area of the input image
Max RHL Delta (mm)	Largest difference in root hair length between each root hair segment on either side of the root
Max RHL Delta Pos (mm)	Position along the root corresponding to the largest difference in root hair length
Max RHA Delta (mm)	Largest difference in root hair area between each root hair segment on either side of the root
Max RHA Delta Pos (mm)	Position along the root corresponding to the largest difference in root hair area
Elongation Zone Distance (mm)	Length of the elongation zone, defined as the largest continuous region where root hairs are emerging
Elongation Zone Start (mm)	The position along the root where the elongation zone starts, calculated as distance from the root tip
Elongation Zone End (mm)	The position along the root where the elongation zone ends, calculated as distance from the root tip
Elongation Zone Gradient	The gradient of the elongation zone, calculated as δ RHL in elongation zone/length of elongation zone
Root Thickness (mm)	Mean thickness of the root, calculated via a sliding window down the segmented root mask
Captured Sample Root Length (mm)	Total length of the straightened root captured within the input image
RH Pixel Intensity Mean	Mean pixel intensity of the area occupied by the root hairs in the input image
Background Pixel Intensity Mean	Mean pixel intensity of the background in the input image
RH: Background Pixel Ratio	RH pixel intensity divided by background pixel intensity

Table 2: **Summary traits calculated for each input image.** RHA = root hair area, RHL = root hair length

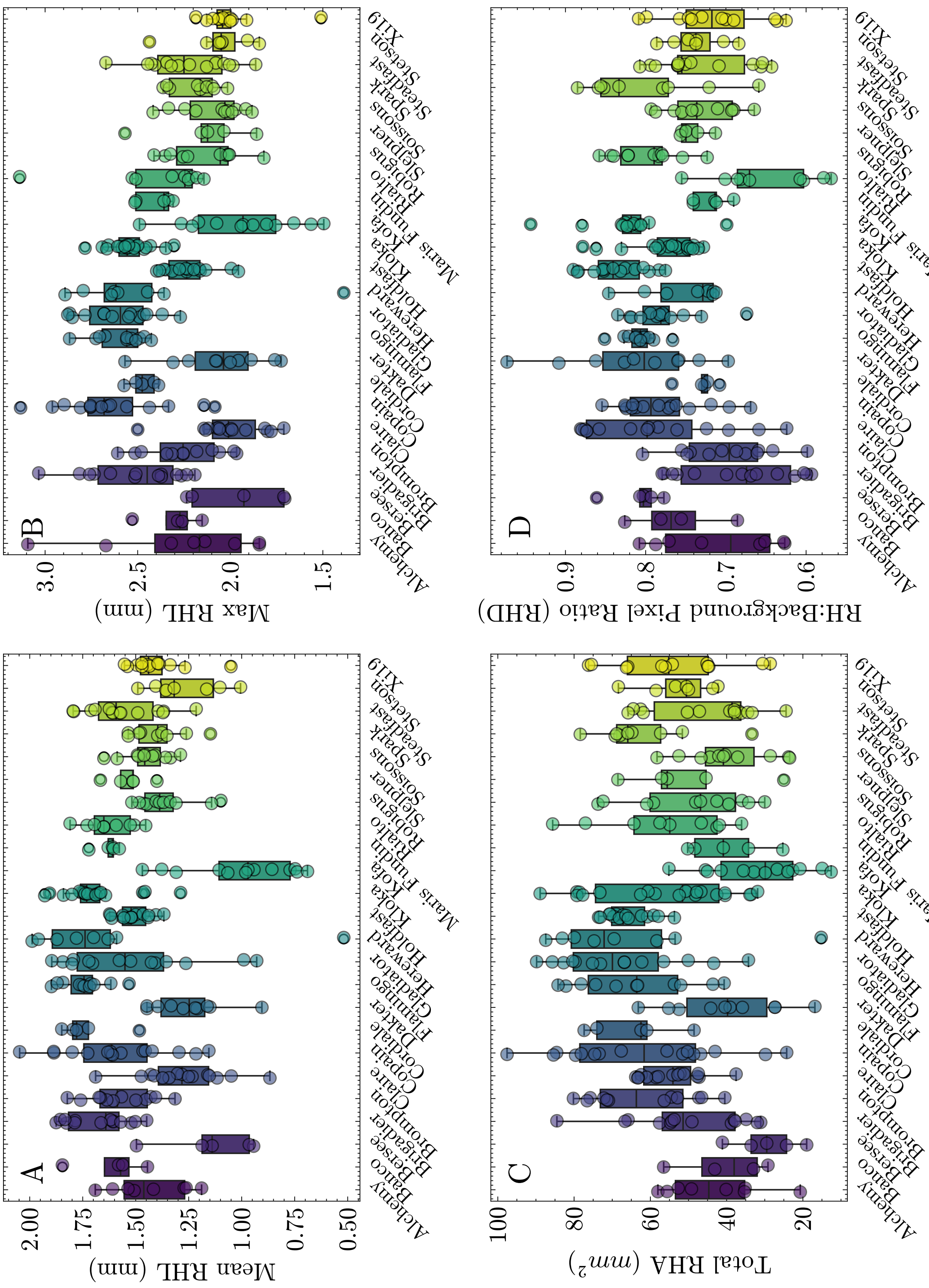

Figure 1: **Root hair traits.** Boxplots illustrate spread of data for each cultivar and each trait. The horizontal lines within each boxplot illustrates the median, whiskers represent the data range, and data from an individual image is displayed as a dot.

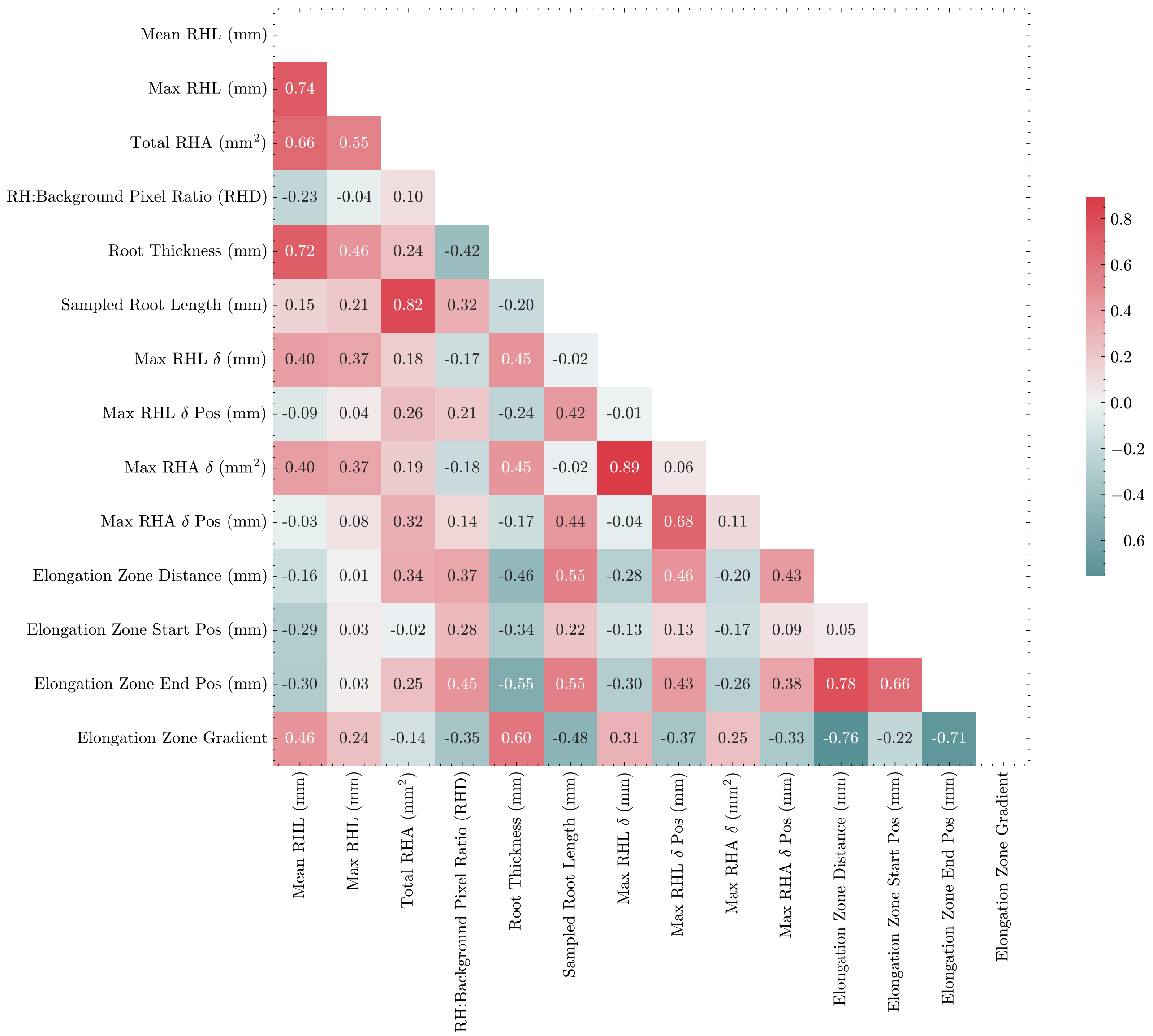

Figure 2: **Correlation matrix illustrating relationships between root hair traits measured across the 24 wheat cultivars using pyRootHair.** Numbers within squares illustrate Pearson correlation coefficients.

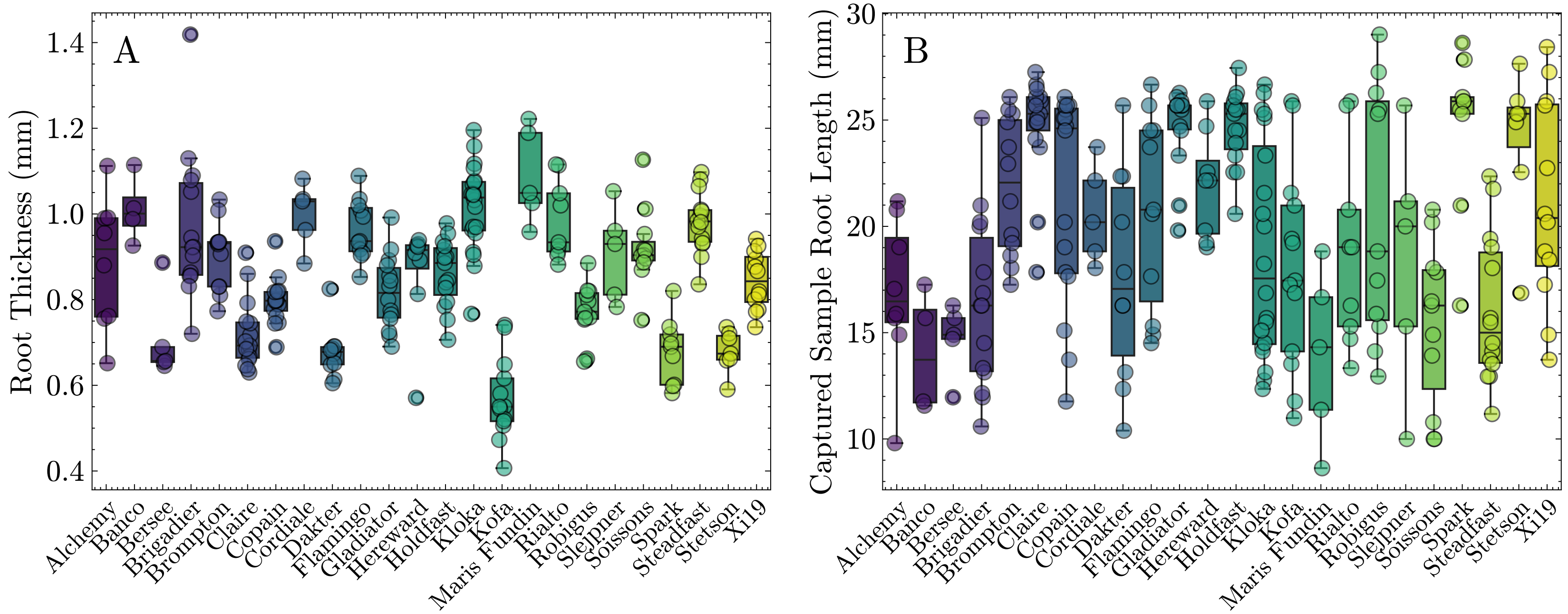

Figure 3: **Root traits.** Boxplots illustrate spread of data for each cultivar and each trait. The horizontal lines within each boxplot illustrates the median, whiskers represent the data range, and data from an individual image is displayed as a dot. RHL: Root Hair Length, RHA: Root Hair Area, RHD: Root Hair Density.

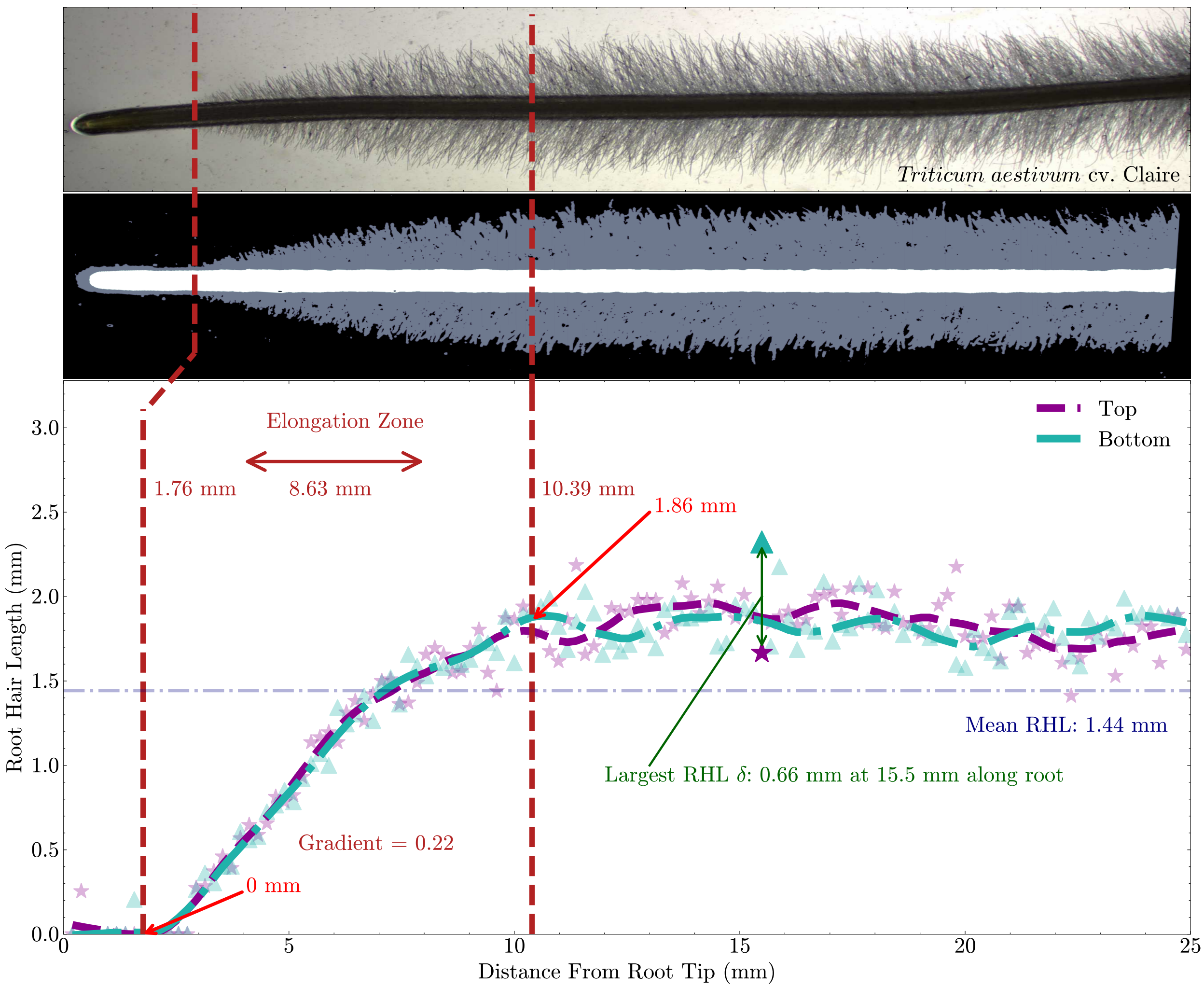

Figure 4: **Visual representation of selected root hair traits extracted from an example bread wheat (cv. Claire) image using pyRootHair.** Top) Raw image. Middle) Segmentation mask of the raw image. Bottom) Green and purple markers illustrate root hair lengths along the root. Length profile for each segment (top or bottom) is illustrated by dashed regression lines through the markers. Vertical dashed maroon lines indicate the automatically identified root hair elongation zone, calculated as the largest region of continuous upward trajectory in the root hair profile. Gradient of the elongation zone is calculated as the maximum change in root hair length within the zone, divided by the length of the zone. Mean root hair length is calculated across the entire root length, illustrated by the dashed horizontal light purple line. The maximum difference (δ) in root hair length between the root hair segments is 0.66 mm at 15.5 mm away from the root tip.

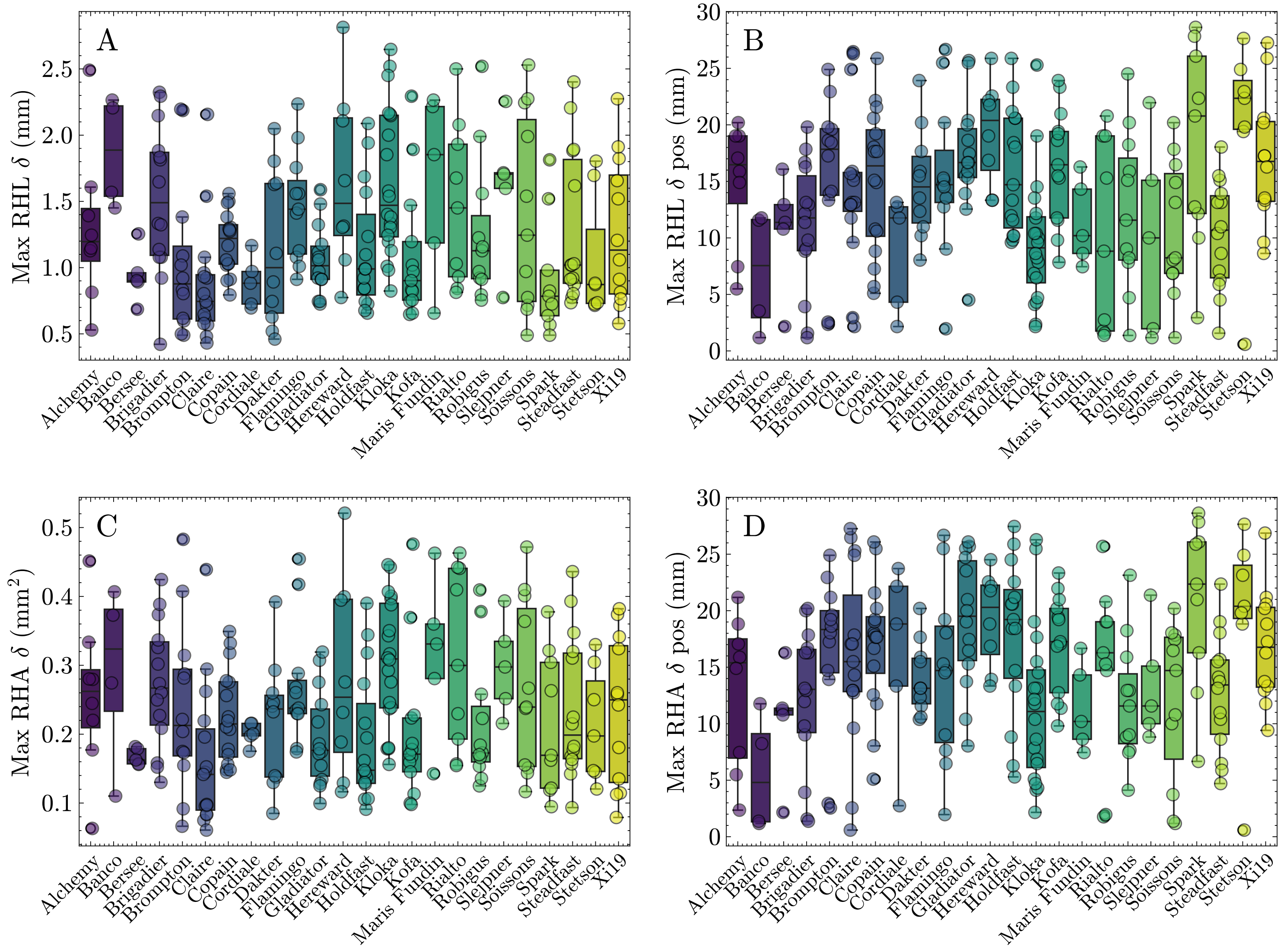

Figure 5: **Root hair heterogeneity traits.** Boxplots illustrate spread of data for each cultivar and each trait. The horizontal lines within each boxplot illustrates the median, whiskers represent the data range, and data from an individual image is displayed as a dot. δ: difference

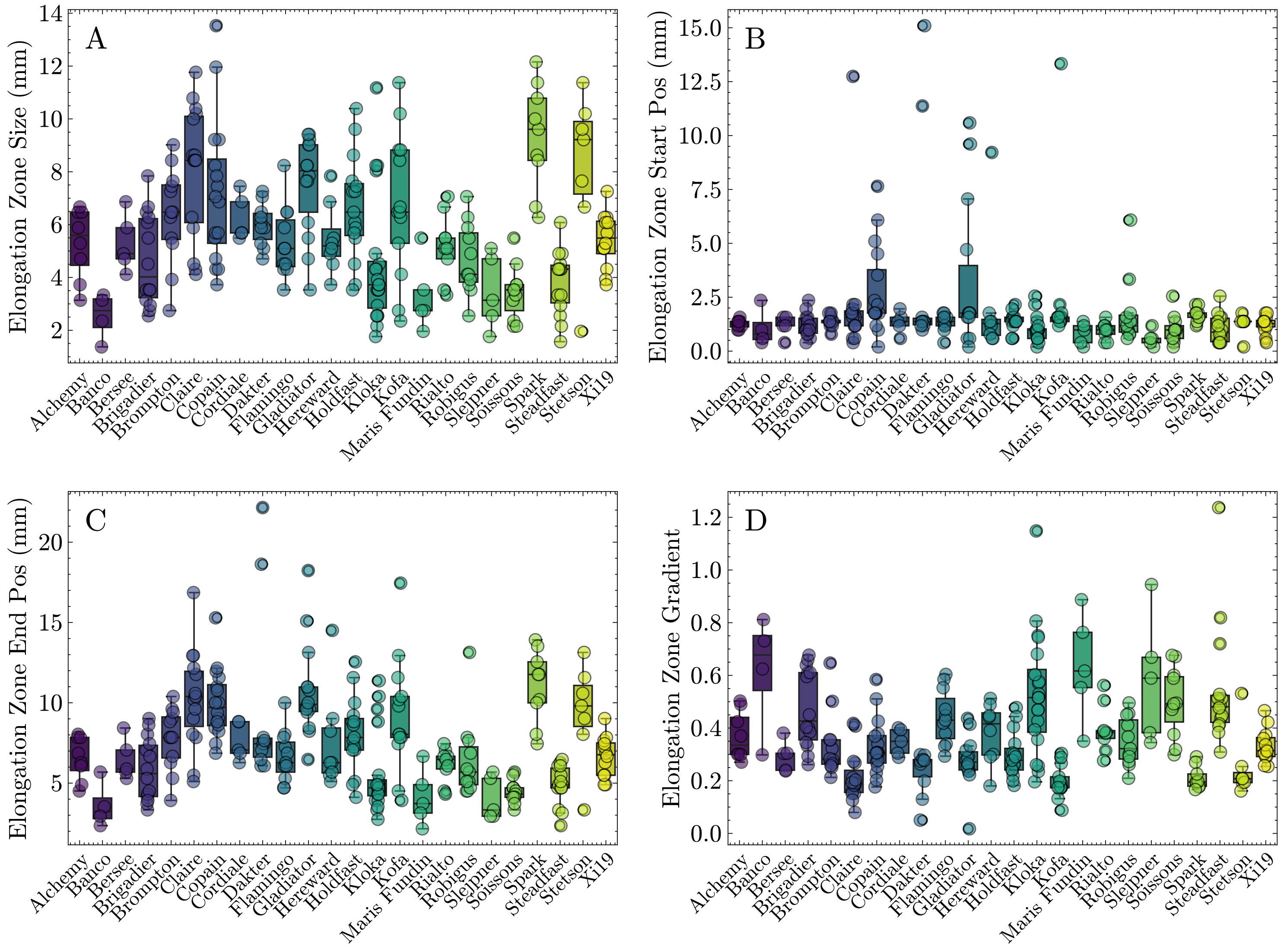

Figure 6: **Root hair elongation zone traits.** Boxplots illustrate spread of data for each cultivar and each trait. The horizontal lines within each boxplot illustrates the median, whiskers represent the data range, and data from an individual image is displayed as a dot.

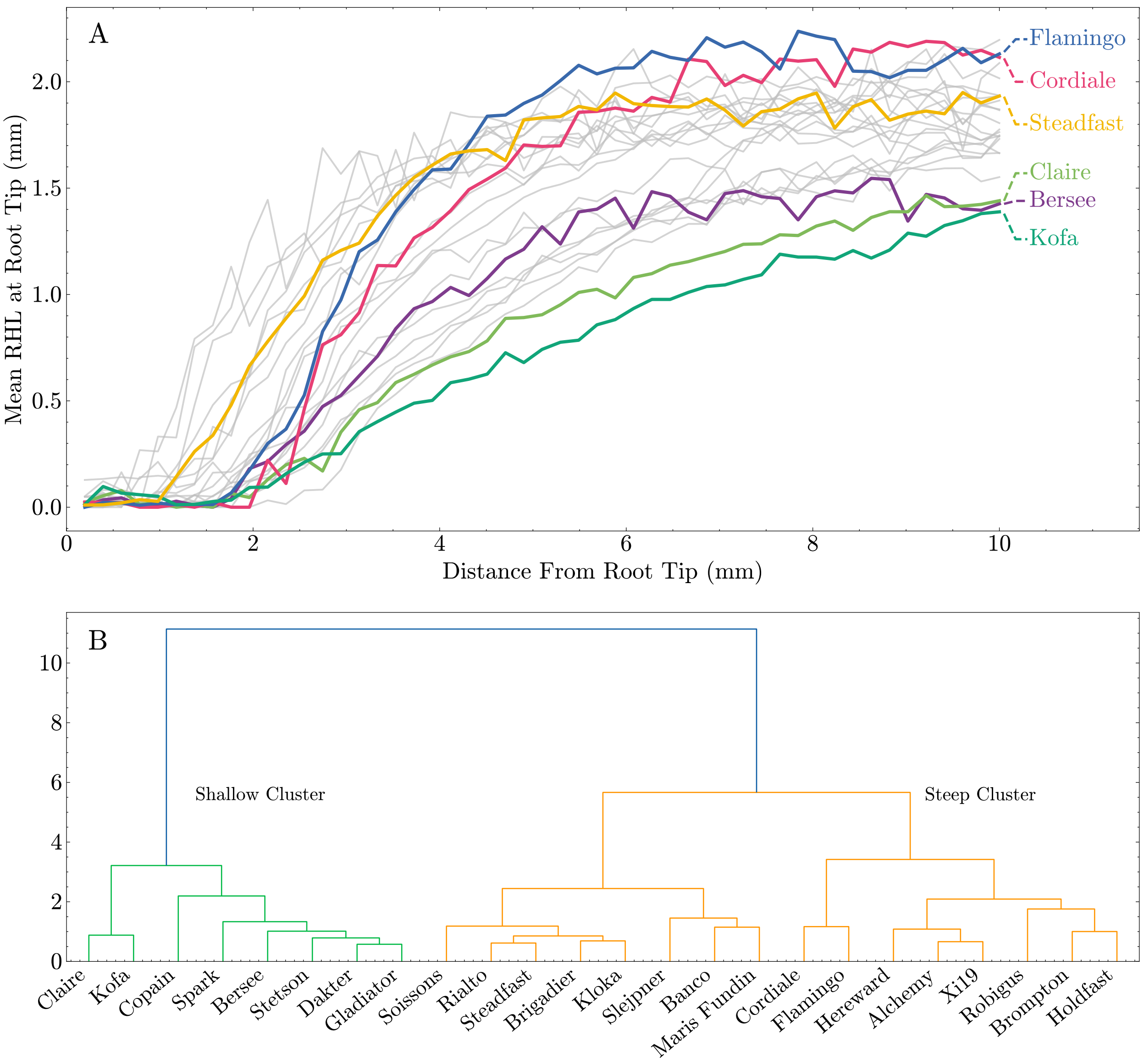

Figure 7: **Variation in root hair profile around the root tip (1cm) across the 24 wheat cultivars phenotyped using pyRootHair.** A) Mean root hair length (RHL) profile, highlighting cultivars exhibiting extreme variation in RHL within the region displayed. B) Unsupervised agglomerative clustering of root hair profiles highlights two main clusters of root hair profiles around the root tip: 'Shallow' (Green cluster) and 'Steep (Orange cluster)'. See Supplementary Figure 1A for examples of cultivars with 'shallow' and 'steep' profiles.

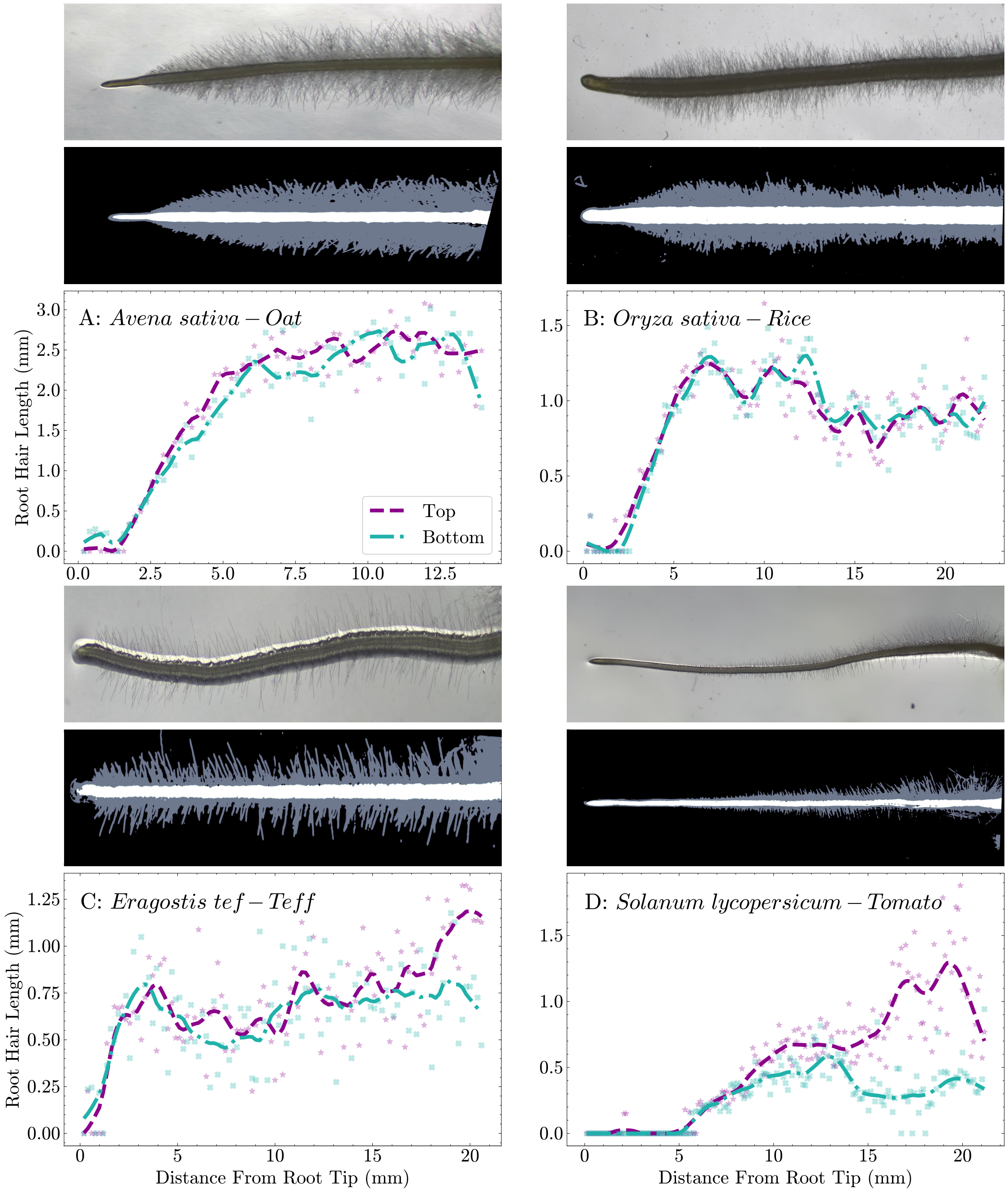

Figure 8: **pyRootHair deployed on root images captured from different plant species.** A) oat, B) rice, C) teff, D) tomato. For each panel: raw images on top, predicted segmentation masks in the middle, automated plots of root hair length (RHL) on the bottom. Raw images and segmentation masks not to scale. Top = top segment of root hairs, Bottom = bottom segment of root hairs.

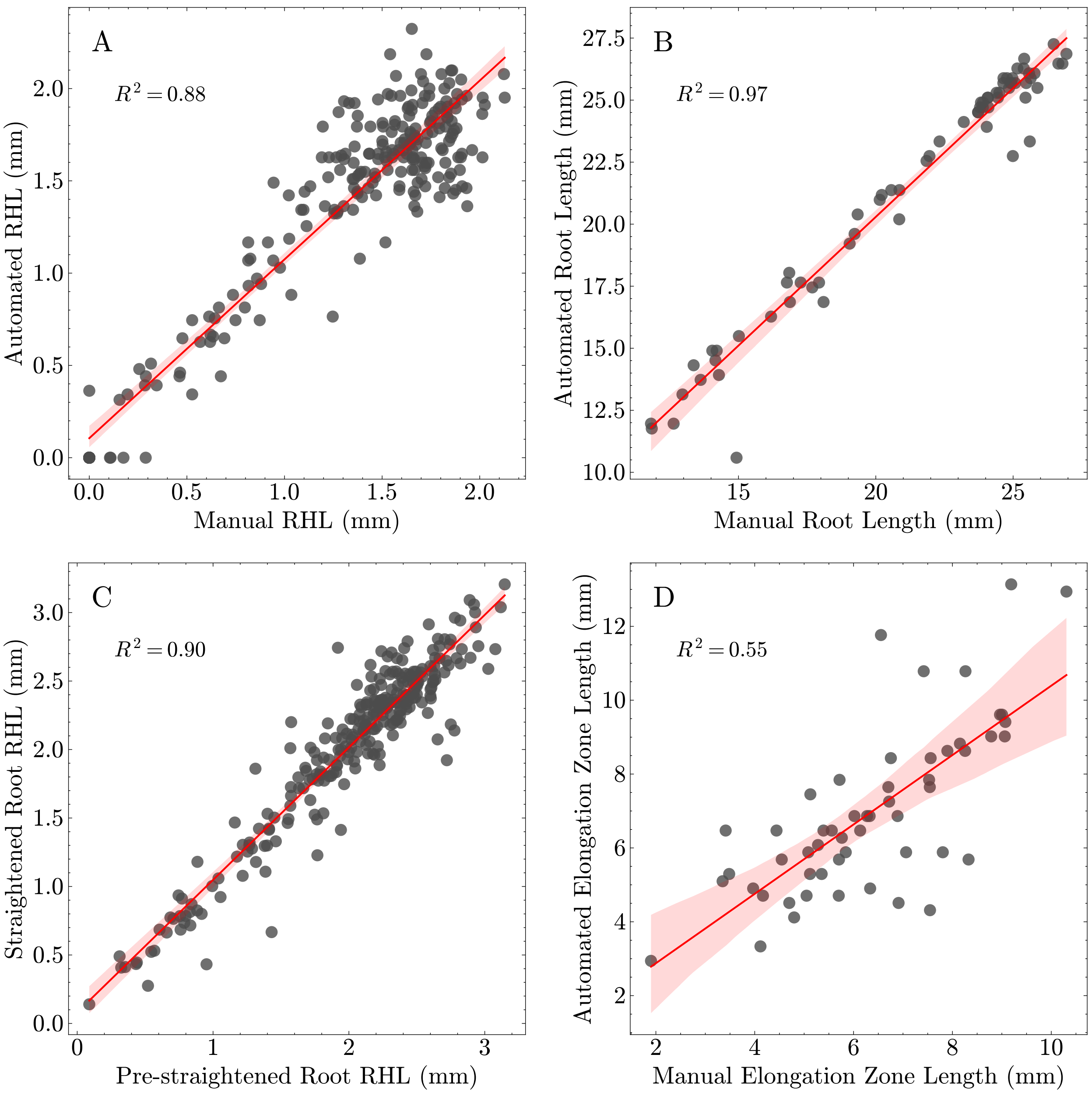

Figure 9: **Correlation (R^2) between manually measured traits and automated trait extraction using pyRootHair.** A) Root Hair Length (RHL). B) Root Length. C) RHL in pre-straightened and straightened images. D) Elongation Zone Length. Regression line with 99% confidence interval illustrated in red.

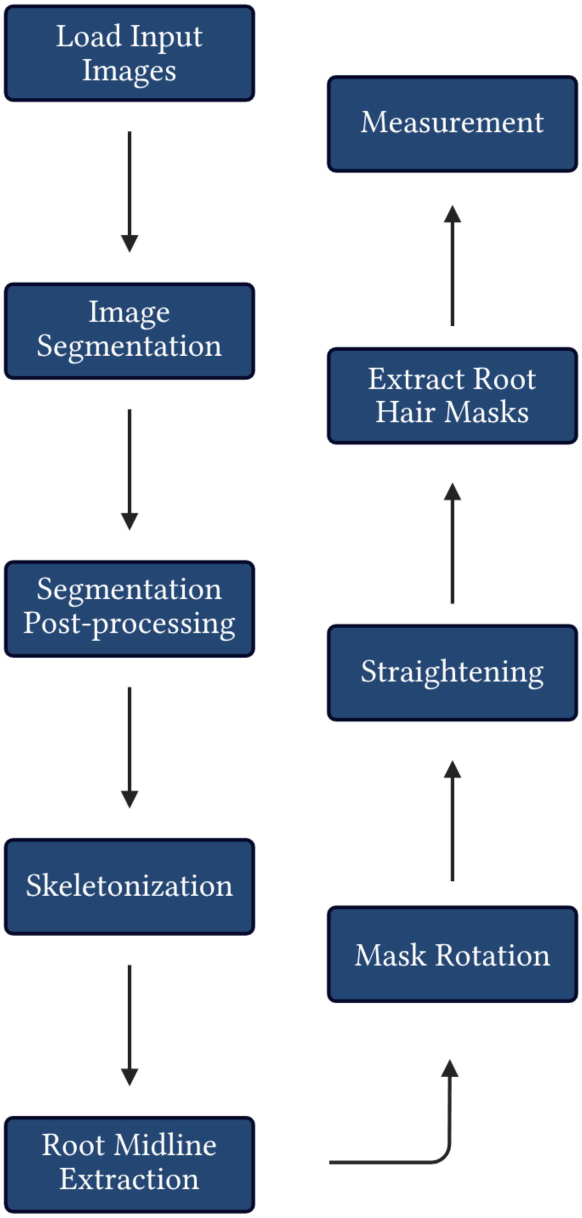

Figure 10: **Simple overview of pyRootHair processing methodology.**

13 Supplementary Figures

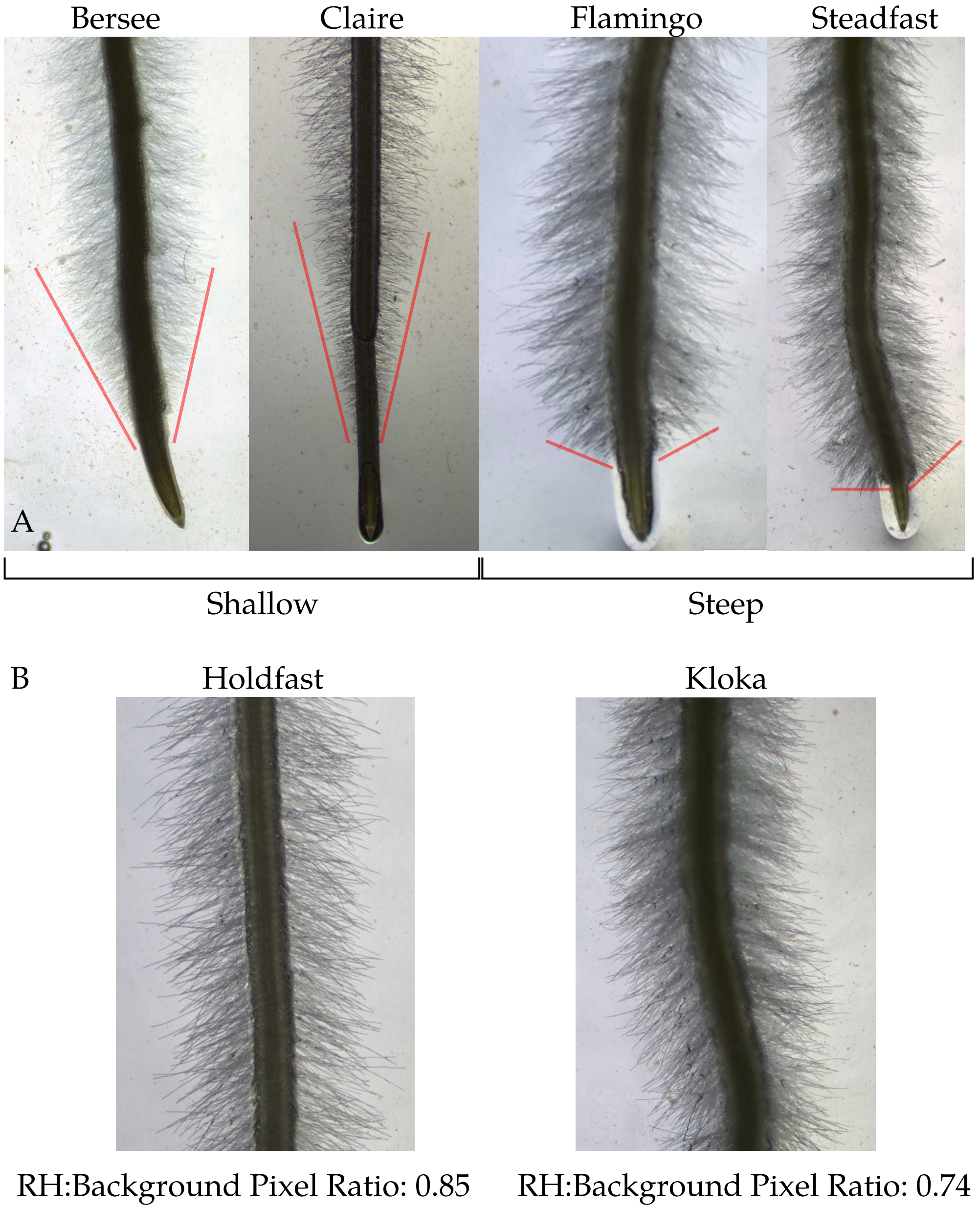

Supplementary Figure 1: **Highlighting variation in root hairs around root tips.** A) Representative images of root tips of wheat cultivars with 'shallow' (Bersee, Claire) and 'steep' (Flamingo, Steadfast) root hair profiles as identified in Figure 7. Red lines illustrate the difference in gradient of the root hair profile at root tip. B) A visual representation between cultivars with a higher (Holdfast) and lower (Kloka) RH:Background Pixel Ratio. Low values indicate increased root hair area (RHA), while high values indicate low RHA.

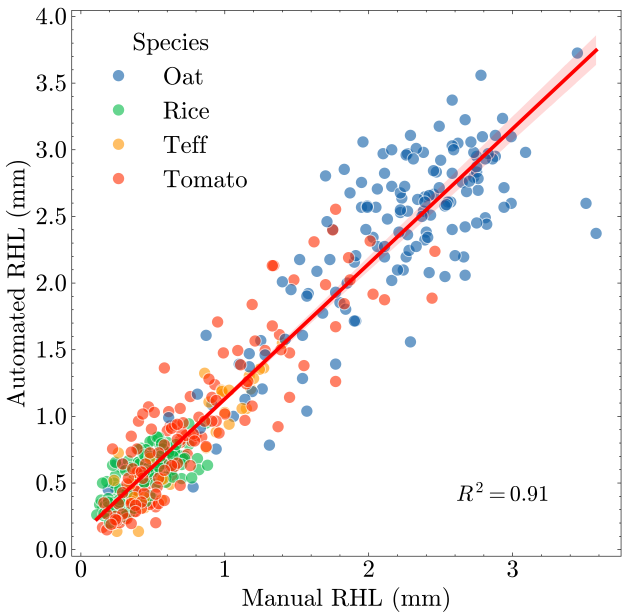

Supplementary Figure 2: **Validation of root hair length (RHL) measurements in oat, rice, teff and tomato.** Manual RHL measurements were performed in FIJI across three different images for all four species. Regression line with 99% confidence interval illustrated in red.

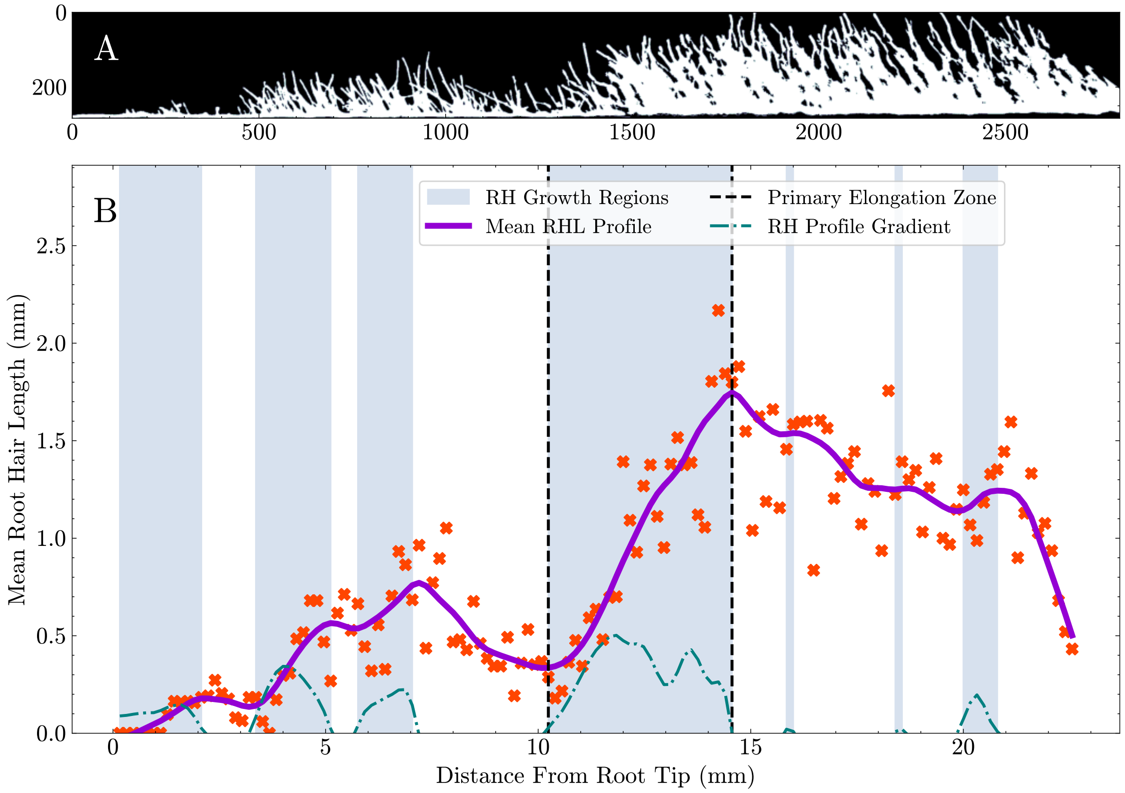

Supplementary Figure 3: **Automated root hair elongation zone calculation.** A) Example segmentation mask of a root hair section. B) Example of a plot automatically generated by pyRootHair with --plot-summary, corresponding to data extracted from the mask in A). Red crosses illustrate root hair length (RHL) from sliding window along root hair mask. Purple line illustrates mean root hair profile derived from a regression line. Dashed green line illustrates the gradient of the root hair profile (purple line). Regions where $y>0$ for the dashed green line indicate regions of positive root hair growth, highlighted by the grey rectangles. The largest region is automatically determined as the root hair elongation zone, bound by the vertical dashed black lines. RH: Root Hair

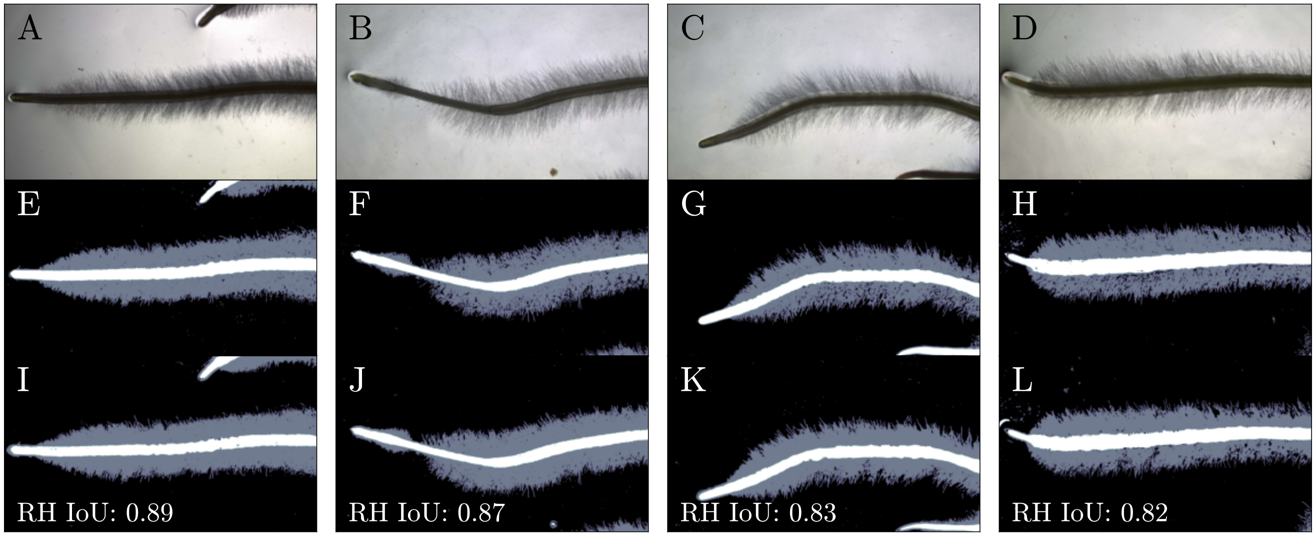

Supplementary Figure 4: **Validation of Convolutional Neural Network (CNN) segmentation on four selected images**. A-D) Raw Images, E-H) Manually annotated segmentation masks of the above images using ilastik (Berg et al. 2019), I-L) Predicted segmentation masks generated from the CNN. Intersection over union (IoU) scores for the root hair masks are shown at the bottom. IoU scores illustrate proportion of overlapping pixels in the manually annotated (E-H, grey) and the predicted root hair masks (I-L, grey), divided by the total area of root hair masks minus the overlapping region (intersection).

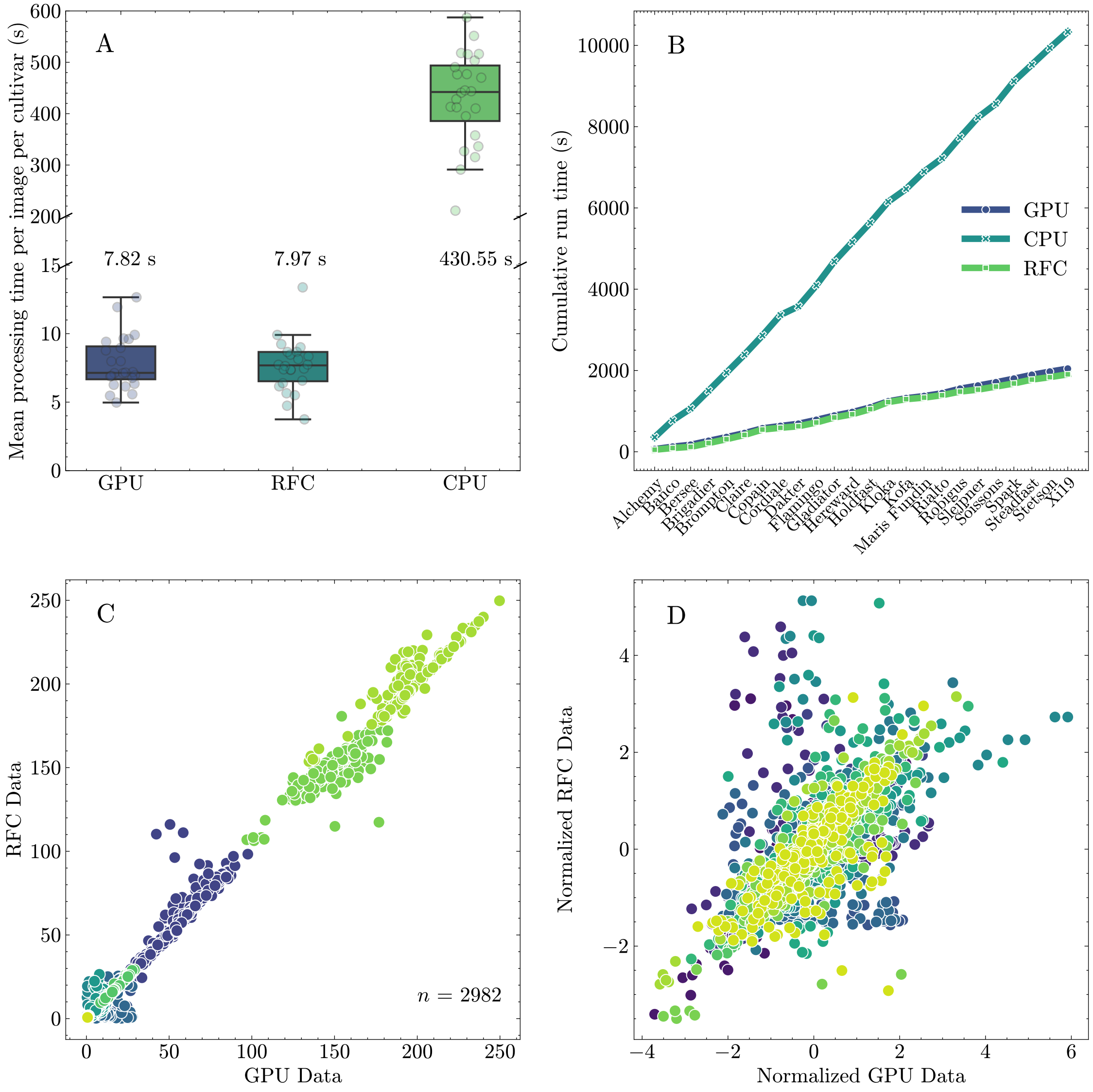

Supplementary Figure 5: **Comparing processing times between different pyRootHair pipeline configurations.** A) Boxplot illustrating the mean processing duration in seconds (including inference) for each image within each cultivar input folder. Mean runtime per image annotated above/below each box. CPU pipeline utilized the same CNN deployed in the main (GPU) pipeline to perform inference without a GPU. B) Cumulative run time for all 252 images used in this study across all three pipeline options. (C) Summary data and (D) mean normalized summary data calculated for all images using the default pipeline (GPU) and the random forest classifier (RFC) pipeline. The GPU pipeline was run using an Nvidia L40S GPU with 8 GB VRAM. The RFC pipeline was run on an HPC compute node with 20 GB RAM using a RFC model trained on a single image. CNN: convolutional neural network. GPU: Graphical Processing Unit. RFC: Random Forest Classifier. CPU: Central Processing Unit, HPC: High Performance Computer

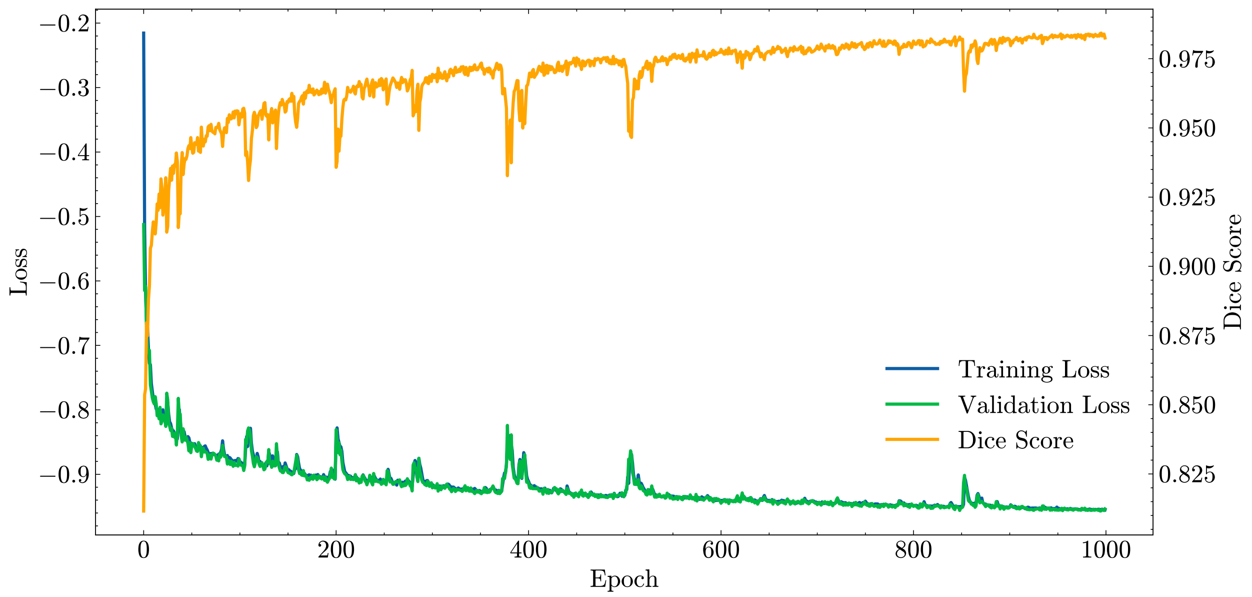

Supplementary Figure 6: **nnUNet Model Training Metrics.** Illustration of training loss, validation loss and dice scores for each epoch (n=1000). Mean validation dice score: 0.98 across 83 training instances.

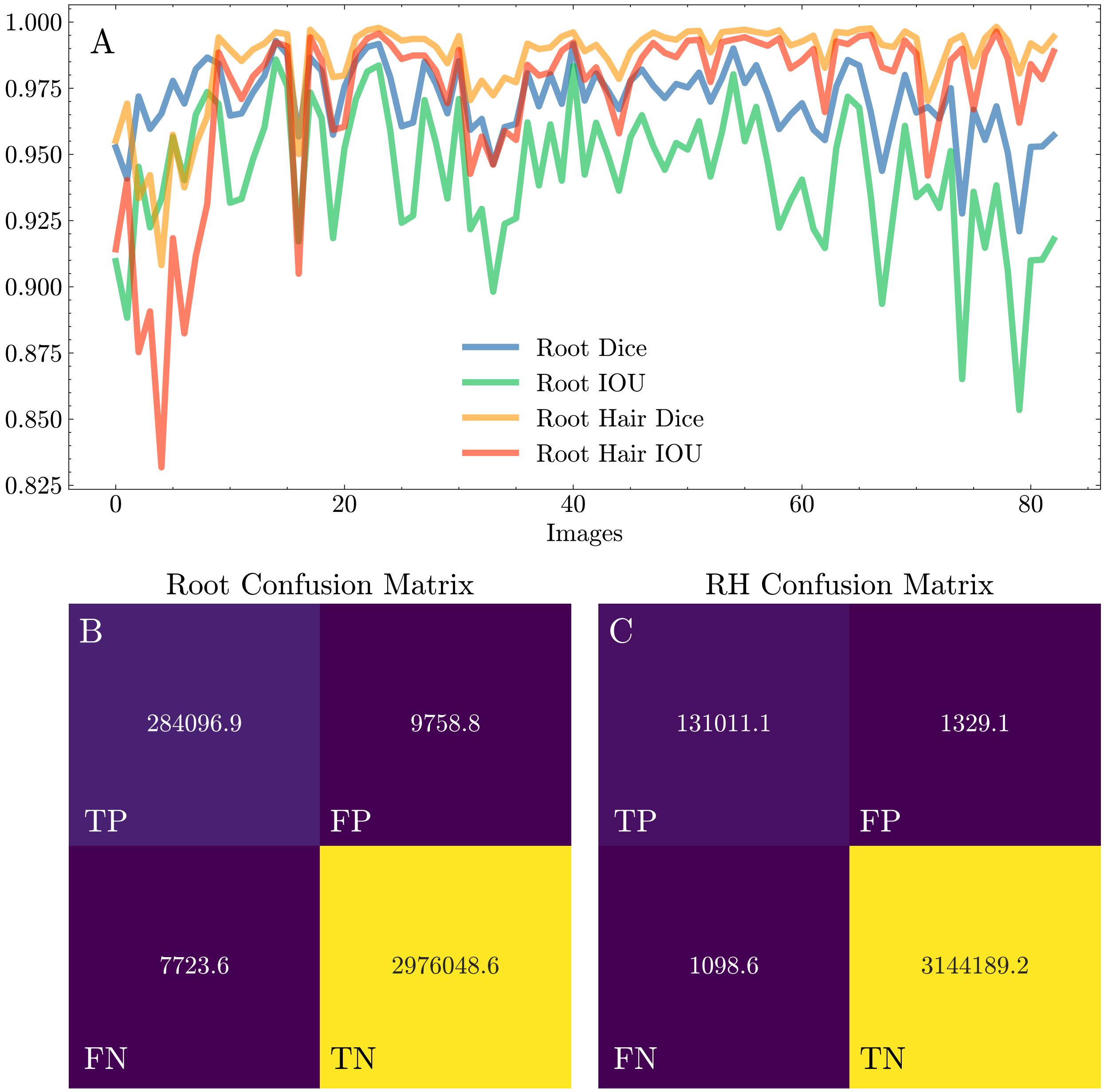

Supplementary Figure 7: **nnUNet Model Validation Metrics.** A) Dice and IoU scores for root and root hair masks for each individual training image ($n = 83$) during model validation. Confusion matrices for B) root and C) root hair segmentation masks during validation. Values within each cell represent the mean number of pixels across all training images assigned to the particular class. TP: True Positive, TN: True Negative, FP: False Positive, FN: False Negative, IoU: Intersection Over Union

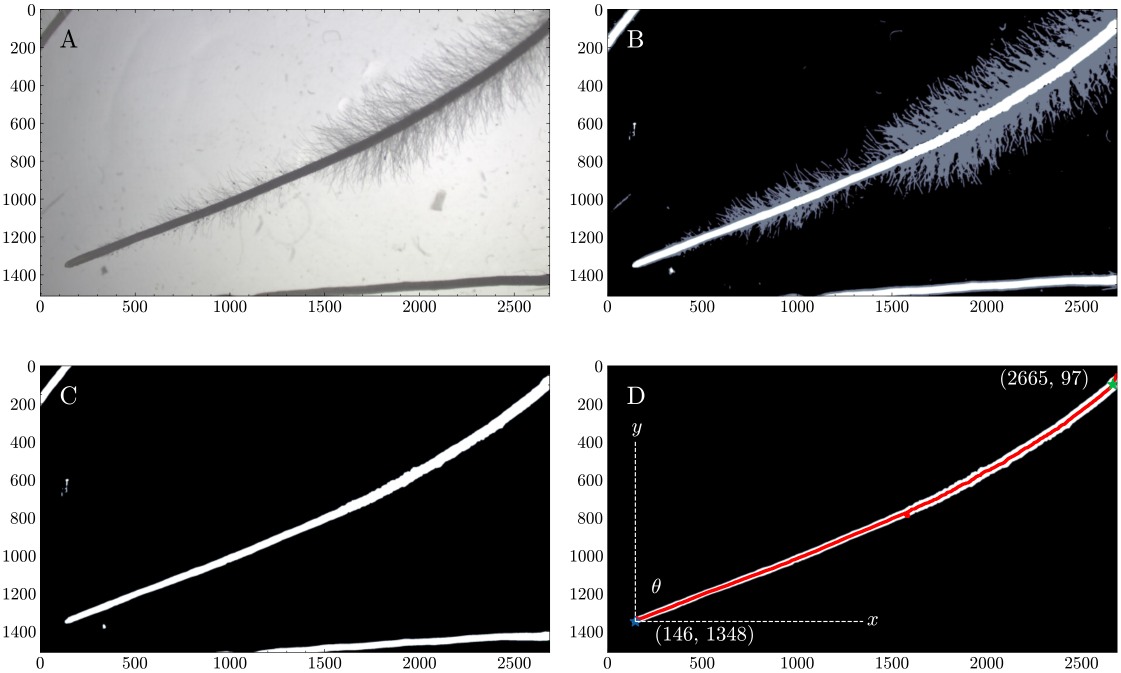

Supplementary Figure 8: **Segmentation, root extraction and midline approximation** A) Example of a raw input image into pyRootHair. B) Segmentation mask of the input image. C) Segmentation mask of all root objects. D) Removal of non-primary root objects within the mask. The root mask is skeletonized, and a cubic spline is mapped to the root skeleton. A sliding window is used to approximate the root midline (in red) by calculating the median co-ordinates of the cubic spline down the root. The root endpoints (in parentheses) are retrieved from the approximated midline and used to calculate the angle (θ) of the root relative to the vertical (y-axis). Here, the angle θ is calculated by $\arctan\left(\frac{|2665-146|}{|97-1348)|}\right)^{\circ} = 63.6^{\circ}$.

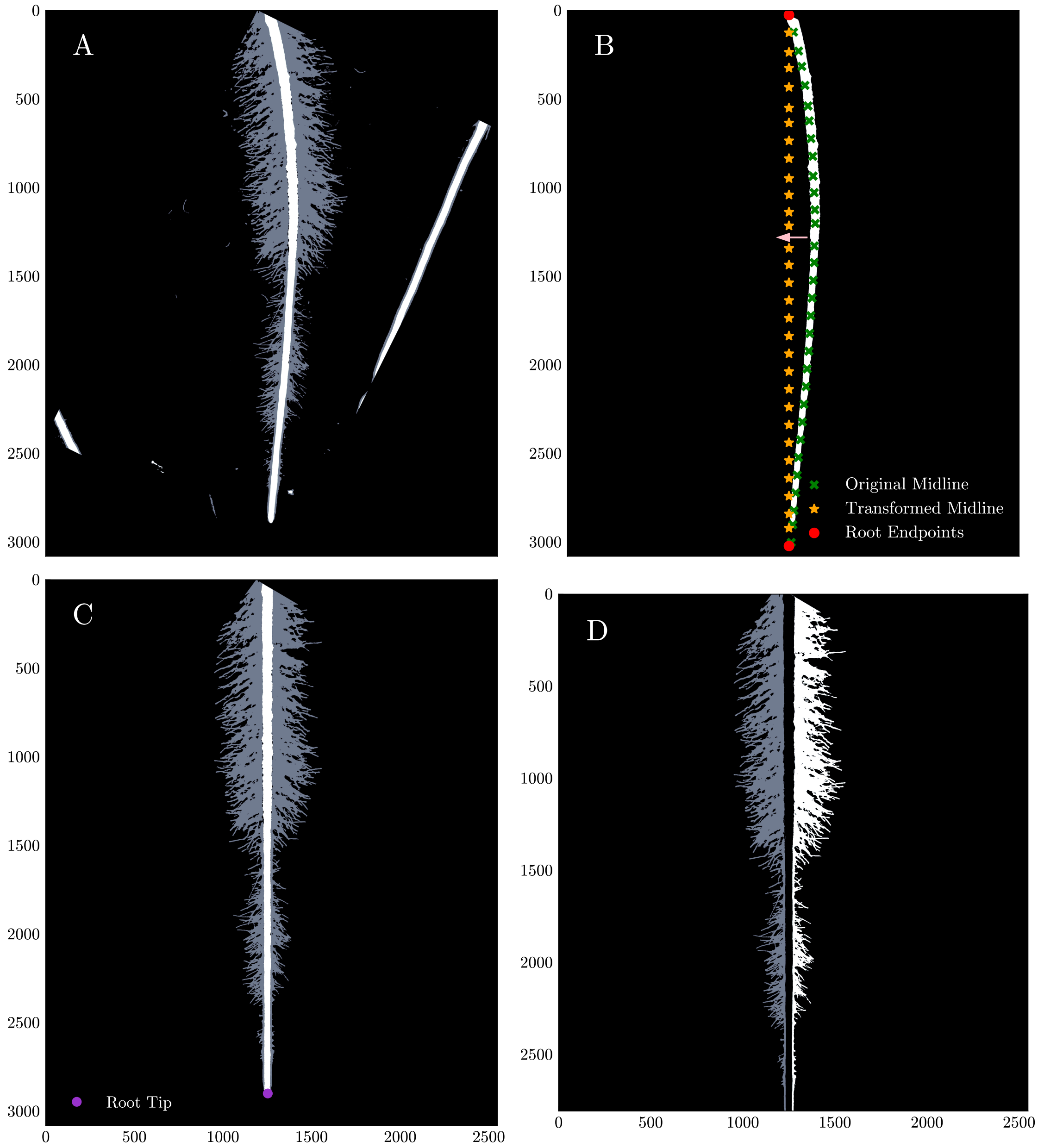

Supplementary Figure 9: **Root straightening** A) Segmentation mask of input image, rotated by angle θ such that the root is oriented downwards. B) Root midline is approximated as previously described (Supplementary Figure 8), illustrated by green crosses. Orange stars illustrate midline of new, straight root, which starts and ends at the same endpoints of the original root (red circles). Euclidean distances between midline points are preserved from the original root midline to the new root midline. The direction of image transformation is shown by the pink arrow. C) Straightened segmentation mask via piecewise affine transformation from $scikit-image$ (Van Der Walt et al. 2014) and the root tip is located (purple circle). D) Removal of root mask, preserving the two root hair segments on either side of the root. Root hair masks are cropped such that an equal length of root hair segment is processed downstream.

References

Aufrecht, Jayde A., Jennifer M. Ryan, Sahar Hasim, David P. Allison, Andreas Nebenführ, Mitchel J. Doktycz, and Scott T. Retterer (Aug. 15, 2017). "Imaging the Root Hair Morphology of Arabidopsis Seedlings in a Two-layer Microfluidic Platform". In: *Journal of Visualized Experiments* 126, p. 55971. ISSN: 1940-087X. DOI: 10.3791/55971-v. URL: https://app.jove.com/v/55971 (visited on 01/22/2025).

Bahmani, Ramin, Dong G. Kim, Jin A. Kim, and Seongbin Hwang (Nov. 23, 2016). "The Density and Length of Root Hairs Are Enhanced in Response to Cadmium and Arsenic by Modulating Gene Expressions Involved in Fate Determination and Morphogenesis of Root Hairs in Arabidopsis". In: *Frontiers in Plant Science* 7. ISSN: 1664-462X. DOI: 10.3389/fpls.2016.01763. URL: http://journal.frontiersin.org/article/10.3389/fpls.2016.01763/full (visited on 01/22/2025).

Berg, Stuart, Dominik Kutra, Thorben Kroeger, Christoph N. Straehle, Bernhard X. Kausler, Carsten Haubold, Martin Schiegg, Janez Ales, Thorsten Beier, Markus Rudy, Kemal Eren, Jaime I Cervantes, Buote Xu, Fynn Beuttenmueller, Adrian Wolny, Chong Zhang, Ullrich Koethe, Fred A. Hamprecht, and Anna Kreshuk (Dec. 2019). "ilastik: interactive machine learning for (bio)image analysis". In: *Nature Methods* 16.12, pp. 1226–1232. ISSN: 1548-7091, 1548-7105. DOI: 10.1038/s41592-019-0582-9. URL: https://www.nature.com/articles/s41592-019-0582-9 (visited on 02/17/2025).

Brueggeman, Justin M, Ian A Windham, and Andreas Nebenführ (Sept. 12, 2022). "Nuclear movement in growing Arabidopsis root hairs involves both actin filaments and microtubules". In: *Journal of Experimental Botany* 73.16. Ed. by Anja Geitmann, pp. 5388–5399. ISSN: 0022-0957, 1460-2431. DOI: 10.1093/jxb/erac207. URL: https://academic.oup.com/jxb/article/73/16/5388/6584498 (visited on 01/22/2025).

Choi, Hee-Seung and Hyung-Taeg Cho (Aug. 1, 2019). "Root hairs enhance Arabidopsis seedling survival upon soil disruption". In: *Scientific Reports* 9.1, p. 11181. ISSN: 2045-2322. DOI: 10.1038/s41598-019-47733-0. URL: https://www.nature.com/articles/s41598-019-47733-0 (visited on 01/22/2025).

Daly, Keith R., Samuel D. Keyes, Shakil Masum, and Tiina Roose (Feb. 2016). "Image-based modelling of nutrient movement in and around the rhizosphere". In: *Journal of Experimental Botany* 67.4, pp. 1059–1070. ISSN: 0022-0957, 1460-2431. DOI: 10.1093/jxb/erv544. URL: https://academic.oup.com/jxb/article-lookup/doi/10.1093/jxb/erv544 (visited on 01/22/2025).

De Baets, Sarah, Thomas D. G. Denbigh, Kevin M. Smyth, Bethany M. Eldridge, Laura Weldon, Benjamin Higgins, Antoni Matyjaszkiewicz, Jeroen Meersmans, Emily R. Larson, Isaac V. Chenchiah, Tanniemola B. Liverpool, Timothy A. Quine, and Claire S. Grierson (Apr. 3, 2020). "Micro-scale interactions between Arabidopsis root hairs and soil particles influence soil erosion". In: *Communications Biology* 3.1, p. 164. ISSN: 2399-3642. DOI: 10.1038/s42003-020-0886-4. URL: https://www.nature.com/articles/s42003-020-0886-4 (visited on 05/15/2023).

Dolan, Liam (Aug. 2017). "Root hair development in grasses and cereals (Poaceae)". In: *Current Opinion in Genetics & Development* 45, pp. 76–81. ISSN: 0959437X. DOI: 10.1016/j.gde.2017.03.009. URL: https://linkinghub.elsevier.com/retrieve/pii/S0959437X16301319 (visited on 01/17/2023).

Gahoonia, Tara S. and Niels E. Nielsen (May 2004). "Barley genotypes with long root hairs sustain high grain yields in low-P field". In: *Plant and Soil* 262.1, pp. 55–62. ISSN: 0032-079X. DOI: 10.1023/B:PLSO.

0000037020.58002.ac. URL: http://link.springer.com/10.1023/B:PLSO.0000037020.58002.ac (visited on 04/09/2024).

Grierson, Claire, Erik Nielsen, Tijs Ketelaarc, and John Schiefelbein (Jan. 2014). “Root Hairs”. In: *The Arabidopsis Book* 12, e0172. ISSN: 1543-8120. DOI: 10.1199/tab.0172. URL: http://www.bioone.org/doi/10.1199/tab.0172 (visited on 11/01/2023).

Guichard, Marjorie, Jean-Marc Allain, Michele Wolfe Bianchi, and Jean-Marie Frachisse (Dec. 2019). “Root Hair Sizer: an algorithm for high throughput recovery of different root hair and root developmental parameters”. In: *Plant Methods* 15.1, p. 104. ISSN: 1746-4811. DOI: 10.1186/s13007-019-0483-z. URL: https://plantmethods.biomedcentral.com/articles/10.1186/s13007-019-0483-z (visited on 11/20/2023).

Haling, Rebecca E., Lawrie K. Brown, A. Glyn Bengough, Iain M. Young, Paul D. Hallett, Philip J. White, and Timothy S. George (Sept. 2013). “Root hairs improve root penetration, root–soil contact, and phosphorus acquisition in soils of different strength”. In: *Journal of Experimental Botany* 64.12, pp. 3711–3721. ISSN: 1460-2431, 0022-0957. DOI: 10.1093/jxb/ert200. URL: https://academic.oup.com/jxb/article-lookup/doi/10.1093/jxb/ert200 (visited on 11/17/2022).

Huang, Feng, Zhaoyan Chen, Dejie Du, Panfeng Guan, Lingling Chai, Weilong Guo, Zhaorong Hu, Mingming Xin, Huiru Peng, Yingyin Yao, and Zhongfu Ni (Dec. 2020). “Genome-wide linkage mapping of QTL for root hair length in a Chinese common wheat population”. In: *The Crop Journal* 8.6, pp. 1049–1056. ISSN: 22145141. DOI: 10.1016/j.cj.2020.02.007. URL: https://linkinghub.elsevier.com/retrieve/pii/S2214514120300489 (visited on 10/26/2022).

Isensee, Fabian, Paul F. Jaeger, Simon A. A. Kohl, Jens Petersen, and Klaus H. Maier-Hein (Feb. 2021). “nnU-Net: a self-configuring method for deep learning-based biomedical image segmentation”. In: *Nature Methods* 18.2, pp. 203–211. ISSN: 1548-7091, 1548-7105. DOI: 10.1038/s41592-020-01008-z. URL: https://www.nature.com/articles/s41592-020-01008-z (visited on 02/17/2025).

Isensee, Fabian, Tassilo Wald, Constantin Ulrich, Michael Baumgartner, Saikat Roy, Klaus Maier-Hein, and Paul F. Jaeger (2024). *nnU-Net Revisited: A Call for Rigorous Validation in 3D Medical Image Segmentation.* Version Number: 2. DOI: 10.48550/ARXIV.2404.09556. URL: https://arxiv.org/abs/2404.09556 (visited on 02/17/2025).

Keyes, Samuel D., Keith R. Daly, Neil J. Gostling, Davey L. Jones, Peter Talboys, Bernd R. Pinzer, Richard Boardman, Ian Sinclair, Alan Marchant, and Tiina Roose (June 2013). “High resolution synchrotron imaging of wheat root hairs growing in soil and image based modelling of phosphate uptake”. In: *New Phytologist* 198.4, pp. 1023–1029. ISSN: 0028-646X, 1469-8137. DOI: 10.1111/nph.12294. URL: https://onlinelibrary.wiley.com/doi/10.1111/nph.12294 (visited on 10/24/2022).

Liu, Chun-Yan, Fei Zhang, De-Jian Zhang, Ak Srivastava, Qiang-Sheng Wu, and Ying-Ning Zou (Jan. 31, 2018). “Mycorrhiza stimulates root-hair growth and IAA synthesis and transport in trifoliate orange under drought stress”. In: *Scientific Reports* 8.1, p. 1978. ISSN: 2045-2322. DOI: 10.1038/s41598-018-20456-4. URL: https://www.nature.com/articles/s41598-018-20456-4 (visited on 01/21/2025).

Liu, Lin, Lu-Guang Jiang, Jin-Hong Luo, Ai-Ai Xia, Li-Qun Chen, and Yan He (Dec. 2021). “Genome-wide association study reveals the genetic architecture of root hair length in maize”. In: *BMC Genomics* 22.1, p. 664. ISSN: 1471-2164. DOI: 10.1186/s12864-021-07961-z. URL: https://bmcgenomics.biomedcentral.com/articles/10.1186/s12864-021-07961-z (visited on 01/21/2025).

Lu, Wei, Xiaochan Wang, and Weidong Jia (Nov. 2022). “Root hair image processing based on deep learning and prior knowledge”. In: *Computers and Electronics in Agriculture* 202, p. 107397. ISSN: 01681699. DOI: 10.1016/j.compag.2022.107397. URL: https://linkinghub.elsevier.com/retrieve/pii/S0168169922007050 (visited on 02/06/2025).

Mackay, Ian J, Pauline Bansept-Basler, Toby Barber, Alison R Bentley, James Cockram, Nick Gosman, Andy J Greenland, Richard Horsnell, Rhian Howells, Donal M O’Sullivan, Gemma A Rose, and Phil J Howell (Sept. 1, 2014). “An Eight-Parent Multiparent Advanced Generation Inter-Cross Population for Winter-Sown Wheat: Creation, Properties, and Validation”. In: *G3 Genes—Genomes—Genetics* 4.9, pp. 1603–1610. ISSN: 2160-1836. DOI: 10.1534/g3.114.012963. URL: https://academic.oup.com/g3journal/article/4/9/1603/6025902 (visited on 05/15/2025).

Maqbool, Saman, Fatima Saeed, Ali Raza, Awais Rasheed, and Zhonghu He (Aug. 29, 2022). “Association of Root Hair Length and Density with Yield-Related Traits and Expression Patterns of TaRSL4 Underpinning Root Hair Length in Spring Wheat”. In: *Plants* 11.17, p. 2235. ISSN: 2223-7747. DOI: 10.3390/plants11172235. URL: https://www.mdpi.com/2223-7747/11/17/2235 (visited on 10/24/2022).

Pacheco, Javier Martínez, Philippe Ranocha, Luciana Kasulin, Corina M. Fusari, Lucas Servi, Ariel. A. Aptekmann, Victoria Berdion Gabarain, Juan Manuel Peralta, Cecilia Borassi, Eliana Marzol, Diana Rosa Rodríguez-Garcia, Yossmayer del Carmen Rondón Guerrero, Mariana Carignani Sardoy, Lucía Ferrero, Javier F. Botto, Claudio Meneses, Federico Ariel, Alejandro D. Nadra, Ezequiel Petrillo, Christophe Dunand, and José M. Estevez (Mar. 14, 2022). “Apoplastic class III peroxidases PRX62 and PRX69 promote Arabidopsis root hair growth at low temperature”. In: *Nature Communications* 13.1, p. 1310. ISSN: 2041-1723. DOI: 10.1038/s41467-022-28833-4. URL: https://www.nature.com/articles/s41467-022-28833-4 (visited on 02/08/2023).

Percival-Alwyn, Lawrence, Ian Barnes, Matthew D. Clark, James Cockram, Michael P. Coffey, Susan Jones, Paul J. Kersey, Catherine A. Kidner, Carolin Kosiol, Bingjie Li, William A. Marsh, Ji Zhou, Mario Caccamo, and Iain Milne (Dec. 5, 2024). “UKCropDiversity-HPC: A collaborative high-performance computing resource approach for sustainable agriculture and biodiversity conservation”. In: *PLANTS, PEOPLE, PLANET*, ppp3.10607. ISSN: 2572-2611, 2572-2611. DOI: 10.1002/ppp3.10607. URL: https://nph.onlinelibrary.wiley.com/doi/10.1002/ppp3.10607 (visited on 02/17/2025).

Pereira, David, Thomas Alline, Léa Cascaro, Emilie Lin, and Atef Asnacios (June 14, 2024). “Mechanical resistance of the environment affects root hair growth and nucleus dynamics”. In: *Scientific Reports* 14.1, p. 13788. ISSN: 2045-2322. DOI: 10.1038/s41598-024-64423-8. URL: https://www.nature.com/articles/s41598-024-64423-8 (visited on 01/21/2025).

Pietrzyk, Peter, Neen Phan-Udom, Chartinun Chutoe, Lise Pingault, Ankita Roy, Marc Libault, Patompong Johns Saengwilai, and Alexander Bucksch (Jan. 10, 2025). “DIRT/µ: automated extraction of root hair traits using combinatorial optimization”. In: *Journal of Experimental Botany* 76.2. Ed. by Lionel Dupuy, pp. 285–298. ISSN: 0022-0957, 1460-2431. DOI: 10.1093/jxb/erae385. URL: https://academic.oup.com/jxb/article/76/2/285/7756301 (visited on 01/22/2025).

Saengwilai, Patompong, Christopher Strock, Harini Rangarajan, Joseph Chimungu, Jirawat Salungyu, and Jonathan P Lynch (Nov. 9, 2021). “Root hair phenotypes influence nitrogen acquisition in maize”. In: *Annals of Botany* 128.7, pp. 849–858. ISSN: 0305-7364, 1095-8290. DOI: 10.1093/aob/mcab104. URL: https://academic.oup.com/aob/article/128/7/849/6342804 (visited on 04/11/2023).

Schindelin, Johannes, Ignacio Arganda-Carreras, Erwin Frise, Verena Kaynig, Mark Longair, Tobias Pietzsch, Stephan Preibisch, Curtis Rueden, Stephan Saalfeld, Benjamin Schmid, Jean-Yves Tinevez, Daniel James White, Volker Hartenstein, Kevin Eliceiri, Pavel Tomancak, and Albert Cardona (July 2012). "Fiji: an open-source platform for biological-image analysis". In: *Nature Methods* 9.7, pp. 676–682. ISSN: 1548-7091, 1548-7105. DOI: 10.1038/nmeth.2019. URL: https://www.nature.com/articles/nmeth.2019 (visited on 03/11/2024).

Scott, Michael F., Nick Fradgley, Alison R. Bentley, Thomas Brabbs, Fiona Corke, Keith A. Gardner, Richard Horsnell, Phil Howell, Olufunmilayo Ladejobi, Ian J. Mackay, Richard Mott, and James Cockram (Dec. 2021). "Limited haplotype diversity underlies polygenic trait architecture across 70 years of wheat breeding". In: *Genome Biology* 22.1, p. 137. ISSN: 1474-760X. DOI: 10.1186/s13059-021-02354-7. URL: https://genomebiology.biomedcentral.com/articles/10.1186/s13059-021-02354-7 (visited on 05/15/2025).

Singh, Gaurav, David Pereira, Stéphanie Baudrey, Elise Hoffmann, Michael Ryckelynck, Atef Asnacios, and Marie-Edith Chaboute (Oct. 2021). "Real-time tracking of root hair nucleus morphodynamics using a microfluidic approach". In: *The Plant Journal* 108.2, pp. 303–313. ISSN: 0960-7412, 1365-313X. DOI: 10.1111/tpj.15511. URL: https://onlinelibrary.wiley.com/doi/10.1111/tpj.15511 (visited on 01/22/2025).

Stetter, Markus G., Karl Schmid, and Uwe Ludewig (Mar. 17, 2015). "Uncovering Genes and Ploidy Involved in the High Diversity in Root Hair Density, Length and Response to Local Scarce Phosphate in Arabidopsis thaliana". In: *PLOS ONE* 10.3, e0120604. ISSN: 1932-6203. DOI: 10.1371/journal.pone.0120604. URL: https://dx.plos.org/10.1371/journal.pone.0120604 (visited on 01/24/2025).

Tsang, Ian, Jonathan A Atkinson, Stephen Rawsthorne, James Cockram, and Fiona Leigh (Sept. 27, 2024a). "Root hairs: an underexplored target for sustainable cereal crop production". In: *Journal of Experimental Botany* 75.18. Ed. by Kris Vissenberg, pp. 5484–5500. ISSN: 0022-0957, 1460-2431. DOI: 10.1093/jxb/erae275. URL: https://academic.oup.com/jxb/article/75/18/5484/7696010 (visited on 10/11/2024).

Tsang, Ian, Pauline Thomelin, Eric S. Ober, Stephen Rawsthorne, Jonathan A. Atkinson, Darren M. Wells, Lawrence Percival-Alwyn, Fiona J. Leigh, and James Cockram (Oct. 30, 2024b). "A novel root hair mutant, srh1, affects root hair elongation and reactive oxygen species levels in wheat". In: *Frontiers in Plant Science* 15. Publisher: Frontiers. ISSN: 1664-462X. DOI: 10.3389/fpls.2024.1490502. URL: https://www.frontiersin.org/journals/plant-science/articles/10.3389/fpls.2024.1490502/full (visited on 02/06/2025).

Van Der Walt, Stéfan, Johannes L. Schönberger, Juan Nunez-Iglesias, François Boulogne, Joshua D. Warner, Neil Yager, Emmanuelle Gouillart, and Tony Yu (June 19, 2014). "scikit-image: image processing in Python". In: *PeerJ* 2, e453. ISSN: 2167-8359. DOI: 10.7717/peerj.453. URL: https://peerj.com/articles/453 (visited on 09/09/2025).

Vatter, Thomas, Benjamin Neuhäuser, Markus Stetter, and Uwe Ludewig (Sept. 2015). "Regulation of length and density of Arabidopsis root hairs by ammonium and nitrate". In: *Journal of Plant Research* 128.5, pp. 839–848. ISSN: 0918-9440, 1618-0860. DOI: 10.1007/s10265-015-0733-8. URL: http://link.springer.com/10.1007/s10265-015-0733-8 (visited on 01/24/2025).

Vincent, Christopher, Diane Rowland, Chaein Na, and Bruce Schaffer (Mar. 2017). "A high-throughput method to quantify root hair area in digital images taken in situ". In: *Plant and Soil* 412.1, pp. 61–80.

ISSN: 0032-079X, 1573-5036. DOI: 10.1007/s11104-016-3016-9. URL: http://link.springer.com/10.1007/s11104-016-3016-9 (visited on 01/28/2025).

Yan, Haiting, Yue Wang, Jingrong Zhang, Xinru Cui, Jiasong Wu, Jie Zhou, Yuan Chen, Jia Lu, Ruiyang Guo, Maggie Ou, Hongxu Lai, and Zhiming Yu (2021). "Rice Root Hair Phenotypes Imaged by Cryo-SEM". In: *BIO-PROTOCOL* 11.11. ISSN: 2331-8325. DOI: 10.21769/BioProtoc.4037. URL: https://bio-protocol.org/e4037 (visited on 01/22/2025).

Zhang, Chunyan, Richard J. Simpson, Chul Min Kim, Norman Warthmann, Emmanuel Delhaize, Liam Dolan, Mary E. Byrne, Yu Wu, and Peter R. Ryan (Mar. 2018). "Do longer root hairs improve phosphorus uptake? Testing the hypothesis with transgenic Brachypodium distachyon lines overexpressing endogenous RSL genes". In: *New Phytologist* 217.4, pp. 1654–1666. ISSN: 0028-646X, 1469-8137. DOI: 10.1111/nph.14980. URL: https://nph.onlinelibrary.wiley.com/doi/10.1111/nph.14980 (visited on 11/01/2023).
