# Supplementary material for: pyRootHair: Machine learning accelerated software for high-throughput phenotyping of plant root hair traits
Source: Gigascience. 2025 Nov 13;15:giaf141. doi: 10.1093/gigascience/giaf141 (PMC12824728; doi:10.1093/gigascience/giaf141)
Supplement: giaf141_Reviewer_1_Report_Original_Submission — Wanneng Yang -- 8/10/2025 [file giaf141_reviewer_1_report_original_submission.pdf]

Reviewer Report

Title: pyRootHair: Machine Learning Accelerated Software for High-Throughput Phenotyping of Plant Root Hair Traits

Version: Original Submission **Date:** 8/10/2025

Reviewer name: Wanneng Yang

Reviewer Comments to Author:

This paper introduces an artificial intelligence-driven software named pyRootHair, which enables high-throughput automated extraction of root hair traits from plant root images, thereby facilitating rapid analysis of root hair morphological variations in various plants, including wheat. However, the following issues remain:
1ï¼‰Compared to previously published work, the contributions and innovations of this study are not sufficiently highlighted. For instance, the work by Lu, Wei, Xiaochan Wang, and Wei Jia, titled "Root hair image processing based on deep learning and prior knowledge" (Comput. Electron. Agric. 202, 2022: 107397), should be explicitly referenced to clarify the advancements presented here.
2ï¼‰ Although the study demonstrates that pyRootHair can be applied to multiple plant species, including Arabidopsis, Brachypodium, rice, and tomato, the primary validation and analysis are conducted on wheat. For other species, only segmentation results and trait extraction figures are presented, lacking detailed comparative validation with manual measurements as thoroughly as for wheat.
3ï¼‰The process of "straightening" curved roots is implemented, but the potential introduction of new errors by this procedure is not discussed.
4ï¼‰ In the trait validation section, the correlation analysis between automated and manual measurements shows strong agreement for root hair length and root length, but weaker correlation for elongation zone length. The study should provide a more in-depth discussion on the possible reasons for this lower correlation.
5ï¼‰The details of the core algorithms (CNN architecture, random forest classifier) are insufficiently described. Key aspects such as parameter selection, optimization, training procedures, and the division ratios of the training/validation/test sets are not clearly specified. Additionally, the specific strategies for data augmentation are not mentioned.
6ï¼‰ No quantitative comparisons with similar tools (e.g., in terms of speed and accuracy) are provided.

Methods

Are the methods appropriate to the aims of the study, are they well described, and are necessary controls included? Choose an item.

Conclusions

Are the conclusions adequately supported by the data shown? Choose an item.

Reporting Standards

Does the manuscript adhere to the journal's guidelines on minimum standards of reporting? Choose an item.

Choose an item.

Statistics

Are you able to assess all statistics in the manuscript, including the appropriateness of statistical tests used? Choose an item.

Quality of Written English

Please indicate the quality of language in the manuscript: Choose an item.

Declaration of Competing Interests

Please complete a declaration of competing interests, considering the following questions:

- Have you in the past five years received reimbursements, fees, funding, or salary from an organisation that may in any way gain or lose financially from the publication of this manuscript, either now or in the future?
- Do you hold any stocks or shares in an organisation that may in any way gain or lose financially from the publication of this manuscript, either now or in the future?
- Do you hold or are you currently applying for any patents relating to the content of the manuscript?
- Have you received reimbursements, fees, funding, or salary from an organization that holds or has applied for patents relating to the content of the manuscript?
- Do you have any other financial competing interests?
- Do you have any non-financial competing interests in relation to this paper?

If you can answer no to all of the above, write 'I declare that I have no competing interests' below. If your reply is yes to any, please give details below.

I declare that I have no competing interests.

I agree to the open peer review policy of the journal. I understand that my name will be included on my report to the authors and, if the manuscript is accepted for publication, my named report including any attachments I upload will be posted on the website along with the authors' responses. I agree for my report to be made available under an Open Access Creative Commons CC-BY license (http://creativecommons.org/licenses/by/4.0/). I understand that any comments which I do not wish to be included in my named report can be included as confidential comments to the editors, which will not be published.

Choose an item.

To further support our reviewers, we have joined with Publons, where you can gain additional credit to further highlight your hard work (see: https://publons.com/journal/530/gigascience). On publication of this paper, your review will be automatically added to Publons, you can then choose whether or not to claim your Publons credit. I understand this statement.

Yes Choose an item.
