# Supplementary material for: pyRootHair: Machine learning accelerated software for high-throughput phenotyping of plant root hair traits
Source: Gigascience. 2025 Nov 13;15:giaf141. doi: 10.1093/gigascience/giaf141 (PMC12824728; doi:10.1093/gigascience/giaf141)
Supplement: giaf141_Reviewer_2_Report_Original_Submission — Nicolas Gaggion -- 8/20/2025 [file giaf141_reviewer_2_report_original_submission.pdf]

Reviewer Report

Title: pyRootHair: Machine Learning Accelerated Software for High-Throughput Phenotyping of Plant Root Hair Traits

Version: Original Submission **Date:** 8/20/2025

Reviewer name: Nicolas Gaggion

Reviewer Comments to Author:

The manuscript "pyRootHair: Machine Learning Accelerated Software for High-Throughput Phenotyping of Plant Root Hair Traits" presents a valuable tool for plant phenotyping in microscopy images. As it stands, my recommendation is to accept after minor revisions.
I found the GitHub repository provides detailed instructions and was straightforward to install and run. The whole process took only a few minutes to execute, which speaks well to the software's accessibility. To further enhance the clarity, precision, and accessibility of the manuscript, I have several comments. On the random forest classifier training process, the manuscript states "For this comparison, the RFC was trained on a single input image, and used to perform inference on all subsequent images." However, the repository documentation indicates: "To train a random forest model, you will need to train the model on a single representative example of an image, and a corresponding binary mask of the image." The repository further notes that "You will need to ensure that all the images are relatively consistent in terms of lighting, appearance, root hair morphology, and have the same input dimensions. Should your images vary for these traits, you will need to train separate random forest models for different batches of images."
The manuscript needs clarification on this training process. Please specify whether users are expected to manually segment one of their own images, use the nnUNet model to generate binary segmentation and refine it using annotation tools (such as ilastik), or select one of the images from ones provided by the authors of the manuscript that best matches their new data.
Regarding nnUNet performance, I support the decision not to compare with other models, as nnUNet represents state-of-the-art performance and enables easy training for non-expert users. However, I have several questions: Do you plan to release the training dataset so users can retrain the model by incorporating new manually annotated data? The manuscript would benefit from quantifying segmentation performance by crop type. Measuring performance solely by computing time is insufficient, and quantitative metrics such as Dice scores on test holdout sets or cross-validation results (as performed by the nnUNet model) should be reported.
The current abstract describes pyRootHair as an "AI-powered software application to automate root hair trait extraction from images of plant roots grown on agar plates." This description needs to clarify that images were obtained via microscopy. Do you have insights on how the trained model performs across different microscope systems?
The manuscript requires additional clarification on the root straightening process using piecewise transformation, as this represents an important step in the measurement procedure. Please specify how this is performed and whether a specific algorithm or function from a library is used for the piecewise

affine transformation. For readers who are not computer vision specialists, a figure illustrating the measurement steps (segmentation â†' skeletonization â†' straightening â†' measurement) would be valuable.
Really minor comments: It would be helpful if the demo generated all plots by default, and Random Forest Classifier (RFC) is not included in the abbreviation list.
Overall, this represents solid work that addresses an important need in plant phenotyping research. The suggested clarifications will enhance both the scientific rigor and practical utility of the contribution.

Methods

Are the methods appropriate to the aims of the study, are they well described, and are necessary controls included? Choose an item.

Conclusions

Are the conclusions adequately supported by the data shown? Choose an item.

Reporting Standards

Does the manuscript adhere to the journal's guidelines on minimum standards of reporting? Choose an item.

Choose an item.

Statistics

Are you able to assess all statistics in the manuscript, including the appropriateness of statistical tests used? Choose an item.

Quality of Written English

Please indicate the quality of language in the manuscript: Choose an item.

Declaration of Competing Interests

Please complete a declaration of competing interests, considering the following questions:

- Have you in the past five years received reimbursements, fees, funding, or salary from an organisation that may in any way gain or lose financially from the publication of this manuscript, either now or in the future?
- Do you hold any stocks or shares in an organisation that may in any way gain or lose financially from the publication of this manuscript, either now or in the future?
- Do you hold or are you currently applying for any patents relating to the content of the manuscript?

- Have you received reimbursements, fees, funding, or salary from an organization that holds or has applied for patents relating to the content of the manuscript?
- Do you have any other financial competing interests?
- Do you have any non-financial competing interests in relation to this paper?

If you can answer no to all of the above, write 'I declare that I have no competing interests' below. If your reply is yes to any, please give details below.

I declare that I have no competing interests

I agree to the open peer review policy of the journal. I understand that my name will be included on my report to the authors and, if the manuscript is accepted for publication, my named report including any attachments I upload will be posted on the website along with the authors' responses. I agree for my report to be made available under an Open Access Creative Commons CC-BY license (http://creativecommons.org/licenses/by/4.0/). I understand that any comments which I do not wish to be included in my named report can be included as confidential comments to the editors, which will not be published.

Choose an item.

To further support our reviewers, we have joined with Publons, where you can gain additional credit to further highlight your hard work (see: https://publons.com/journal/530/gigascience). On publication of this paper, your review will be automatically added to Publons, you can then choose whether or not to claim your Publons credit. I understand this statement.

Yes Choose an item.
