# Supplementary material for: pyRootHair: Machine learning accelerated software for high-throughput phenotyping of plant root hair traits
Source: Gigascience. 2025 Nov 13;15:giaf141. doi: 10.1093/gigascience/giaf141 (PMC12824728; doi:10.1093/gigascience/giaf141)
Supplement: giaf141_Reviewer_3_Report_Original_Submission — Peter Pietrzyk -- 9/7/2025 [file giaf141_reviewer_3_report_original_submission.pdf]

Reviewer Report

Title: pyRootHair: Machine Learning Accelerated Software for High-Throughput Phenotyping of Plant Root Hair Traits

Version: Original Submission **Date:** 9/7/2025

Reviewer name: Peter Pietrzyk

Reviewer Comments to Author:

I really enjoyed reading this manuscript because it covers a small but very important niche in plant phenotyping. Root hairs play a crucial role in water and nutrient uptake, but phenotyping them remains a challenge. The manuscript introduces pyRootHair, an AI based image analysis tool for phenotyping of root hairs traits. The rationale of the work is clear: namely that manual root hair phenotyping is challenging and time consuming, while existing computational tools require input or long processing times. pyRootHair tries to alleviate this phenotyping bottleneck. The dataset is appropriate and validation against manual measurements shows good reliability of the method. The code and Jupyter notebooks are available on GitHub under MIT license. At the same time, some issues as stated below need to be addressed before publication.

Major comments

* Figure numbering and referencing: Across the manuscript, figure numbers are not always in the same order as they are referenced in the text. This creates unnecessary confusion for the reader and should be corrected. For example, the first referenced figure is Figure 3 in line 114. The same happens with supplementary figures. This creates confusion and should be corrected.
* Section 4 Data Description: This section only shortly describes the dataset of the wheat images, but does not include the images of the other species that were used for training and evaluation. These additional images are, however, used for analysis in Section 5.2 and 5.3. The manuscript would benefit from elaborating on the description of all data (e.g. different magnification, microscopes) used and include all images in this section.
* Lines 121-123: The manuscript mentions that images that contain a longer section of root would have a higher mean RHL. Is this a mistake or can the authors elaborate on this?
* Section 5.1 Variation in Root Hair Traits : The results are presented as a very large set of traits, but often without synthesis. At present, the section does not feel like an analysis. The authors should highlight and discuss the most biologically meaningful traits and better integrate the findings into a interpretation.
* The method to calculate RHD is described in section 5 Analysis section. I believe it would fit better into the Section 8 Methods. This would also improve the readability of section 5.
* I disagree with how the authors use the term plasticity. Plasticity usually relates to the change in a trait due to environmental conditions. Since no environmental factors were used in this study it should not be used. I think that developmental variability or heterogeneity are more suitable in the context of this manuscript.
* From Section 4 and Section 8.2 it appears as if different microscopes (Leica SP9 and Leica S9D) were

used. If this is a typo it should be corrected.
* Section 5.2 Adaptability: Are there any results for medicago, brachypodium and arabidopsis? If not, the authors should either add the results or do not state that they investigated these species.
* Section 5.4 Performance: Can you elaborate on what "extracted summary data" means?
* Section 6 Discussion: The critique of DIRT/Âµ could be framed more constructively. While pyRootHair emphasizes speed and throughput, DIRT/Âµ aims at accurate resolution of individual hairs. These represent different goals, and a fairer discussion would acknowledge the trade-offs rather than dismissing the alternative approach.
* Section 8.2 Image Acquisition: Most of this paragraph deals with plant material rather than the imaging process. This part should be moved to Section 8.1.
* Section 8.5 Workflow and Figure 1: The workflow was difficult to understand and there are a few mistakes in the text/figure. Steps 9-10 are omitted in the text and the description for Steps 12-15 are described as single step within one sentence. Please elaborate on that. In Figure 1 the text "Cubic spline interpolation on rotated root skeleton co-ordinates" is used between steps 9-10 as well as 10-11. I would also prefer to have a visual presentation of the steps to clarify some of the steps.
* Figure 3: The figure is overcrowded and very difficult to follow. The order of subplots does not match the order in which they are introduced in the text, which makes it hard for the reader to connect text with subplots. The figure would benefit from being split into multiple, thematically grouped panels, with subplots reordered to match the text.
* Figure 3/4: It would be also beneficial for easy comparison if the order of the traits is the same in both figures.
* The manuscript uses the word "extremely" several times (eight times in total), which reduces scientific objectivity. I recommend rephrasing with more precise or neutral wording (e.g., "computationally intensive" instead of "extremely computationally expensive").
Minor comments
* Mean RHL and average RHL are used interchangeably throughout the text and in Table 2. Please choose one and correct.
* Line 26: Eragostis tef is misspelled. Should be Eragrostis tef.
* Line 88: DIRTÎ¼ should be written DIRT/Î¼
* Line 118: Add the value of the correlation between mean RHL and maximum RHL: i.e. (Figure 4, R^2=???)
* Line 130: "Supplementary 2B" should be "Supplementary Figure 2B"
* Line 140: The term 'positive growth' is possibly unsuitable to describe the elongation zone. I would suggest: "…where root hairs increase length."
* Line 147: The sentence "All cultivars were imaged five days after germination." would fit better into the data description or methods sections
* Line 153: Figure 3M should be Figure 3N
* Line 181: Is the correlation between automated and manual measurements for RHL or mean RHL?
* Line 256 and other (plus Figure 2 caption): The term binary mask is used even though three classes are present. From my understanding a binary mask only has zeros and ones. It should therefore not be called binary.
* Section 8.6 Output: Be consistent with the format for arguments/flags (bold or not). E.g. -i/--input is

bold while --plot-summary is not
* Line 365: GWAS was not used as abbreviation in the text
* Table 1: Is DEU used for Germany? Explain the country abbreviations used in the caption
* Table 2: Why is "Avg RHL" used here? Could you be consistent with the rest of the text
* Figure 2: In the caption the vertical dashes lines are described as "maroon", but in the text (lines 141) they are described as "red". Also, the horizontal dashed line in the image looks purple, but in the caption it is described as "orange".

Methods

Are the methods appropriate to the aims of the study, are they well described, and are necessary controls included? Choose an item.

Conclusions

Are the conclusions adequately supported by the data shown? Choose an item.

Reporting Standards

Does the manuscript adhere to the journal's guidelines on [minimum standards of reporting?](minimum standards of reporting) Choose an item.

Choose an item.

Statistics

Are you able to assess all statistics in the manuscript, including the appropriateness of statistical tests used? Choose an item.

Quality of Written English

Please indicate the quality of language in the manuscript: Choose an item.

Declaration of Competing Interests

Please complete a declaration of competing interests, considering the following questions:

- Have you in the past five years received reimbursements, fees, funding, or salary from an organisation that may in any way gain or lose financially from the publication of this manuscript, either now or in the future?
- Do you hold any stocks or shares in an organisation that may in any way gain or lose financially from the publication of this manuscript, either now or in the future?
- Do you hold or are you currently applying for any patents relating to the content of the manuscript?

- Have you received reimbursements, fees, funding, or salary from an organization that holds or has applied for patents relating to the content of the manuscript?
- Do you have any other financial competing interests?
- Do you have any non-financial competing interests in relation to this paper?

If you can answer no to all of the above, write 'I declare that I have no competing interests' below. If your reply is yes to any, please give details below.

I declare that I have no competing interests

I agree to the open peer review policy of the journal. I understand that my name will be included on my report to the authors and, if the manuscript is accepted for publication, my named report including any attachments I upload will be posted on the website along with the authors' responses. I agree for my report to be made available under an Open Access Creative Commons CC-BY license (http://creativecommons.org/licenses/by/4.0/). I understand that any comments which I do not wish to be included in my named report can be included as confidential comments to the editors, which will not be published.

Choose an item.

To further support our reviewers, we have joined with Publons, where you can gain additional credit to further highlight your hard work (see: https://publons.com/journal/530/gigascience). On publication of this paper, your review will be automatically added to Publons, you can then choose whether or not to claim your Publons credit. I understand this statement.

Yes Choose an item.
